# Phylogenomic Analysis of micro-RNA Involved in Juvenile to Flowering-Stage Transition in Photophilic Rice and Its Sister Species

**DOI:** 10.3390/cells12101370

**Published:** 2023-05-12

**Authors:** Prasanta K. Dash, Payal Gupta, Rohini Sreevathsa, Sharat Kumar Pradhan, Tenkabailu Dharmanna Sanjay, Mihir Ranjan Mohanty, Pravat K. Roul, Nagendra K. Singh, Rhitu Rai

**Affiliations:** 1 ICAR-National Institute for Plant Biotechnology, Pusa Campus, New Delhi 110012, India; 2 ICAR-National Rice Research Institute, Cuttack 753006, India; 3 Department of Genetics & Plant Breeding (RRTTS, Jeypore), Odisha University of Agriculture and Technology, Bhubaneswar 751003, India

**Keywords:** stage transition, microsynteny, photophilic, *Oryza*, rice wild relatives, *MIRNA*s, *MIR172*

## Abstract

Vegetative to reproductive phase transition in phototropic plants is an important developmental process and is sequentially mediated by the expression of micro-RNA *MIR172*. To obtain insight into the evolution, adaptation, and function of *MIR172* in photophilic rice and its wild relatives, we analyzed the genescape of a 100 kb segment harboring *MIR172* homologs from 11 genomes. The expression analysis of *MIR172* revealed its incremental accumulation from the 2-leaf to 10-leaf stage, with maximum expression coinciding with the flag-leaf stage in rice. Nonetheless, the microsynteny analysis of *MIR172*s revealed collinearity within the genus *Oryza,* but a loss of synteny was observed in (i) *MIR172*A in *O. barthii* (AA) and *O. glaberima* (AA); (ii) *MIR172*B in *O. brachyantha* (FF); and (iii) *MIR172*C in O. *punctata* (BB). Phylogenetic analysis of precursor sequences/region of *MIR172* revealed a distinct tri-modal clade of evolution. The genomic information generated in this investigation through comparative analysis of *MIRNA,* suggests mature *MIR172*s to have evolved in a disruptive and conservative mode amongst all *Oryza* species with a common origin of descent. Further, the phylogenomic delineation provided an insight into the adaptation and molecular evolution of *MIR172* to changing environmental conditions (biotic and abiotic) of phototropic rice through natural selection and the opportunity to harness untapped genomic regions from rice wild relatives (RWR).

## 1. Introduction

Micro-RNAs (*MIRNA*s) function as post-transcriptional regulators of gene expression in eukaryotes [1]. In plants under a given environmental condition, *MIRNA*s perform a host of regulatory functions, and one important regulation is phase transitions leading to plant morphogenesis and development [2,3,4,5]. Such phase transitions mark cardinal changes in plant development and are mediated by sequentially expressed *MIRNA*s [6]. Stage transitions are cardinal and necessary changes in the plant developmental processes, and these transitions are mediated by sequentially expressed miRNAs [6]. *MIRNA172* is one such family of MIRNA that is involved in phase transition during plant development. Vegetative phase changes in Arabidopsis and maize are controlled by the sequential activity of *miR156* and *MIR172* [7]. Although *miR156* is highly expressed during early developmental stages, *MIR172* is highly expressed during later stages of development [7,8,9]. In plants such as *Acacia confusa*, *A. colei*, *Hedera helix*, *Eucalyptus globulus*, and *Quercus acutissima*, contrasting expression patterns of *miR156* and *MIR172* and their target genes were observed [10].

Inter alia, *MIR172* is one such family of *MIRNA* that is sequentially involved in the vegetative to reproductive phase transition in Arabidopsis and maize [7]. *MIR172* is highly expressed during later stages of plant development [7,8,9] and, functionally, one of the first plant *MIRNA* to be identified in Arabidopsis [11]. In most plants, it is 22-nucleotide long and is highly conserved. However, the number of precursor-*MIR172*, mature *MIR172*, and the target genes exhibit diversity in different plant species. In rice, two mature *MIR172*s are encoded by four precursor-*MIR172*s with several homologs (similar due to descent from a common ancestor) and homeologs (homologs resulting from allopolyploidy).

Functionally, *MIR172* is ubiquitous and one of the earliest plant miRNA genes to be identified by small-RNA cloning and sequencing in Arabidopsis [11]. In *Arabidopsis*, five pri-*MIR172*s encode three mature *MIR172*s, and these *MIR172*s repress the expression of six members of the APETALA 2 (AP2)-like family of transcription factors, three TARGET OF EAT (TOE) proteins, and SCHLAFMUTZE (SMZ) and its paralog SCHNARCHZAPFEN (SNZ) [12]. In maize, five pri-*MIR172*s encode only one mature *MIR172*, which represses the expression of six members of the *AP2*-like family of transcription factors and TS6-GN2230 [13]. In rice, four pri-*MIR172*s encode two mature *MIR172*s and repress five members of the AP2-like transcription factors [13,14]. In *Populus trichocarpa*, nine pri*-MIR172*s encode four mature *MIR172*s with six target genes [15].

Beyond its cardinal role in the transition from the vegetative to the reproductive stage, *MIR172* is also involved in a repertoire of developmental processes in plants, such as the determination of stem cell fate [16], developmental timing [12,17,18,19,20], sex determination [21], flowering [22], fruit growth [23,24], spike architecture and grain threshability in wheat [25,26], tuberization in potatoes [27], and nodulation in soybeans [28]. Response to abiotic stress in *Arabidopsis* [29] and biotic stress resistance in tomatoes [30] are also affected by *MIR172* expression. Recently it was observed that auxins also modulate *MIR172* [1] activity to affect plant morphogenesis.

Grain production in field crops has doubled in the last decades due to the “Green revolution” [31], along with advances in functional [32,33,34,35] and structural [36,37,38,39,40,41,42] genomics. Further, marker-assisted back-cross breeding (MABB) imparted biotic [43,44,45,46] and abiotic [47,48,49,50] stress tolerance, ultimately leading to higher grain yield. However, we still need to improve food production to maintain the demand-supply balance. In this regard, wild relatives of a crop are more robust genomic sources of traits/genes than their domesticated counterparts in terms of disease tolerance and plant architecture [5]. Agriculturally important crops [47,48,49] are positively photophilic and phototropic and are constantly exposed to changing environmental conditions during their lifetime. Environmental stresses, such as biotic and abiotic stresses, cause adverse effects on growth, development, and yield. However, their wild relatives are reservoirs of untapped genomic resources to be utilized to develop/breed improved cultivars to combat biotic/abiotic threats posed by climate change [51]. Rice is the world’s second most-produced [52] staple food crop, and its evolution from grasses has had an immense impact on human civilization catering to daily nutrition [53] globally. It belongs to the genus *Oryza* (family poaceae), which comprises 24 species, among which *Oryza sativa* L. and *Oryza glaberrima* S. are cultivated, and the remaining species are wild relatives spread all over the world [54]. Wild *Oryza* species or relatives (RWRs) are adapted to diverse habitats and are more robust compared to cultivated varieties in terms of stress tolerance [55]. RWRs are a rich resource of unexplored genes for breeding improved cultivated rice to combat the threats posed by increasing population and climate change [51]. Previous studies targeting the regulation/expression of genes targeted by *MIR172* in poaceae have been accomplished, but studies focusing on evolution, content, genetic diversity, and structure of genes embedded in the *MIRNA172* locus per se are lacking. In this endeavor, we employed a comparative genomics approach to identify homologs of *MIRNA172* in rice and its wild relatives to gain insight into the expression and evolution of the region in and around *MIR172*. Our study revealed the conservative and disruptive evolution of the *MIR172* gene family in various cultivated/wild grasses and supplanted genomic information to be harnessed from RWR to design a designer crop tolerant to abiotic/biotic stresses.

## 2. Materials and Methods

### 2.1. Sequence Retrieval of MIR172 Homologs

MiRBase 22.1 (http://www.mirbase.org/, accessed on 10 March 2021) was used to retrieve the mature and precursor sequences of *MIR172* from *Oryza sativa*, *Sorghum bicolor*, *Zea mays*, *Triticum aestivum*, and *Arabidopsis thaliana* [56]. *Oryza sativa MIRNA* precursor sequences were used as the query to execute BLASTN (Local BLAST) against the genome sequences of *O. glaberrima*, *O. glumaepatula*, *O. rufipogon*, *O. punctata*, *O. barthii*, and *O. brachyantha* that are available in the Gramene to extract each plant’s precursor sequence, the *MIRNA* precursor sequences of sorghum, maize, and Arabidopsis were utilized as the query. Based on the score, e-value (lowest), and percentage identity (highest), high-scoring pairings (HSP) for *MIR172* were found. These HSP were then chosen for comparative genomic study in order to comprehend the microsynteny, organizational structure, and evolutionary trend of *MIR172* in domesticated and wild grasses. As an outlier for determining the evolutionary trend, the Arabidopsis *MIR172* sequence from TAIR (https://www.arabidopsis.org/, accessed on 10 March 2021) was used.

### 2.2. MIRNA Precursor Sequence Analysis

Using the MAFFT version 7.271 program [57] with the L-INS-I approach and output in Phylip format, multiple sequence alignment of *MIR172* precursor and mature sequences was carried out in seven *Oryza* spp., maize, sorghum, and Arabidopsis. The aligned sequences were scored for similarity using ESPRIPT 3.0 [58] (https://espript.ibcp.fr/ESPript/ESPript/, accessed on 12 March 2023) with the default settings.

### 2.3. Microsynteny Analysis

*O. sativa*, *O. rufipogon*, *O. glaberrima*, *O. barthii*, *O. glumaepatula*, *O. brachyantha*, *O. punctata*, *Zea mays*, and *Sorghum bicolor* genomic sequences were obtained from Gramene database, and *Arabidopsis thaliana* genomic sequences were obtained from TAIR (https://www.arabidopsis.org/, accessed on 15 March 2021) (Supplementary Table S1). The reference plant for the microsynteny investigation was *Oryza sativa*. Genes were predicted using the FGENESH tool from Molquest II (http://www.molquest.com/molquest.phtml?group=index&topic=gfind, accessed on 18 March 2021) [59] with the default parameters. For all rice species, *Oryza sativa* was used as the default template, and the genomes of wheat, maize, and sorghum were employed as templates for their corresponding genomes. Blast2GO software (version 5.2.5) (https://www.blast2go.com/, accessed on 19 March 2021) was used to perform functional annotation and genomics analysis on genes predicted by FGENESH in the 100 kb area [60]. Functionally annotated genes from the 100 kb region of *MIRNA*s were enlisted from each species and used for microsynteny analysis using BLAST2GO analysis.

The database for microsynteny analysis, as well as the blastp for calculating synteny block input, were created using the NCBI BLAST-2.11.1+ software (makeblastdb and blastp). The query for the makeblastdb script comprised four sets of proteins (one set per *MIR172*A, B, C, and D) predicted by the MolQuest program in 100 kb genomic regions of 10 genomes under study in the previous stage. The blastp settings were blastp-outfmt 8-e-value 1 × 10^−10^-max target seqs [5]. Synteny blocks for each *MIRNA* were computed using the blast output and GFF annotations of seven *Oryza*, two non-*Oryza*, and arabidopsis as input. The MCScanX tool [61] was used to determine the interspecies syntenic blocks with the following parameters: Match-score, final score = match score + num gaps × gap penalty (default: 50); gap-penalty, gap penalty (default: 1); match-size, the number of genes required to call a collinear block (default: 5); E-value, alignment significance 1 × 10^−5^; max-gaps, maximum gaps allowed (default: 25); and overlap-window, maximum distance 10,000 (number of nucleotides between genes) (default: 5) as well as collinear block patterns: 1 inter-species. The method found two or more species that shared a pairwise synteny block with at least five genes shared and an E-value of 1 × 10^−10^ in a maximum range of 10,000 nucleotides. The MCScanX software circle plotter was used to generate the figures. After identifying the collection of synteny blocks, in-house scripts were created to sub-set the MCScanX collinearity output file. Circos (Version 0.69-9., http://circos.ca/, accessed on 25 March 2021) [62] was used to generate circular graphs.

### 2.4. Plant Material, PCR, and Sequencing of Precursor MIR172

Seeds of *O. sativa*, *O. rufipogon*, *O. glaberrima*, *O. barthii*, *O. glumaepatula*, *O. brachyantha*, and *O. punctata* were procured from AICRP on rice (OUAT), and individual collections of rice breeders, seeds of *Zea mays* and *Sorghum bicolor*, were obtained from the division of Genetics, IARI; seeds of Arabidopsis were available in the laboratory. Plants were sown on moistened germination paper for five days, and after initial growth, the seedlings were transferred to 12-inch pots containing a mixture of cocopeat and sand (1:1) for completion of growth. Fresh tissues were harvested from pot-grown plants for amplification of *MIRNA*s and downstream experiments. Amplification and sequencing of each of the four *MIR172* homologs from *O. sativa*, *O. rufipogon*, *O. glaberrima*, *O. barthii*, *O. glumaepatula*, *O. brachyantha*, *O. punctata*, *Zea mays*, *Sorghum bicolor*, and *Arabidopsis thaliana* were accomplished with *Triticum aestivum* as an outlier since no homologs of *MIR172* were identified in wheat by *in-silico* analysis. Using the CTAB method, genomic DNA was isolated from ten plant species and used as a template for amplification of *MIR172* A, B, C, and D precursor sequences using specific primers (Supplementary Table S2) in a master-cycler using high-fidelity DNA polymerase. The PCR reaction conditions were as follows: 30 s at 98 °C, 30 cycles of 10 s at 98 °C, 15 s at different annealing temperatures, and 10 s at 72 °C. For validation, the amplified PCR products were sequenced and aligned with pre-existing sequences.

### 2.5. Expression Analysis of Mature MIR172

Total *MIRNA* was extracted from the stage staggered tissues of seven *Oryza* spp., such as 2 leaf-shoot, 2 leaf-root, 4 leaf-shoot, 10 leaf-shoot, 10 leaf-shoot apical, 10 leaf-root, flag leaf, booting panicle, panicle (0.5 cm), panicle (0.5–1 cm), panicle (1–2 cm), panicle (2–4 cm), sorghum (3 leaf-shoot, 3 leaf-root, 5 leaf-shoot, 5 leaf-root, growing point, flower that has not yet bloomed, complete flower-blooming and G-blooming), maize (2 leaf-shoot, 2 leaf-root, 4 leaf-shoot, 10 leaf-shoot, 10 leaf-root, flag leaf, tassel, and silk), and Arabidopsis (2 rosette leaf-shoot, 2 rosette leaf-root, 4 leaf rosette-shoot, 10 leaf rosette-shoot, 10 leaf rosette-root, complete rosette growth, inflorescence, floral bud, and open flower) using a commercial *MIRNA* isolation kit as per manufacturer’s instruction. Using the Mir-XTM *MIRNA* First-Strand synthesis kit, cDNA was synthesized from the extracted *MIRNA*. Real-time PCR (qRT-PCR) was used to examine the expression of mature *MIR172* using the Mir-X *MIRNA* qRT-PCR SYBR Kit with the mRQ 3′ universal reverse primer supplied with the kit, the species-specific mature *MIR172* forward primer (Supplementary Table S3), and a reference dye (ROX) according to the manufacturer’s protocol. In 96-well qPCR compatible plates, the following cycles were used: initial denaturation for 5 min at 95 °C, 45 cycles of 15 s at 95 °C, 30 s at 60 °C, and 30 s at 72 °C. U6 was used as an endogenous control to normalize the *MIRNA* expression level. The results were presented as the mean of three biological replicates, with three technical replicates for each biological repeat.

### 2.6. Phylogenomic Analysis of Precursor Sequences

*MIR172* homologs’ precursor sequences, along with 500 bp upstream sequences, were retrieved from the Gramene database. Synteny/evolutionary link between different *MIRNA*s was deduced by using the Gramene database (Supplementary Table S1). Clustal Omega [63] was used to conduct multiple sequence alignment for the individual *MIRNA*s, and MEGA10 [64] was used to construct an un-rooted tree using the Maximum-Likelihood (ML) technique. The Nearest Neighbor Interchanges (NNIs) method [65] was used to search for tree topology. For *MIR172*, the Tamura 3-parameter model with a discrete Gamma distribution (+G) and 5 rate categories substitution was used. The gamma shape parameter was directly determined from the data, and 1000 bootstrap replicates were used in the procedure. The proportion of invariable sites was fixed. The tree was generated in the Newick format. I-TOL (http://itol.embl.de/, accessed on 28 March 2021) produced a graphical representation of the phylogenetic tree.

### 2.7. Test of Neutrality

The neutrality of *MIRNA* sequence polymorphisms among poaceae was assessed using DnaSP v5.10 [66] and neutrality tests such as Tajima’s D [67] and Fu and Li’s F [68] on four distinct *MIRNA* loci, namely *MIR172*A, *MIR172*B, *MIR172*C, and *MIR172*D.

## 3. Results

### 3.1. Identification of MIR172 Homologs

**Figure 1:**
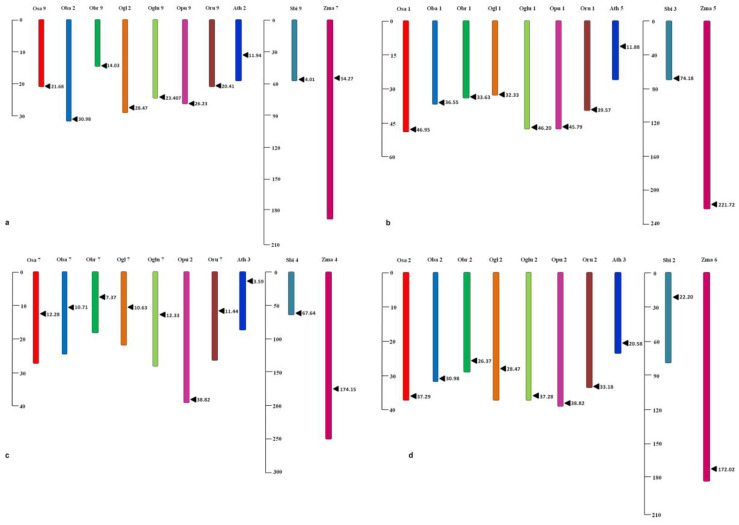
Identification and location of *MIR172* and its homologs amongst analyzed poaceae members. Genomic location of (**a**) *MIR172*A (Scales: 0–30 Mb and 0–210 Mb); Osa9-*Oryza sativa* chr 9, Ogl2-*Oryza glaberrima* chr 2, Oba2-*Oryza barthii* chr 2, Oglu9-*Oryza glumaepatula* chr 9, Obr9-*Oryza brachyantha* chr 9, Oru9-*Oryza rufipogon* chr 9, Opu9-*Oryza punctata* chr 9, Zma7-*Zea mays* chr 7, Sbi9- *Sorghum bicolor* chr 9, Ath2-*Arabidopsis thaliana* chr 2; (**b**) *MIR172*B (Scales: 0–60 Mb and 0–240 Mb); Osa1-*Oryza sativa* chr 1, Ogl1-*Oryza glaberrima* chr 1, Oba1-*Oryza barthii* chr 1, Oglu1-*Oryza glumaepatula* chr 1, Obr1-*Oryza brachyantha* chr 1, Oru1-*Oryza rufipogon* chr 1, Opu1-*Oryza punctata* chr 1, Zma5-*Zea mays* chr 5, Sbi3-*Sorghum bicolor* chr 3, Ath5-*Arabidopsis thaliana* chr 5; (**c**) *MIR172*C (Scales:0–40 Mb and 0–300 Mb); Osa7-*Oryza sativa* chr 7, Ogl7-*Oryza glaberrima* chr 7, Oba7-*Oryza barthii* chr 7, Oru7-*Oryza rufipogon* chr 7, Oglu7-*Oryza glumaepatula* chr 7, Opu2*-Oryza punctata* chr 2, Obr7-*Oryza brachyantha* chr 7, Zma4-*Zea mays* chr 4, Sbi4-*Sorghum bicolor* chr 4, Ath3-*Arabidopsis thaliana* chr 3; (**d**) *MIR172*D (Scales: 0–40 Mb and 0–210 Mb) Osai2-*Oryza sativa* homeolog i chr 2, Osaii2-*Oryza sativa* homeolog ii chr 2, Ogl2-*Oryza glaberrima* chr 2, Oba2-*Oryza barthii* chr 2, Oglu2-*Oryza glumaepatula* chr 2, Obr2-*Oryza brachyantha* chr 2, Oru2-*Oryza rufipogon* chr 2, Opu2-*Oryza punctata* chr 2, Zma6-*Zea mays* chr 6, Sbi2-*Sorghum bicolor* chr 2, Ath3-*Arabidopsis thaliana* chr 3; on chromosomes of different poaceae members.

*MIR172* homologs were identified in all seven *Oryza* spp., sorghum, maize, and Arabidopsis except wheat, as no precursor of *MIR172* could be identified in wheat using its miRBase database. In rice and its wild relatives, four homologs of *MIR172,* i.e., *MIR172* (A–D), were identified. These homologs are located on different chromosomes throughout the rice genome (Table 1). In sorghum, a total of six homologs of *MIR172,* i.e., *MIR172* (A–F), were identified, whereas, in maize and Arabidopsis, five homologs of *MIR172,* i.e., *MIR172* (A–E) were identified. Since we used *sativa* as a reference for genome structure and evolution analysis, only four homologs corresponding to rice, i.e., *MIR172* (A–D), were included in the study. Chromosomal locations and coordinates of each homolog (Table 1, Figure 1a–d) and the number of predicted homologs per *MIR172* are provided in Table 2. The presence of four homologs in rice, five in maize and Arabidopsis, and six in sorghum was confirmed by the BLAST result in this study. All the homologs of *MIR172* viz., *MIR172A, B*, *C*, and *D* were successfully amplified, and the nucleotide sequences have been submitted as Supplementary Data S1.

### 3.2. Expression Analysis of MIR172

**Figure 2:**
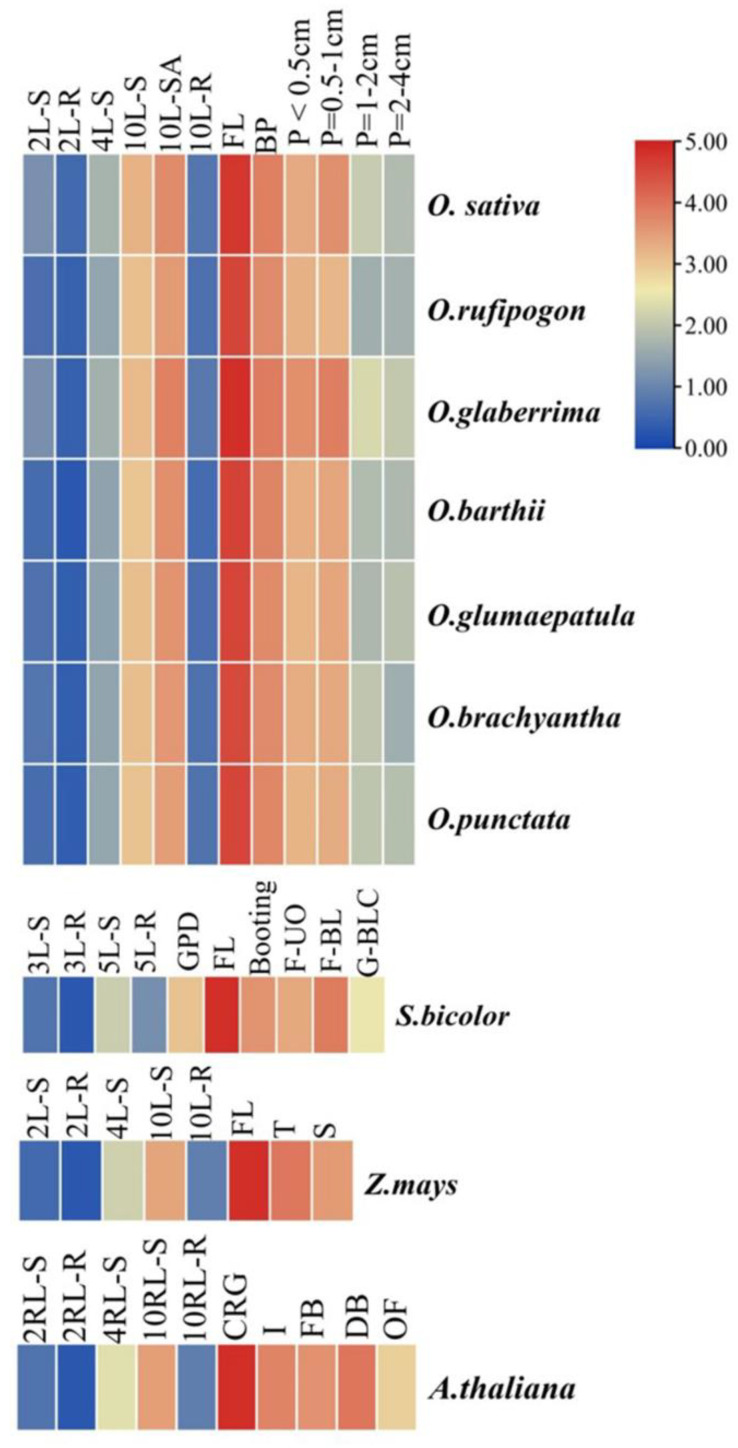
Expression profiling of *MIR172* in rice, other cereals, and A. thaliana. Expression pattern obtained by qRT-PCR of each block shows log_2_-fold expression in different stage-specific developmental tissue. Details of specific stages used for each plant species: *Oryza* spp. (2LS-2 leaf-shoot, 2LR-2 leaf-root, 4LS-4 leaf-shoot, 4LR-4 leaf-root, 10LS-10 leaf-shoot apical, 10LR-10 leaf-root, FL-flag leaf, BP-booting panicle, p (<0.5 cm)-panicle (<0.5 cm), p (0.5–1 cm)-panicle (0.5–1 cm), p (1–2 cm)-panicle (1–2 cm) and p (2–4 cm)-panicle (2–4 cm)); *Sorghum bicolor* (3LS-3 leaf-shoot, 3LR-3 leaf-root, 5LS-5 leaf-shoot, 5LR-5 leaf-root, GPD-growing point differentiation, FL-flag leaf, BP-booting panicle, F-flower not yet bloomed, F-blooming-flower-blooming, G-blooming complete); *Zea mays* (2LS-2 leaf-shoot, 2LR-2 leaf-root, 4LS-4 leaf-shoot, 10LS-10 leaf-shoot, 10LR-10 leaf-root, FL-flag leaf, T-Tassel and S-silk) and *Arabidopsis thaliana* (2RLS-2 rosette leaf-shoot, 2RLR-2 rosette leaf-root, 4RLS-4 rosette leaf -shoot, 10RLS-10 leaf rosette-shoot, 10RLR-10 rosette leaf -root, CRG-complete rosette growth, I-Inflorescence, FB-floral bud, and OF-open flower).

*MIR172* is known to play a cardinal role in vegetative to reproductive-stage transition in plants [7]. To determine the variations in the expression pattern of *MIR172* during development, an expression profile of mature *MIR172* was compared in six RWR and other poaceae members. Accumulation of mature *MIR172* increased with the growth of the plant, gradually increasing from the 2-leaf stage (0.582-fold) to the 10-leaf stage (8.54-fold), reaching its maximum in the flag-leaf stage in rice (25.97-fold, Figure 2). However, there was no increase in *M1IR172* expression in roots in rice. Similarly, in sorghum, the expression of *MIR172* increased from the 3-leaf stage (0.934-fold) to growth point differentiation (7.35-fold), reaching a maximum in the flag-leaf stage (24.9-fold, Figure 2). A similar pattern was observed in maize, with maximum expression in the flag leaf (23.9-fold). In the case of Arabidopsis, the expression level gradually increased from the 2-rosette leaf stage (0.745-fold) to the complete rosette growth stage (22.615-fold, Figure 2). Flag leaf or complete rosette growth marks the transition stage between the vegetative and reproductive stages. During the reproductive growth phase, increased accumulation of *MIR172* was observed in the booting panicle stage in rice (13.465-fold), maize (13.546-fold), and sorghum (10.918-fold); and developing inflorescence in Arabidopsis (11.22-fold). Expression of *MIR172* gradually decreased during panicle development. Mature *MIR172* exhibited a similar expression profile in all *Oryza* spp. with minor variations in the magnitude of expression among different species (Figure 2).

### 3.3. Conservation and Divergence in Mature and Precursor Sequence of MIR172

**Figure 3:**
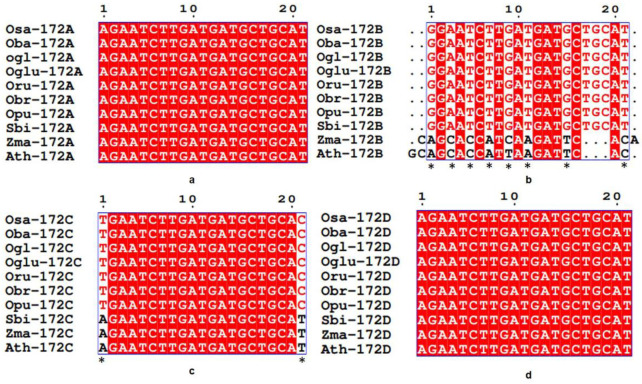
Multiple sequence alignment of mature *MIR172* exhibiting conserved and diverged regions. (**a**) Highly conserved mature *MIR172*A sequences amongst poaceae members and Arabidopsis, (**b**) Prevalence of SNPs and InDels (insertions/deletions) detected at multiple positions in Zma-172B and At-172B by multiple sequence alignment of mature *MIR172*B. Conserved mature sequence of *MIR172*B in seven *Oryza* spp. * Denotes position of single nucleotide polymorphism. (**c**) Sequence alignment of mature *MIR172*C of all poaceae members vis-a-vis Arabidopsis shows the prevalence of substitutions. Single nucleotide substitution where ‘T’ in rice is replaced by ‘A’ in *S. bicolor*, *Z. mays,* and Arabidopsis while ‘C’ in rice is replaced by ‘T’ in *S. bicolor*, *Z. mays,* and Arabidopsis; in highly conserved mature *MIR172*C of all poaceae members and Arabidopsis and (**d**) Sequence alignment of mature *MIR172*D of all poaceae members vis-a-vis Arabidopsis. Osa-*Oryza sativa*, Oba-*Oryza barthii*, Ogl-*Oryza glaberrima*, Oglu-*Oryza glumaepatula*, Oru-*Oryza rufipogon*, Obr-*Oryza brachyantha*, Opu-*Oryza punctata*, Sbi-*Sorghum bicolor*, Zma-*Zea mays*, Tae-*Triticum aestivum*, Ath-*Arabidopsis thaliana*.

Mature *MIR172A* and *MIR172D* of all the poaceae members and Arabidopsis were found to be highly conserved (Figure 3a,d), while mature *MIR172B* homologs of all the seven *Oryza* spp and sorghum were highly conserved. However, in mature *MIR172B* sequence of maize and Arabidopsis, multiple substitutions at 1^st^ (G→A), 3^rd^ (A→C), 5^th^ (T→C), 7^th^ (T→A), 9^th^ (G→C in maize and G→T in Arabidopsis), 11^th^ (T→A), 15th (G→T) and 21^st^ (T→C) positions were detected along with deletions and insertions (Figure 3b). A single nucleotide insertion was observed at the zero position in Arabidopsis and at the 22^nd^ position in maize. Three nucleotide (TGC) deletions were observed in Arabidopsis and maize between the 16^th^ and 20^th^ positions (Figure 3b). *MIR172C* homologs revealed the highly conserved nature of all the rice *MIR172C* homologs. In the case of sorghum*,* maize, and Arabidopsis, two base substitutions at the 1^st^ (T→A) and 21^st^ (C→T) positions were observed (Figure 3c).

**Figure 4:**
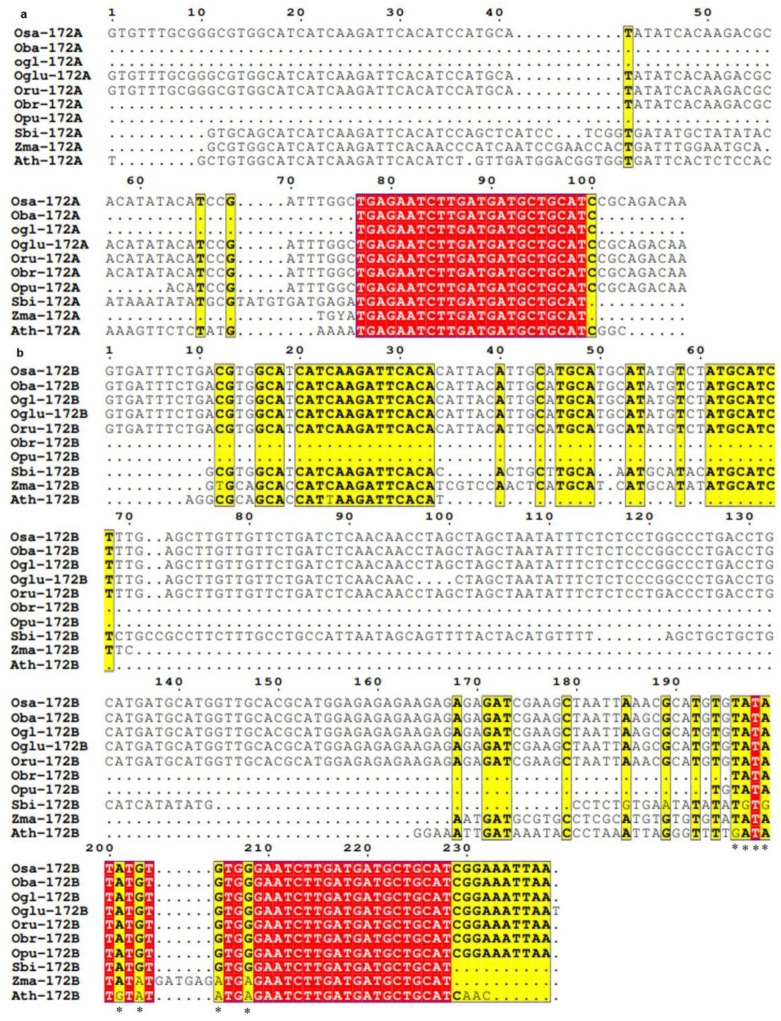
Multiple sequence alignment for elucidation of conservation and divergence in *MIR172*A and B precursor region (**a**) Precursor sequence alignment of *MIR172*A across poaceae; and (**b**) Precursor sequence alignment of *MIR172*B across poaceae. The sequences in the red box are regions of the sequences that are highly conserved among all the species included in the study. * denotes the sites for single nucleotide polymorphism. Osa-*Oryza sativa*, Ogl-*Oryza glaberrima*, Oba-*Oryza barthii*, Oru-*Oryza rufipogon*, Oglu-*Oryza glumaepatula*, Opu-*Oryza punctata*, Obr-*Oryza brachyantha*, Zma-*Zea mays*, Sbi-*Sorghum bicolor*, Ath-*Arabidopsis thaliana*.

Multiple sequence alignment of precursor sequences of *MIR172* revealed the non-occurrence of SNPs in the *MIR172A* precursor sequences of all poaceae members and Arabidopsis. However, insertions and deletions were detected in almost all poaceae species under study. An insertion of 11 nucleotides (ATCCGAACCAC) was observed between the 41^st^ and 42^nd^ positions in maize and Arabidopsis. Further, an eight-nucleotide (ATCCTCGG) insertion was observed between the 41^st^ and 42^nd^ positions in sorghum. A five nucleotides (TATGT) insertion between the 69^th^ and 70^th^ positions was also observed in sorghum. A deletion of the first 76 nucleotides (GTGTTTGCGGGCGTGGCATCATCAAGATTCACATCCATGCATATATCACAAGACGCACATATACATCCGATTTGGC) was observed in *barthii* and *glaberrima*, while deletion of first 41 nucleotides (GTGTTTGCGGGCGTGGCATCATCAAGATTCACATCCATGCA) was observed in *rufipogon.* Similarly, the first 62 nucleotide (GTGTTTGCGGGCGTGGCATCATCAAGATTCACATCCATGCATATATCACAAGACGCACATAT) deletion was also observed in *punctata*. A nine nucleotide (CGCAGACAA) deletion was also observed at the 100th position in *barthii* and *glaberrima*. A 17-nucleotide deletion (CACATATACATCCGATT) between the 55^th^ and 73^rd^ position was observed in maize. In addition to this, a ten-nucleotide (CCGCAGACAA) deletion at the 99^th^ position in sorghum and maize and a six-nucleotide (AGACAA) deletion at the 103^rd^ position in Arabidopsis was also observed (Figure 4a).

In *MIR172B* precursors, specific single nucleotide substitution was detected at the 203^rd^ position, where G was replaced with A; at the 205^th^ position, G was replaced with A, and at the 208^th^ position, G was replaced with A was observed in maize, and Arabidopsis in comparison to rice. Specific single base substitutions were also observed in sorghum at the 197^th^ (A→G) and the 199^th^ (A→G) positions. In Arabidopsis, similar single nucleotide substitutions were observed at the 196^th^ (T→G) and 201^st^ (T→G) positions. In addition to single nucleotide substitutions, insertions and deletions at multiple locations were also observed. A genome-specific two-nucleotide (CC) insertion between the 71^st^ and 72^nd^ positions was observed in sorghum. An insertion of six nucleotides (GATGAG) between the 204^th^ and 205^th^ positions was observed in maize. A deletion of the first 195 nucleotides (GTGATTTCTGACGTGGCATCATCAAGATTCACACATTACATTGCATGCATGCATATTCTATGCATCTTTGAGCTTGTTGTTCTGATCTCAACAACCTAGCTAGCTATATTTCTCTCCTGGCCCTGACCTGCATGATGCATGGTTGCACGCATGGAGAGAGAAGAGAGAGATCGAAGCTAA TT AAACGCATGTG) in *brachyantha* and the first 193 nucleotides (GTGATTTCTGACGTGGCATCATCAAGATTCACACATTACATTGCATGCATGCATATGTTATGCATCTTTGA GCTTGTTGTTCTGATCTCAACAACCTAGCTAGCTAATATTTCTCTCCTGGCCCT GA CCTGCATGATGCATGGTTGCACGCATGGAGAGAGAAGAGAGAGATCGAAGCTA ATTAAACGCATG) in *punctata* was observed. The first ten nucleotides (GTGATTTCTG) were also found to be deleted in sorghum and maize, and the first eight nucleotides (GTGATTTC) in Arabidopsis were found to be deleted. In sorghum, (ATTAC) deletions between the 34^th^ and 40^th^, (TG) deletions between the 49^th^ and 52^nd^, (TCTCCTG) deletions between the 114^th^ and 122^nd^, (GTTGCACGCATGGAGAGAGAAGAGAGAGATCGAAGC) deletions between 143^rd^ and 180^th^ and (CGGA) at 228^th^ positions were observed. In maize, 50^th^ and 52^nd^ positions (G), 70^th^ and 168^th^ (GAGCTTGTTGTTCTGATCTCAACAACCTAGCTAGCTAATATTTCTCTCTGGCCCTGACCTGCATGATGCATGGTTGCACGCATGGAGAGAGAA GAGAG) deletions between and (CGGA) deletions between 228^th^ were observed. In Arabidopsis, single deletion of 130 nucleotides (CATTACATTGCATGCATGCATATGTCTATGCATCTTTGAGCTTGTTGTTCTGATCTCAACAACCTAGCTAGCTAATATTTCT CTCCTGGCCCTGACCTGCATGATGCATGGTTGCACGCATGGAGAGAGA) between the 34^th^ and 164^th^ position was observed (Figure 4b).

**Figure 5:**
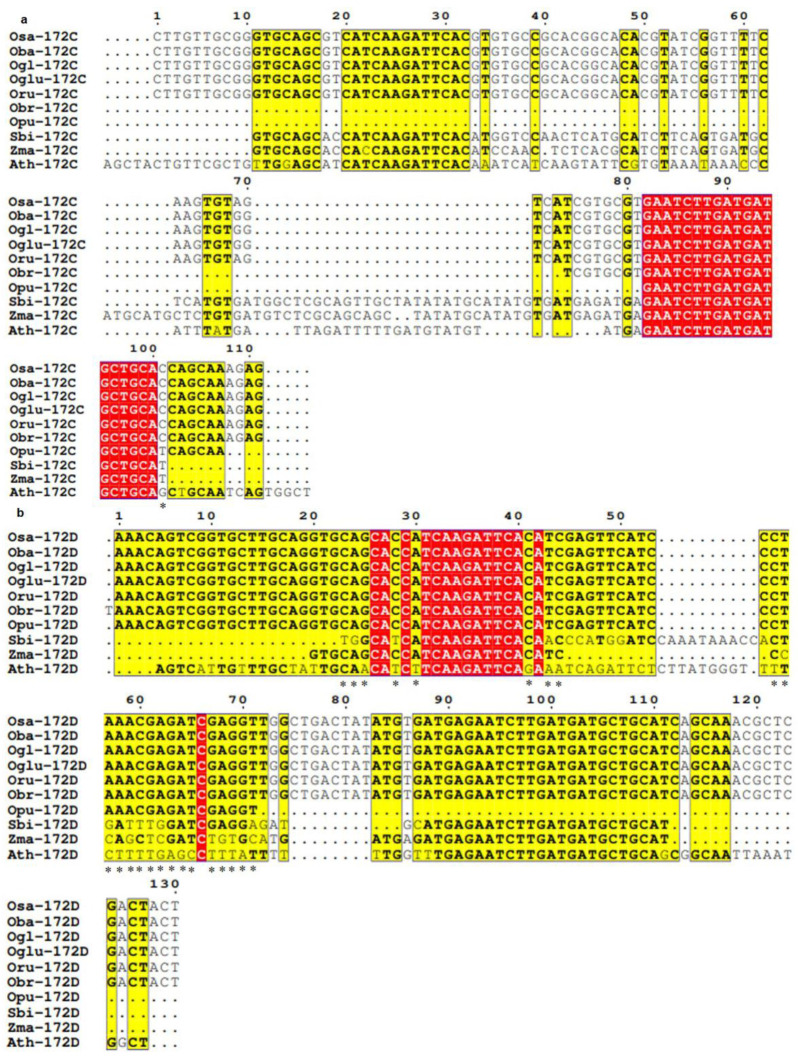
Multiple sequence alignment discerns conservation and divergence in the precursor region of *MIR172*C and D. (**a**) *MIR172*C precursor sequence alignment in poaceae; (**b**) *MIR172*D precursor sequence alignment in poaceae. The sequences in the red box are regions of the sequences that are highly conserved among all the species included in the study. * denotes the sites of single nucleotide polymorphism. Osa-*Oryza sativa*, Ogl-*Oryza glaberrima*, Oba-*Oryza barthii*, Oru-*Oryza rufipogon*, Oglu-*Oryza glumaepatula*, Opu-*Oryza punctata*, Obr-*Oryza brachyantha*, Zma-*Zea mays*, Sbi-*Sorghum bicolor*, Ath-*Arabidopsis thaliana*.

Multiple sequence alignment of *MIR172C* precursor sequences revealed the occurrence of only one SNP. Single nucleotide polymorphism was observed at the 101^st^ position, where ‘T’ in rice was replaced with ‘G’ in Arabidopsis. Although many insertions and deletions were also observed, prominent amongst them is the insertion of five nucleotides (AGCTA) at the zero position, 19 nucleotides (TTAGATTTTTGATGTATGT) between the 70^th^ and 71^st^ position, and five nucleotides (TGGCT) at 111^th^ position in Arabidopsis. Sorghum, a seven-nucleotide insertion (ATGCATG) between the 62^nd^ and 63^rd^, and 28 nucleotides insertion (TGGCTCGCAGTTGCTATATATGCATATG) between the 70^th^ and 71^st^ positions were observed. A deletion of the first 73 nucleotides (CTTGTTGCGGGTGCAGCGTCATCAAGATTCACGTGTGCCGCACGGCACACGTATCGGTTTTCAAGTGTAGTCA) and the first 81 nucleotides (CTTGTTGCGGGTGCAGCGTCATCAAGATTCACGTGTGCCGCACGGCAC ACGTATCGGTTTTCAAGTGTAGTCATCGTGCGT) was observed in *brachyantha* and *punctata,* respectively. Additionally, the deletion of four nucleotides (AGAG) at the 107^th^ position was observed in *punctata*. A deletion of ten nucleotides (CTGCAATCAG) at the 101st position was observed in sorghum and maize (Figure 5a).

In *MIR172D* precursors, genome-specific single nucleotide substitutions were detected in sorghum at 23^rd^ (C→T), 24^th^ (A→G), 28^th^ (C→T), 43^rd^ (T→A), 57^th^ (A→G) and 71^st^ (T→A) positions. Four consecutive single nucleotide substitutions were detected at the 59^th^ (A→T), 60^th^ (C→T), 61^st^ (G→T), and 62^nd^ (A→G) positions in *S. bicolor*. In maize, two consecutive nucleotide substitutions were observed at the 56^th^ (T→C) and 57^th^ (A→C) positions and at the 61^st^ (G→T) and 62^nd^ (A→C) positions. In *Z. mays,* two SNPs at 59^th^ (A→G) and 71^st^ (T→C) positions, and in Arabidopsis, SNPs were observed at 25^th^ (G→A), 28^th^ (C→T), 30^th^ (A→T), 41^st^ (C→T), 43^rd^ (T→A) and 55^th^ (C→T) positions. Prominently, four successive SNPs were detected at the 67^th^ (G→T), 68^th^ (A→T), 69^th^ (G→T), and 70^th^ (G→A) positions in Arabidopsis (Figure 5b).

In addition to the substitutions, insertions and deletions were also observed in *MIR172D* precursor sequences. A single nucleotide insertion at the zero position was observed in *brachyantha*. An insertion of 10 nucleotides (CAAATAAACC) in sorghum and a nine-nucleotide insertion (CTTATGCCT) in Arabidopsis between the 53^rd^ and 54^th^ positions was observed. Similarly, deletion of the first 22 nucleotides (AAACAGTCGGTGCTTGCAGGTG) in sorghum, the first 19 nucleotides (AAACAGTCGGTGCTTGCAG) in maize and the first four nucleotides (AAAC) in Arabidopsis were observed. A deletion of nine nucleotides (GAGTTCATC) between the 44^th^ and 53^rd^ positions was observed in maize. Another deletion of 59 nucleotides (TGGCTGACTATATGTGATGAGAATCTTGATGATGCTGCATCAGCAAACGCTCGACTACT) from the 71^st^ to the 130^th^ position was observed in *punctata*. An eleven-nucleotide deletion (CTGACTATATG) in sorghum between the 74^th^ and 85^th^ position and an eight-nucleotide deletion (CTGACTAT) between the 74^th^ and 83^rd^ position in maize and Arabidopsis were also observed. A 19-nucleotide deletion (CAGCAAACGCTCGACTACT) from the 111^th^ to 130^th^ position in sorghum and maize was observed. A three-nucleotide deletion (ACT) at the 128^th^ to 130^th^ position was also observed in Arabidopsis (Figure 5b).

### 3.4. Spectrum of Sequence Variation in MIR172 Homologs

Tajima’s *D* 67 and Fu and Li’s *F* 68 test of neutrality of sequence polymorphisms for different *MIR172* homologs, viz., *MIR172A*, *MIR172B*, *MIR172C*, and *MIR172D* revealed non-significant negative values for all four loci. Among the four pre-*MIRNA* homologs examined across nine poaceae species, no single nucleotide polymorphism was detected in *MIR172A* and, therefore, no neutrality test was conducted for *MIR172A* (Supplementary Figures S1 and S2). The highest negative (non-significant) Tajima’s D value (−0.48816) was found in *MIR172*C, followed by *MIR172*B (−1.0403). Sequence variation for *MIR172*D was the lowest (−1.45822) and non-significant (Supplementary Figures S3, S5, and S7). Nevertheless, Fu and Li’s F values were also non-significantly negative and found to be consistent with Tajima’s D value. Similarly, *MIR172*C exhibited the highest negative Fu and Li’s value (−0.36836) followed by *MIR172*B (−0.81963), with *MIR172*D having the lowest negative value (−1.43015) (Supplementary Figures S4, S6, and S8). The nucleotide diversity of the poaceae species studied ranged from 0.05657 at the pre-*MIR172*B gene to 0.05657 at the pre-*MIR172*C locus and 0.18291 at the pre-*MIR172*D locus. These findings suggest that differences in selection pressure experienced by cultivated varieties during improvement, as well as WGD events in non-*Oryza* species, account for sequence polymorphism at distinct *MIRNA* loci.

### 3.5. Gene Conservation and Gene Density Analysis

**Figure 6:**
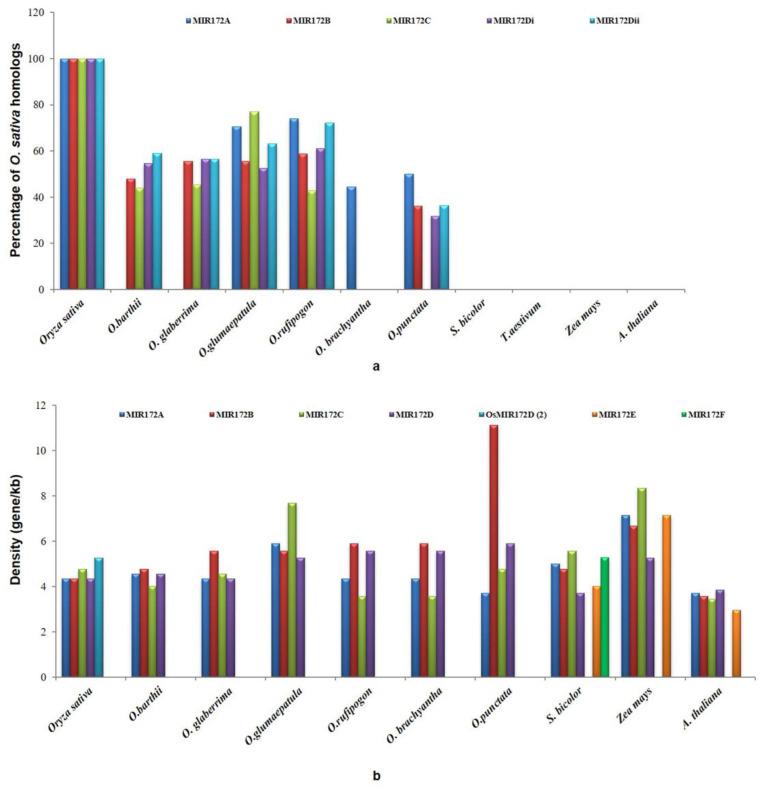
Cartoon representation of (**a**) 100 kb genomic segments containing *MIR172*A/B/C/D conserved between *O. sativa* and other poaceae members; and (**b**) the gene density in 100 kb genomic segments containing *MIR172*A/B/C/D between *O. sativa* and other poaceae members.

The number of genes predicted in the 100 kb region of each homolog of *MIR172* (Table 2) revealed the presence of 23 genes around the 100 kb region *MIR172A* of *Oryza sativa,* and the highest percentage of *sativa* homologs were found to be conserved in *rufipogon* (73.91%, i.e., 17 conserved genes out of 23 detected genes). The lowest conservation (0%) was found in *barthii, glaberrima*, sorghum, maize, and Arabidopsis, i.e., disruptive synteny with complete loss of conservation. Similarly, the maximum gene density was found in *Oryza punctata* (1gene/3.703 kb), and the minimum density of genes was observed in maize (1gene/7.14 kb) (Figure 6a,b). We observed the presence of 23 genes in the 100 kb region of *MIR172B* of *sativa,* with 58.8% conservation with *rufipogon* (10 out of 17 detected genes), and the lowest conservation of 0% was detected in *brachyantha*, sorghum, and maize (disruptive synteny), including Arabidopsis. The maximum gene density was found in sorghum (1gene/3.073 kb), and the minimum density of genes was observed in *punctata* (1gene/5.88 kb) (Figure 6a,b).

In *MIR172C,* 21 genes were identified in the 100 kb region of *Oryza sativa* with 76.9% conservation in *glumaepatula* (10 out of 13 genes) and the lowest in *punctata*, *brachyantha,* sorghum, and maize (0%, i.e., disruptive synteny), including Arabidopsis. At the same time, maximum gene density was found in *rufipogon* and *brachyantha* (1gene/3.571 kb); the minimum density of genes was observed in maize (1gene/8.33 kb) (Figure 6a,b). Two homeologs of *MIR172D,* i.e., *MIR172D*i and *MIR172D*ii, were identified in the *sativa* genome, and 23 genes were identified in the 100 kb region harboring *MIR172D*i of *sativa.* The highest percentage of *sativa* homologs were found to be conserved in *rufipogon* (61.1%, i.e., 11 out of 18 genes) and the lowest in *brachyantha*, sorghum, Arabidopsis, and maize (0%, i.e., disruptive synteny (Figure 6a). It is observed that the maximum gene density is 1 gene/3.703 kb in sorghum, while the minimum gene density of 1 gene/5.882kb was observed in *punctata* (Figure 6b).

Furthermore, 19 genes were identified in the 100 kb region harboring *MIR172D*ii of *Oryza sativa* sub-species *indica,* and the highest percentage of *sativa* homologs were found to be conserved in *rufipogon* (72.2%, i.e., 13 out of 18 genes). However, the disruptive synteny showing a total loss of conservation was detected in *brachyantha*, sorghum, and maize. Further, the synteny results revealed the highest gene density of 1 gene/3.073 kb in sorghum, with *punctata* exhibiting the lowest gene density of 1 gene/5.88 kb.

### 3.6. Microsynteny Analysis

Inter-species synteny block analysis of *MIRNA172* homologs revealed that out of 218 genes in *MIR172A*, 129 genes (59.17%) were collinear. Similarly, 88 genes (45.13%) out of 195 predicted genes in *MIR172B*; 77 genes (36.49%) out of predicted 211 genes in *MIR172C*; and 91 genes (38.72%) out of 235 predicted genes for *MIR172D* were collinear. The demonstration of the alignment of non-anchor genes is marked by ‘||’ in the multi-alignment of gene orders. Synteny or collinearity blocks were (Supplementary Figures S9–S45) constructed using *sativa* and other relevant reference genomes to develop saturated synteny/collinearity blocks for each homolog of *MIRNA*.

(i) MIR172A

**Figure 7:**
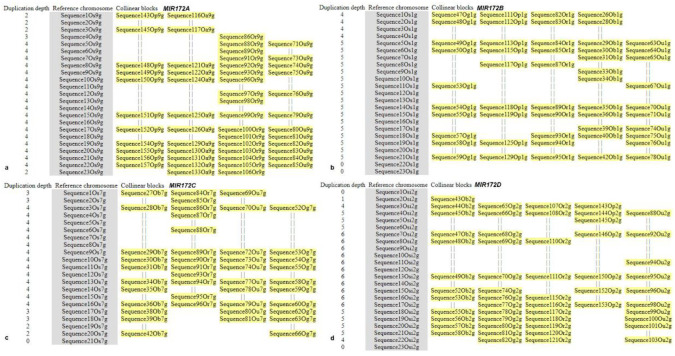
Evaluation of 100 kb regions of *MIR172* amongst poaceae family. Collinearity blocks of (**a**) *MIR172*A, (**b**) *MIR172*B, (**c**) *MIR172*C, and (**d**) *MIR172*D with *Oryza sativa* as reference. The first column indicates the depth of duplication at each gene locus; the second column indicates the genes in reference chromosomes, and the subsequent columns represent aligned collinear blocks with the matched genes. Alignment among non-anchor genes is removed in the output and represented by ‘||’ in the multi-alignment of gene ordering.

**Figure 8:**
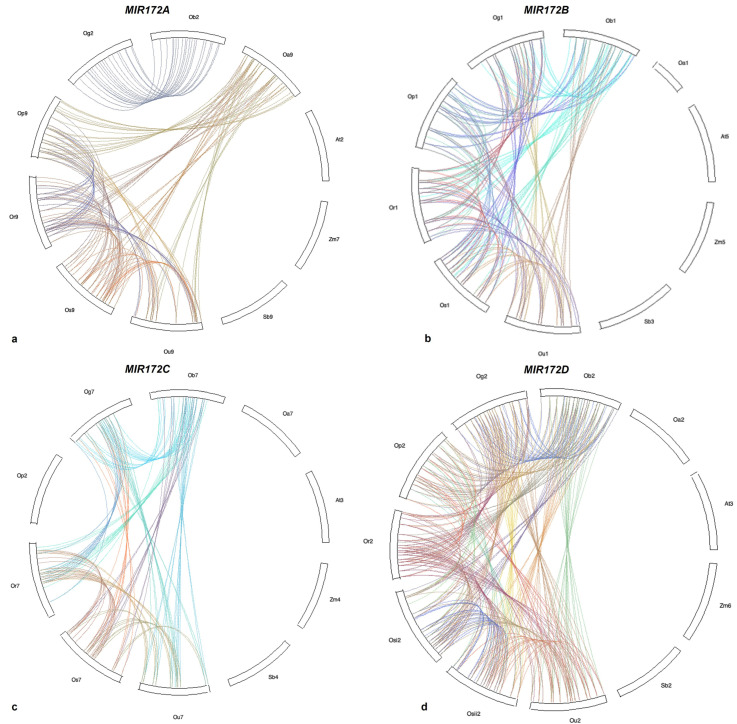
Analysis of 100 kb region flanking *MIR172* amongst Poaceae members. (**a**) Circular plot of *MIR172*A displaying synteny and collinearity patterns. Os9-*Oryza sativa* chr 9, og2-*Oryza glaberrima* chr 2, ob2-*Oryza barthii* chr 2, ou9-*Oryza glumaepatula* chr 9, oa9-*Oryza brachyantha* chr 9, or9-*Oryza rufipogon* chr 9, op9-*Oryza punctata* chr 9, zm7-*Zea mays* chr 7, sb9-*Sorghum bicolor* chr 9, at2-*Arabidopsis thaliana* chr 2; (**b**) Circular plot displaying synteny and collinearity patterns in *MIR172*B. os1-*Oryza sativa* chr 1, og1-*Oryza glaberrima* chr 1, ob1-*Oryza barthii* chr 1, ou1-*Oryza glumaepatula* chr 1, oa1-*Oryza brachyantha* chr 1, or1-*Oryza rufipogon* chr 1, op1-*Oryza punctata* chr 1, zm5-*Zea mays* chr 5, sb3-*Sorghum bicolor* chr 3, at5-*Arabidopsis thaliana* chr 5; (**c**) Circular plot displaying synteny and collinearity patterns in *MIR172*C. os7-*Oryza sativa* chr 7, og7-*Oryza glaberrima* chr 7, ob7-*Oryza barthii* chr 7, or7-*Oryza rufipogon* chr 7, ou7-*Oryza glumaepatula* chr 7, op2*-Oryza punctata* chr 2, oa7-*Oryza brachyantha* chr 7, zm4-*Zea mays* chr 4, sb4-*Sorghum bicolor* chr 4, at3-*Arabidopsis thaliana* chr 3; and (**d**) Circular plot displaying synteny and collinearity patterns in *MIR172*D. osi2-*Oryza sativa* homeolog i chr 2, osii2-*Oryza sativa* homeolog ii chr 2, og2-*Oryza glaberrima* chr 2, ob2-*Oryza barthii* chr 2, ou2-*Oryza glumaepatula* chr 2, oa2-*Oryza brachyantha* chr 2, or2-*Oryza rufipogon* chr 2, op2-*Oryza punctata* chr 2, zm6-*Zea mays* chr 6, sb2-*Sorghum bicolor* chr 2, at3-*Arabidopsis thaliana* chr 3.

Although conservation of microsynteny in the 100 kb region surrounding *MIR172A* was detected amongst *brachyantha*, *glumaepetula*, *rufipogon,* and *punctata*; a complete loss of microsynteny was observed within *barthii* and *glaberima,* along with sorghum, maize, and Arabidopsis. An in-depth analysis of the 100 kb region in *MIR172A* of *sativa* revealed the presence of 23 genes, of which seven genes viz., *Chitin elicitor receptor kinase* 1-like, *P-loop NTPase* domain-containing protein LPA1 homolog, *Glucomannan 4-beta-mannosyltransferase* 1-like, probable *serine/threonine-protein kinase* PBL23, pollen-specific protein C13-like, dynamin-related protein 1E-like, and pentatricopeptide repeat-containing protein *At5g66520*-like were found to be conserved in *brachyantha*, *glumaepetula*, *rufipogon*, and *punctata*. Genes such as *Retrotransposon protein*, putative, unclassified, and *Ethylene responsive transcription factor 1B* of *sativa* were also found to be conserved in *glumaepatula* and *rufipogon*. Further analysis revealed that *diacylglycerol lipase-beta* and *Ethylene-responsive transcription factor ERF096* of *sativa* were conserved in *brachyantha* and *punctata*. Thus, we conclude that the overall microsynteny in *MIR172A* was gradually lost from *sativa* to its wild relatives and further to dicot species (Figure 7a and Figure 8a).

(ii) MIR172B

Similarly, conservation of microsynteny in a 100 kb region surrounding *MIR172B* was detected only in *Oryza punctata*, *glumaepatula*, *rufipogon*, *barthii*, and *glaberima* (Figure 5b and Figure 6b) with the exception of *Oryza brachyantha* that exhibited complete loss of microsynteny. In the 100 kb region, altogether, 23 genes were detected in *Oryza sativa*, of which *carboxyl-terminal peptidase-like*, *pumilio homolog 1-like*, *protein FIZZY-RELATED 3*, putative *WRKY transcription factor 49*, uncharacterized protein LOC4326395, *zinc transporter 1*, and probable *E3 ubiquitin-protein ligase RHC2A* genes were found to be conserved in all five *Oryza* spp. LUX gene was found to be conserved in *punctata*, *rufipogon*, *barthii,* and *glaberima*. *Oryza* homolog *Retrotransposon protein*, and Ty3-gypsy subclass were conserved in *glumaepatula,* and *Phosphatidylinositol/phosphatidylcholine transfer protein SFH13* was conserved in *barthii*. However, none of the *sativa* homologs were detected in sorghum/maize/Arabidopsis (Figure 7b and Figure 8b), showing a complete loss of microsynteny.

(iii) MIR172C

Analysis in a 100 kb region surrounding *MIR172*C revealed the conservation of microsynteny amongst *glumaepatula*, *rufipogon*, *barthii,* and *glaberima* (Figure 7c and Figure 8c). However, a complete loss of microsynteny was observed in *punctata* and *brachyantha*. Amongst 21 genes identified in the 100 kb region harboring *MIR172C* of *sativa*, six genes, namely, *F-actin-capping protein subunit alpha*, *Ribosomal protein L23/L15e family protein*, hypothetical *protein OsI_25745*, *adenyl cyclase-like protein*, *G-type lectin S-receptor-like serine/threonine-protein kinase At1g34300* and *Retrotransposon protein*, *Ty1-copia subclass* were found to be conserved in *glumaepatula*, *rufipogon*, *barthii,* and *glaberima.* Another hypothetical protein gene *OsI_25741* was found to be conserved in *barthii*, *rufipogon*, and *glumaepatula*. Similarly, hypothetical *protein DAI22_07g114300* and *fatty acyl-CoA reductase1* were found to be conserved in *barthii*, *glumaepatula*, and *glaberrima*. Genes such as *retrotransposon protein*, *Ty3-gypsy subclass* was found to be conserved in *barthii* and *glaberrima*. Amongst others, *activator-like transposable element* and *phosphatidylinositol 4-phosphate 5-kinase 6-like* are the two genes found conserved only in *rufipogon* while no *sativa* homologs were conserved in sorghum/maize/Arabidopsis indicating complete disruption of microsynteny (Figure 7c and Figure 8c).

(iv) MIR172D

Microsynteny conservation in the 100 kb region surrounding *MIR172D* was detected amongst *punctata*, *glumaepatula*, *rufipogon*, *barthii*, and *glaberima,* but the same was completely lost in *brachyantha*. Two homeologs of *MIR172D,* i.e., Os*MIR172Di* and Os*MIR172Dii,* were identified in the *sativa* genome. Out of the 23 genes detected in *OsMIR172Di* in the 100 kb region harboring *MIR172D* of *sativa*, two genes, namely protein *LUTEIN DEFICIENT 5* and *pentatricopeptide repeat-containing protein At2g22410* were found to be conserved in *punctata*, *glumaepatula*, *rufipogon*, *barthii,* and *glaberima*. The genes *heavy metal-associated isoprenylated plant protein 3-like* and *Pib variant* protein were found to be conserved in *punctata*, *glumaepatula*, *barthii,* and *glaberima*. Another rice homolog, putative *brown planthopper-induced resistance protein 1* was conserved in *punctata*, *rufipogon*, *barthii, glaberima,* and hypothetical *protein DAI22_02g385300* was conserved in *punctata*, *glumaepatula*, *rufipogon,* and *glaberima*. Further, genes such as *DNA ligase-like* and probable protein *S-acyltransferase 15* were conserved in all *Oryza* spp except *punctata*, *glumaepatula*, and *brachyantha*. Other genes, such as putative *telomere binding protein-1; TBP1*, *cyst nematode resistance protein-like* protein, and protein *N-lysine methyltransferase METTL21A* were observed to be conserved in *barthii*, *punctata,* and *glumaepatula,* respectively. No *sativa* homologs were found to be conserved in sorghum, maize, and Arabidopsis, indicating complete disruption of microsynteny (Figure 7d and Figure 8d).

Out of the 19 genes identified in the 100 kb region of OsMIR172Dii detected in sativa, four genes, pentatricopeptide repeat-containing protein At2g22410, heavy metal-associated isoprenylated plant protein 3-like, thioredoxin-like 3–2 and OTU domain-containing protein 5-A were found to be conserved in punctata, glumaepatula, rufipogon, barthii, and glaberima. Rice homolog, hypothetical protein DAI22_02g385300 was conserved in punctata, glumaepatula, and rufipogon; pumilio homolog 1-like was conserved in punctata, glumaepatula, rufipogon, barthii, and glaberima; and P-loop NTPase domain-containing protein LPA1 was conserved in glumaepatula, rufipogon, barthii, and glaberima. Further, genes named DNA ligase-like and probable protein S-acyltransferase 15 were found to be conserved in rufipogon, barthii, and glaberima, while the Pib variant protein was conserved in punctata, glaberrima, and barthii. Nonetheless, no sativa homologs were detected in sorghum, maize, or Arabidopsis, indicating complete disruption of microsynteny.

### 3.7. Phylogenomic Analysis

**Figure 9:**
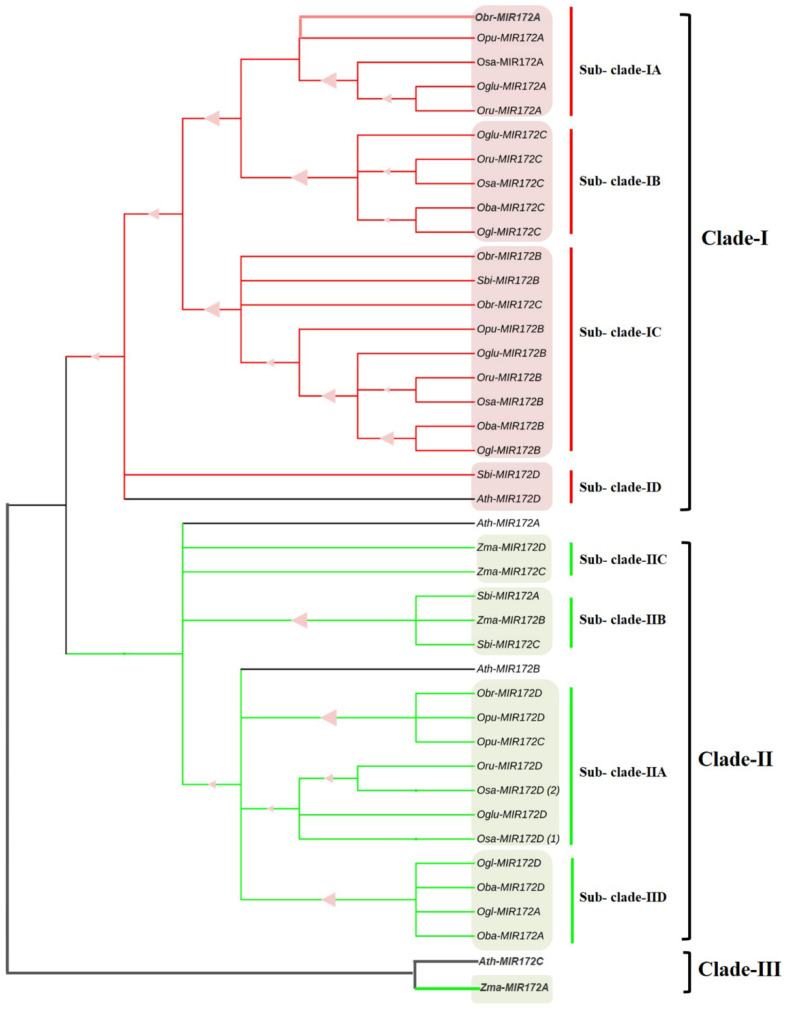
Phylogenetic analysis of *MIR172*. The ML phylogenetic tree shows 40 *MIR172* precursors and promoter sequences (500 bp) from *Oryza sativa* and its six wild cousins, *Sorghum bicolor* and *Zea mays*. Arabidopsis was included as an outlier. Bootstrap values (0.53 to 1.000) are represented by triangles. Maximum-likelihood tree revealed that *MIR172* sequences clustered into three clades viz. Clade-I, Clade-II, and Clade-III. Osa-*Oryza sativa*, Ogl-*Oryza glaberrima*, Oba-*Oryza barthii*, Oru-*Oryza rufipogon*, Oglu-*Oryza glumaepatula*, Opu-*Oryza punctata*, Obr-*Oryza brachyantha*, Zma-*Zea mays*, Sbi-*Sorghum bicolor*, Ath-*Arabidopsis thaliana*.

Phylogenomic analysis of *MIR172* homologs revealed that they had undergone whole genome duplications and polyploidization events to segregate into different clades. Maximum-likelihood tree revealed that *MIR172* sequences clustered into three clades viz. Clade-I, Clade-II, and Clade-III (Figure 9). Clade-I included *sativa*, *brachyantha*, *glaberima*, *rufipogon,* and *punctata MIR172A* that formed Sub-clade-IA, while, *sativa*, *barthii*, *glaberima*, *glumaepetula*, and *rufipogon MIR172C* formed Sub-clade-IB. Similarly, *sativa*, *barthii*, *brachyantha*, *glaberima*, *glumaepetula*, *rufipogon*, *punctata*, and sorghum *MIR172B* along with *brachyantha MIR172C* formed Sub-clade-IC; sorghum along with Arabidopsis *MIR172D* formed Sub-clade ID. Within Clade-II, *sativa* (1 and 2), *barthii*, *brachyantha*, *glaberima*, *glumaepetula*, *rufipogon*, and *punctata MIR172D* along with *punctata MIR172C* formed Sub-clade IIA. Sorghum *MIR172A* and *C* and maize *MIR172B* formed Sub-clade-IIB; maize *MIR172C* and *D* formed Sub-clade-IIC; *barthii* and *glaberima MIR172A* formed Sub-clade-II-D. Maize *MIR172A,* along with Arabidopsis *MIR172C* formed Clade-III (Figure 9). Maize formed a separate sub-clade IIC or was grouped with Arabidopsis in clade III. This indicates that *MIR172* (A–D) share a common evolutionary descendent.

## 4. Discussion

Studies have shown that whole-genome duplication (WGD), in addition to the tandem duplication of *MIRNA* genes, are involved in their evolution, but its contribution varies from species to species [69], which means apart from their ancestral origin, plant *MIRNA*s may have been generated by duplication of pre-existing *MIRNA* genes Our study revealed the presence of single nucleotide polymorphism in precursor sequences of *MIR172* homologs at multiple positions along with deletions and insertions (Figure 3, Figure 4 and Figure 5). This can be ascribed to two rounds of WGD that Arabidopsis underwent after splitting from papaya during the course of evolution [70]. However, monocots, unlike eudicots such as Arabidopsis, underwent only one shared ancestral WGD during their evolution [70]. Our analysis of the precursor sequences of *MIR172A* (Figure 4) in poaceae revealed that it is conserved in *Oryza* except in *barthii*, *glaberrima*, *punctata*, and *rufipogon,* which showed deletion in a few positions. Nonetheless, *brachyantha* and *punctata* showed deletion in *MIR172B* and *MIR172C,* while an insertion in *brachyantha* and a deletion in *punctata* was observed in *MIR172D* (Figure 5). These can be ascribed to the genome downsizing and resistance to genome expansion in *brachyantha* and *glaberrima* that could have led to structural variation. Similarly, in *punctata* and *barthii* both genome expansion and contraction might have led to structural variations [71]. The polymorphism in the precursor sequences of sorghum and maize could be ascribed to the WGD event that occurred 30 million years ago (MYA), separating the lineage of sorghum and maize from that of rice [72] from its common ancestors. Further, the variations in the precursor sequence of maize could be due to an extra WGD event leading to the expansion of *MIRNA* gene family [73]. In fact, duplicated *MIRNA* genes in maize underwent extensive gene loss, with approximately 35% of ancestral sites retained as duplicate homoeologous *MIRNA* genes [74]. Common grass triplication and genome hybridization in wheat [75] might be accountable for the complete deletion of wheat *MIR172* homologs. WGDs shape up the number of *MIRNA* genes, but their number and pattern vary in a species-specific manner [69]. This might be responsible for the disruption of microsynteny among rice wild relatives and the complete loss of microsynteny in sorghum, maize, and Arabidopsis.

*MIR172* belongs to highly conserved *MIRNA* families [22], and the multiple sequence alignment of mature *MIR172* revealed that it is conserved amongst all the seven *Oryza* species. Our result is commensurate with a previous report that *MIRNA* genes contain fewer SNPs than their contiguous border region, and the mature sequence of the *MIRNA* genes contains fewer SNPs than their precursors [76,77]. The results of multiple sequence alignment of mature and precursor MIR172 revealed the same as we observed fewer SNPs in mature *MIRNA* sequence in comparison to precursor sequences. SNPs have a different impact on the functionality of *MIRNA;* for example, variants in miRNA promoter regions and other regulatory regions may result in an altered transcription rate, variants in splice sites of the host gene (for intronic miRNAs) or of the poly-cistron (clustered miRNAs) can result in aberrant expression patterns. In this regard, Tajima’s D [67] and Fu and Li’s F [68] tests were used to estimate the neutrality of sequence polymorphism for *MIRNA* genes. These tests detect both positive and balancing selections [78]. Our results suggest non-neutral sequence variations in all the *MIRNA*s. Tajima’s D (−1.45822) and Fu and Li’s F (−1.43015) values for the *MIR172D* locus were negative, while both test values were least negative at the *MIR172*C locus [Tajima’s D (−0.48816); Fu and Li’s F (−0.36836)]. Previously, negative findings in one or both tests were reported in rice and Arabidopsis [5,68]. Similarly, the nucleotide diversity was found to differ amongst *MIRNA* locations. As a result, *MIRNA*s are effective targets for the differential accumulation of variation in populations subjected to selection pressures.

Expression analysis of the mature *MIR172* sequences in root and shoot tissues at different developmental stages helped in determining the spatial and temporal expression of each *MIR172* member. However, discerning expression of individual *MIR172* family members was unlikely as two out of four *MIRNA* homologs (*MIR172A* and *D*) are completely conserved, while the other two *MIRs* (*MIR172B* and *C*) have minimal sequence differences. Comparative analysis of *MIR172* showed similar expression profiles in different developmental tissues (Figure 2). Insight into *MIR172* expression revealed that it is highly expressed in late vegetative stages (10 leaf-stage-12.23-fold), flag leaf (25.97-fold), and developing panicles (13.465-fold). However, there was no increase in its expression in roots in rice. A similar trend of expression was observed in other poaceae members and Arabidopsis as well. Lower levels of *MIR172* expression observed in the early vegetative stage can be ascribed to its role in the transition [79] of plant development from juvenile to the flowering stage by regulating AP2-like genes, including the *target of early activation tagged 1/2/3*. Higher expression of *MIR172* in late vegetative and panicle (Figure 2) is consistent with its role in the acquisition of floral competence [80]. The increasing level of *MIR172* is commensurate with the appearance of adult traits, as its over-expression leads to early flowering (Figure 2).

Microsynteny/collinearity analysis provides an insight into the shared ancestry of groups of genes and unravels the evolutionary history of genomes and gene families, establishing gene orthology [81]. Microsynteny analysis of *MIR172* revealed that gene collinearity within the genus *Oryza* is conserved. However, disruption of synteny in the 100 kb region of *MIR172A* in *barthii* (AA) and *glaberima* (AA), of *MIR172B* in *brachyantha* (FF), and of *MIR172C* in *punctata* (BB) was observed. *Oryza spp*. That lost microsynteny belonged to three distinct sub-genomes. Amongst these, *glaberrima* is a domesticated species, while the other three are wild relatives. Evolutionary studies revealed that the AA genome diverged from FF progenitors by almost ~15 MYA [82], whereas the divergence between AA- and BB- genomes occurred at 9.11 MYA [83]. Gene loss events observed in *glaberrima* (AA)*, barthii* (AA), and *brachyantha* (FF) plausibly led to the differences in gene family content.

The progenitors of *brachyantha* and *punctata* diverged from *sativa* progenitors during the course of evolution from FF- and BB- genomes to AA-*Oryza* genomes, in spite of a well-conserved genome organization and well-preserved gene order. Loss of microsynteny in *MIR172*A in *barthii* (AA) and *glaberima* (AA), *MIR172B* in *brachyantha* (FF), and *MIR172C* in *punctata* (BB) can be due to these factors. It is reported that *sativa* was domesticated from *rufipogon* (perennial wild rice) around 9000 years ago in Asia, and *glaberrima* was domesticated from *barthii* independently, around 3000 years ago in West Africa [84,85]. This corroborates our result as the highest number of *sativa* gene homologs were found to be conserved in *rufipogon* for *MIR172A*, *B* and *D* followed by *MIR172C* (Figure 7). Additionally, most of the *barthii* gene homologs were conserved in *glaberrima* (Supplementary Figures S1, S2, S10, S11, S19, S20, S28, and S29).

## 5. Conclusions

Our in-depth analysis of microsynteny/collinearity of *MIR172* and its homologs in seven different *Oryza* species, sorghum, maize, and Arabidopsis is a comprehensive evolutionary study based on *MIRNA* sequence variation and conservation of orthologous genes. We identified the orthologous *MIRNA* genes of rice and, microsynteny analysis, genes harbored around 100 kb region, revealed that the gene pattern and content are conserved (conservative evolution) among *Oryza* species with exceptions depending upon the genome type and selection pressure during evolution/domestication. However, the microsynteny in sorghum, maize, and Arabidopsis was completely lost (disruptive evolution) during the course of evolution due to WGD events. Gain/loss of genes or chromosomal repatterning might have caused structural variation, but overall gene content and order in *MIRs* are maintained. Low genetic diversity at different *MIRNA* loci in cultivated rice revealed that the rice wild relatives are the untapped genetic reservoirs to be harnessed for crop improvement. Abiotic [86] and biotic stresses [43] due to inconsistent climate indices and population outbursts pose a threat to national food security. The green revolution has no doubt provided superior cultivars for improved food grain production, but stringent selection has created a bottleneck in the genetic variability in domesticated crops. Owing to their habitat in a robust natural environment, crop wild relatives managed to maintain a higher level of genetic variability. Utilizing the untapped genetic resources available in CWRs for crop improvement is an attractive option for further improving food production in the future.

## Figures and Tables

**Table 1:** Chromosomal locations and coordinates of each *MIR172* homologs in selected grasses. *MIR172* homolog were identified in all seven rice species, sorghum, maize, and Arabidopsis, except wheat.

Species	*MIRNA*	Location	Precursor Coordinates	
			Start	End
*Oryza sativa*	*MIR172* *A*	Chr 9	21,688,003	21,688,111
	*MIR172* *B*	Chr 1	46,953,572	46,953,809
	*MIR172* *C*	Chr 7	12,288,035	12,288,145
	*MIR172* *D*	Chr 2	37,298,091	37,298,220
		Chr 2	37,328,665	37,328,794
*Oryza barthii*	*MIR172* *A*	Chr 2	30,983,600	30,983,623
	*MIR172* *B*	Chr 1	36,558,471	36,558,708
	*MIR172* *C*	Chr 7	10,711,579	10,711,689
	*MIR172* *D*	Chr 2	30,983,582	30,983,711
*Oryza glaberrima*	*MIR172* *A*	Chr 2	28,473,275	28,473,298
	*MIR172* *B*	Chr 1	32,331,490	32,331,727
	*MIR172* *C*	Chr 7	10,632,540	10,632,650
	*MIR172* *D*	Chr 2	28,473,257	28,473,386
*Oryza glumaepatula*	*MIR172* *A*	Chr 9	23,407,188	23,407,296
	*MIR172* *B*	Chr 1	46,207,906	46,208,139
	*MIR172* *C*	Chr 7	12,334,350	12,334,460
	*MIR172* *D*	Chr 2	37,285,412	37,285,541
*Oryza punctata*	*MIR172* *A*	Ch9	26,230,640	26,230,686
	*MIR172* *B*	Chr1	45,791,747	45,791,791
	*MIR172* *C*	Chr2	38,821,836	38,821,861
	*MIR172* *D*	Chr2	38,821,883	38,821,953
*Oryza rufipogon*	*MIR172* *A*	Chr 9	20,417,006	20,417,114
	*MIR172* *B*	Chr 1	39,578,018	39,578,255
	*MIR172* *C*	Chr 7	11,443,165	11,443,275
	*MIR172* *D*	Chr 2	33,182,612	33,182,741
*Oryza brachyantha*	*MIR172* *A*	Chr9	14,032,760	14,032,800
	*MIR172* *B*	Chr1	33638435	33638477
	*MIR172* *C*	Chr7	7,379,288	7,379,325
	*MIR172* *D*	Chr2	26,379,138	26,429,195
*Sorghum bicolor*	*MIR172* *A*	Chr 9	58,774,558	58,774,659
	*MIR172* *B*	Chr 3	74,188,339	74,188,508
	*MIR172* *C*	Chr 4	67,645,991	67,646,109
	*MIR172* *D*	Chr 2	22,201,215	22,201,302
	*MIR172*E	Chr 2	14,122,957	14,123,071
	*MIR172*F	Chr 5	19,295,951	19,296,068
*Zea mays*	*MIR172* *A*	Chr 7	54,276,707	54,276,789
	*MIR172* *B*	Chr 5	221,721,349	221,721,474
	*MIR172* *C*	Chr 4	174,154,928	174,155,050
	*MIR172* *D*	Chr 6	172,029,786	172,029,859
	*MIR172*E	Chr 3	145,801,592	145,801,765
*Arabidopsis thaliana*	*MIR172* *A*	Chr2	11,942,914	11,943,015
	*MIR172* *B*	Chr5	1,188,207	1,188,301
	*MIR172* *C*	Chr3	3,599,776	3,599,908
	*MIR172* *D*	Chr3	20,587,904	20,588,027
	*MIR172*E	Chr5	23,988,472	23,988,596

**Table 2:** Prediction of gene number and homologs of *MIR172* in respective grasses with Arabidopsis as outliers. Notably, four homologs in *Oryza*, five in maize and Arabidopsis, and six in sorghum were identified with gene content as low as 1–2 in *O. sativa* (*MIR172*D) and as high as 25–27 in *S. bicolor* (*MIR172*D).

	*MIRNA*		Plant Species										
			*Oryza sativa*	*Oryza barthii*	*Oryza glaberrima*	*Oryza glumaepatula*	*Oryza rufipogon*	*Oryza brachyantha*	*Oryza punctata*	*Sorghum bicolor*	*Triticum aestivum*	*Zea mays*	*Arabidopsis thaliana*
No. of genes predicted	*MIR172*	*MIR172*A	23	22	23	17	23	27	22	20	0	14	27
		*MIR172*B	23	21	18	18	17	9	25	21	0	15	28
		*MIR172*C	21	25	22	13	28	21	22	18	0	12	29
		*MIR172*D	1–23, 2–19	22	23	19	18	17	22	27	0	19	26
		*MIR172*E	0	0	0	0	0	0	0	25	0	14	34
		*MIR172*F	0	0	0	0	0	0	0	19	0	0	0
No. of homologs predicted		*MIR172*A	1	1	1	1	1	1	1	1	0	1	1
		*MIR172*B	1	1	1	1	1	1	1	1	0	1	1
		*MIR172*C	1	1	1	1	1	1	1	1	0	1	1
		*MIR172*D	2	1	1	1	1	1	1	1	0	1	1
		*MIR172*E	0	0	0	0	0	0	0	1	0	1	1
		*MIR172*F	0	0	0	0	0	0	0	1	0	0	0

## Data Availability

Not applicable.

## Supplementary Materials

The following supporting information can be downloaded at: https://www.mdpi.com/article/10.3390/cells12101370/s1, Dash et al. supplementary file S1.pdf, Figure S1: Screenshot of Tajima’s test conducted on *MIR172A* sequences; Figure S2: Screenshot of Fu and Li’s test conducted on *MIR172A* sequences; Figure S3: Screenshot of Tajima’s test conducted on *MIR172B* sequences; Figure S4: Screenshot of Fu and Li’s test conducted on *MIR172B* sequences; Figure S5: Screenshot of Tajima’s test conducted on *MIR172C* sequences; Figure S6: Screenshot of Fu and Li’s test conducted on *MIR172C* sequences; Figure S7: Screenshot of Tajima’s test conducted on *MIR172D* sequences; Figure S8: Screenshot of Fu and Li’s test conducted on *MIR172D* sequences; Figure S9: Synteny block diagram for *MIR172A* with *Oryza barthii* as reference; Figure S10: Synteny block diagram for *MIR172A* with *Oryza glaberima* as reference; Figure S11: Synteny block diagram for *MIR172A* with *Oryza glumaepetula* as reference; Figure S12: Synteny block diagram for *MIR172A* with *Oryza rufipogon* as reference; Figure S13: Synteny block diagram for *MIR172A* with *Oryza brachyantha* as reference; Figure S14: Synteny block diagram for *MIR172A* with *Oryza punctata* as reference; Figure S15: Synteny block diagram for *MIR172A* with *Sorghum bicolor* as reference; Figure S16: Synteny block diagram for *MIR172A* with *Zea mays* as reference; Figure S17: Synteny block diagram for *MIR172A* with *Arabidopsis thaliana* as reference; Figure S18: Synteny block diagram for *MIR172B* with *Oryza barthii* as reference; Figure S19: Synteny block diagram for *MIR172B* with *Oryza glaberima* as reference; Figure S20: Synteny block diagram for *MIR172B* with *Oryza glumaepetula* as reference; Figure S21: Synteny block diagram for *MIR172B* with *Oryza rufipogon* as reference; Figure S22: Synteny block diagram for *MIR172B* with *Oryza brachyantha* as reference; Figure S23: Synteny block diagram for *MIR172B* with *Oryza punctata* as reference; Figure S24: Synteny block diagram for *MIR172B* with *Sorghum bicolor* as reference; Figure S25: Synteny block diagram for *MIR172B* with *Zea mays* as reference; Figure S26: Synteny block diagram for *MIR172B* with *Arabidopsis thaliana* as reference; Figure S27: Synteny block diagram for *MIR172C* with *Oryza barthii* as reference; Figure S28: Synteny block diagram for *MIR172C* with *Oryza glaberima* as reference; Figure S29: Synteny block diagram for *MIR172C* with *Oryza glumaepetula* as reference; Figure S30: Synteny block diagram for *MIR172C* with *Oryza rufipogon* as reference; Figure S31: Synteny block diagram for *MIR172C* with *Oryza brachyantha* as reference; Figure S32: Synteny block diagram for *MIR172C* with *Oryza punctata* as reference; Figure S33: Synteny block diagram for *MIR172C* with *Sorghum bicolor* as reference; Figure S34: Synteny block diagram for *MIR172C* with *Zea mays* as reference; Figure S35: Synteny block diagram for *MIR172C* with *Arabidopsis thaliana* as reference; Figure S36: Synteny block diagram for *MIR172D* with *Oryza barthii* as reference; Figure S37: Synteny block diagram for *MIR172D* with *Oryza glaberima* as reference; Figure S38: Synteny block diagram for *MIR172D* with *Oryza glumaepetula* as reference; Figure S39: Synteny block diagram for *MIR172D* with *Oryza rufipogon* as reference; Figure S40: Synteny block diagram for *MIR172D* with *Oryza brachyantha* as reference; Figure S41: Synteny block diagram for *MIR172D* with *Oryza punctata* as reference; Figure S42: Synteny block diagram for *MIR172D* with *Oryza sativa* (ii) as reference; Figure S43: Synteny block diagram for *MIR172D* with *Sorghum bicolor* as reference; Figure S44: Synteny block diagram for *MIR172D* with *Zea mays* as reference; Figure S45: Synteny block diagram for *MIR172D* with *Arabidopsis thaliana* as reference; Table S1: Coordinates for 100 kb region harboring *MIRNA*s for microsynteny analysis and coordinates for promoter and precursors for phylogenetic analysis; Table S2: Forward and reverse primer sequence for amplification of *MIR172A, B, C* and *D* from different poaceae members and Arabidopsis: Table S3: Forward primer sequence for RT-qPCR used for generating the expression profile of *MIR172* in different members of poaceae and Arabidopsis; Supplementary Data S1: Nucleotide sequence (Sanger sequence) of PCR amplified *MIR172 A-D* from different poaceae members; (2) Dash et al. supplementary file S2.xls.
