# Supplementary material for: Phylogenomic Analysis of micro-RNA Involved in Juvenile to Flowering-Stage Transition in Photophilic Rice and Its Sister Species
Source: Cells. 2023 May 12;12(10):1370. doi: 10.3390/cells12101370 (PMC10216681; doi:10.3390/cells12101370)
Supplement: Supplementary file 1 [file cells-12-01370-s001.zip › supplementary File.pdf]

cells

MDPI

Table S1: Coordinates for 100kb region harboring *MIR172* for microsynteny analysis and coordinates for promoter and precursor for phylogenetic analysis.

Species	miRNA	Chromosome location	100 Kb region coordinates		coordinates for phylogenetic analysis	
			Start position	End position	Start position	End position
Oryza sativa	miR172A	Chr 9	21638003	21738111	21687503	21688111
	miR172B	Chr 1	46903572	47003809	46953072	46953809
	miR172C	Chr 7	12238035	12338145	12287535	12288145
	miR172D	Chr 2	37248091	37348220	37297591	37298220
		Chr 2	37278665	37378794	37328165	37328794
Oryza barthii	miR172A	Chr 2	30933600	31033623	30983100	30983623
	miR172B	Chr 1	36508471	36608708	36557971	36558708
	miR172C	Chr 7	10661579	10761689	10711079	10711689
	miR172D	Chr 2	30933582	31033711	30983082	30983711
Oryza glaberrima	miR172A	Chr 2	28423276	28523296	28472775	28473298
	miR172B	Chr 1	32281490	32381727	32330990	32331727
	miR172C	Chr 7	10582540	10682650	10632040	10632650
	miR172D	Chr 2	28423257	28523386	28472757	28473386
Oryza glumaepatula	miR172A	Chr 9	23357188	23457296	23406688	23407296
	miR172B	Chr 1	46157906	46258139	46207406	46208139
	miR172C	Chr 7	12284350	12384460	12333850	12334460
	miR172D	Chr 2	37235412	37335541	37284912	37285541
Oryza punctata	miR172A	Ch9	26180640	26280686	26230140	26230686
	miR172B	Chr1	45741747	45841791	45791247	45791791
	miR172C	Chr2	38771836	38871861	38821336	38821861
	miR172D	Chr2	38771883	38871953	38821383	38821953
Oryza rufipogon	miR172A	Chr 9	20367006	20467114	20416506	20417114
	miR172B	Chr 1	39528018	39628255	39577518	39578255
	miR172C	Chr 7	11393165	11493275	11442665	11443275
	miR172D	Chr 2	33132612	33232741	33182112	33182741
Oryza brachyantha	miR172A	Chr9	13982760	14082800	14032260	14032800
	miR172B	Chr1	33588435	33688477	33587935	33638477
	miR172C	Chr7	7329288	7429325	7378788	7379325
	miR172D	Chr2	26329138	26429195	26378638	26429195
Sorghum bicolor	miR172A	Chr 9	58724558	58824659	58774058	58774659
	miR172B	Chr 3	74138339	74238508	74187839	74188508
	miR172C	Chr 4	67595991	67696109	67645491	67646109
	miR172D	Chr 2	22151215	22251302	22200715	22201302
	miR172E	Chr 2	14072957	14173071	14122457	14123071

	miR172F	Chr 5	19245951	19346068	19295451	19296068
Zea mays	miR172A	Chr 7	54226707	54326789	54276207	54276789
	miR172B	Chr 5	221671349	221771474	221720849	221721474
	miR172C	Chr 4	174104928	174205050	174154428	174155050
	miR172D	Chr 6	171979786	172079859	172029286	172029859
	miR172E	Chr 3	145751592	145851765	145801092	145801765
Arabidopsis thaliana	miR172A	Chr2	11892914	11993015	11942414	11943015
	miR172B	Chr5	1138207	1238301	1187707	1188301
	miR172C	Chr3	3549776	3649908	3599276	3599908
	miR172D	Chr3	20537904	20638027	20587404	20588027
	miR172E	Chr5	23938472	24038596	23987972	23988596

cells

MDPI

Table S2: Forward and reverse primer sequence for amplification of *MIR172A, B, C* and *D* from different poaceae members and Arabidopsis.

Primer name	Forward	Reverse	Amplicon size	Tm
Os-miR172A	TGGATGGAACGGTAGAGTCG	TGAAACGGAGGGAGTGACAAG	224	59
Os-miR172B	CACAGAAAGAGGGAGGGAGG	AGTAGAGAGTGTGATGCCGC	336	59.5
Os-miR172C	CCTGCACGTAGACAACCATG	GCGGGCCCTATAAATTCTGC	316	59
Os-miR172D (i)	ATCAAAGCAGGCCATGGATC	TCGATCTCCCTCTAGCTCCA	299	59
Os-miR172D(ii)	ATCAAAGCAGGCCATGGATC	GCACAAATTGGCGAGGATG	210	59
Ob-miR172A	ATCAAAGCAGGCCATGGATC	AAACAGTCGGTGCTTGCAG	155	58
Ob-miR172B	CACAGAAAGAGGGAGGGAGG	CCGGCAGTAGAGAGTGTGAT	341	59
Ob-miR172C	AAAGCTAATCCTGCCTGCC	TCGTTGTAGGGTCGGGAATC	294	60
Ob-mir172D	AAAGCAGGCCATGGATCGAG	CAAATTGGCGAGGATGAGAGG	204	60
Og-miR172A	AAAGCAGGCCATGGATCGAG	CAAATTGGCGAGGATGAGAGG	204	60
Og-miR172B	CACAGAAAGAGGGAGGGAGG	CCGGCAGTAGAGAGTGTGAT	341	59
Og-miR172C	AAAGCTAATCCTGCCTGCC	TGTAGGGTCGGGAATCCAAC	290	60
Og-mir172D	ATCAAAGCAGGCCATGGATC	CAAATTGGCGAGGATGAGAG	207	59
Ou-miR172A	TGGATGGAACGGTAGAGTCG	AACGGAGGGAGTAACAAGG	222	58
Ou-miR172B	CACAGAAAGAGGGAGGGAGG	AGTAGAGAGTGTGATGCCGC	332	60
Ou-miR172C	AAAGCTAATCCTGCCTGCCT	TGTAGGGTCGGGAATCCAAC	290	59
Ou-mir172D	ATCAAAGCAGGCCATGGATC	CCCTCTAGCTCCATTCTCCA	296	58
Op-miR172A	TATAGTCGGTGTTTGCGGGT	TTATGTATTTGGGGACGG	257	59
Op-miR172B	ACCTGCTAGCCAATGTGTCA	GACCTGGCATTGCATACCTG	224	59
Op-miR172C	AGGAAGGGGAAAGCTAGGAAC	GCAGCCGCGGTTATATATGG	234	58
Op-mir172D	CCATGGCGACGAGTAGTAGT	CCGAATCTCCCTCTAGCTCC	268	59
Or-miR172A	AACCCCTAGCTAGAAGAGGC	CGGAGGGAGTAACAAGGATG	347	58
Or-miR172B	CACAGAAAGAGGGAGGGAGG	CCGGCAGTAGAGAGTGTGAT	341	59
Or-miR172C	CCTGCACGTAGACAACCATG	GTAGGGTCGGGAATCCAACA	268	59
Or-mir172D	ATCAAAGCAGGCCATGGATC	TCGATCTCCCTCTAGCTCCA	299	59
Oa-miR172A	GGACTCTCCCTTTTCCCTCC	TGGGCTGTTGAGAGAAGAAG	227	59
Oa-miR172B	AGCTGCTAGGATGAACTCGA	ATCTGAGAATAGACGGATCGAAG	157	58
Oa-miR172C	TCAGTAGAGGAGTGCTTGCC	GATTCCATCCAATCCAGCGAG	216	59
Oa-mir172D	GGCCAGGGGAAGCTATAACT	AGACGAAGCAGCAAACAGTC	244	58
Sb-miR172A	ATTCGGCGTCTCTCACTGTC	ACAACCTTAGCTTCCACATC	284	58
Sb-miR172B	GCACTGCTAGCTGATCTGAG	TATAGCAGCTGGAGACGACG	343	58.5
Sb-miR172C	CGGGTGATGAGTGAGTGTCT	CGTCTTGCCGGATCTCATTG	271	59
Sb-mir172D	GCCGTTCACCTCTTTCTCAC	GGATGGCACGATCTTTCACC	331	58
Zm-miR172A	CACCAAGCACCCAAACAAGA	TGCAGTTGATTACCATGGCG	250	58
Zm-miR172B	CCATGTACCTCTCACCCCAG	ACTACGCAACCCTTCCACAA	274	59
Zm-miR172C	TTTCCGACTTAAGGCATCCG	CCGTAGTGCATGCTCTCAAG	211	59
Zm-mir172D	CTGTTGACACGGACTTGGTC	AGCTCTTTCCGGGACAGAAG	259	59

At-miR172A	GGGAGAGAGAGAGAGAGGGA	TGGGTTTGAGTTTGAGTTTGAG	247	58
At-miR172B	TTGTAGCCGTCGATTGTTGA	GATCTCTTGTGCGTGCGTAA	185	58.5
At-miR172C	ACCTCCGATCTGTGAATTCCT	ATGGTGCCGTCTTGAGTCTT	279	59
At-mir172D	GGGTTAGCATGTTGATGACTTCA	TCGGAGGATTCTACATATGAGGA	243	58

Table S3. Forward primer sequence for Rt-qPCR used for generating the expression profile of *MIR172* in different members of poaceae and Arabidopsis.

MIRNA	Forward Primer
**Os-miR172**	GAATCTTGATGATGCTGC

Supplementary Data 1

Nucleotide sequence of PCR amplified and Sanger sequenced *MIR172 A-D* from different poaceae members

>Os-miR172A
TGGATGGAACGGTAGAGTCGGTGTTTGCGGGCGTGGCATCATCAAGATTCACATCCATGCATATA
TCACAAGACGCACATATACATCCGATTTGGCTGAGAATCTTGATGATGCTGCATCCGCAGACAAG
CGCCTTTTTTTCTTTTCAAAGGTAAGCTCCTCTGCTATTCAAAGACCATACATTAGTTGTTCATCAT
CTTCATCCTTGTCACTCCCTCCGTTTCA

>Os-miR172B
CACAGAAAGAGGGAGGGAGGATCACTCTCGCCGGCGGCGTTGGTACAATTAAGCTGATGCTTAG
TCATTAATTTCCGATGCAGCATCATCAAGATTCCCACACATATATACACATGCGCTTAATTAGCTT
CGATCTCTCTCTTCTCTCTCCATGCGTGCAACCATGCATCATGCAGGTCAGGGCCAGGAGAGAAA
TATTAGCTAGCTAGGTTGTTGAGATCAGAACAACAAGCTCAAAGATGCATAGACATATGCATGC
ATGCAATGTAATGTGTGAATCTTGATGATGCCACGTCAGAAATCACCGACTGCTAGCTGCGGCAT
CACACTCTCTACT

>Os-miR172C
CCTGCACGTAGACAACCATGCACACATATGCTTCCTGAAACAATACTAATAGAACAATATATAT
ACGGCCGGCTCTTTGCTGGTGCAGCATCATCAAGATTCACGCACGATGACTACACTTGAAAACCG
ATACGTGTGCCGTGCGGCACACGTGAATCTTGATGACGCTGCACCCGCAACAAGTCGGCTCAGCT
CGCATCCACCGCGCGCGCCGCCATGACGAATTGGCGACGGCGGCGGCGCGCGGCCTGTTGGATT
CCCGACCCTACAACGAACGGGCTGTGGCCTGTGGGGGCAGCAGAATTTATAGGGCCCGC

>Os-miR172Di
ATCAAAGCAGGCCATGGATCGAGACGAGTAGTCGAGCGTTTGCTGATGCAGCATCATCAAGATT
CTCATCACATATAGTCAGCCAACCTCGATCTCGTTTAGGGATGAACTCGATGTGAATCTTGATGGT
GCTGCACCTGCAAGCACCGACTGTTTGCTACTAGCTCCATATATGCATCGATCCATGCCTCTCATC
CTCGCCAATTTGTGCCAATTTGTGAGATCGATGCATATATATGGTGCATACACTACCAGAAGAAT
GGATAGTATATATGGAGAATGGAGCTAGAGGGAGATCGA

>Os-miR172Dii
ATCAAAGCAGGCCATGGATCGAGACGAGTAGTCGAGCGTTTGCTGATGCAGCATCATCAAGATT
CTCATCACATATAGTCAGCCAACCTCGATCTCGTTTAGGGATGAACTCGATGTGAATCTTGATGGT
GCTGCACCTGCAAGCACCGACTGTTTGCTACTAGCTCCATATATGCATCGATCCATGCCTCTCATC
CTCGCCAATTTGTGC

>Ob-miR172A
ATCAAAGCAGGCCATGGATCGAGACGAGTAGTCGAGCGTTTGCTGATGCAGCATCATCAAGATT
CTCATCACATATAGTCAGCCAACCTCGATCTCGTTTAGGGATGAACTCGATGTGAATCTTGATGGT
GCTGCACCTGCAAGCACCGACTGTTT

>Ob-miR172B
CACAGAAAGAGGGAGGGAGGATCACTCTCGCCGGCGGCGTTGGTACAATTAAGCTGATGCTTAG
TCATTAATTTCCGATGCAGCATCATCAAGATTCCCACACATATATACACATGCGCTTAATTAGCTT

CGATCTCTCTCTTCTCTCTCCATGCGTGCAACCATGCATCATGCAGGTCAGGGCCGGGAGAGAAA
TATTAGCTAGCTAGGTTGTTGAGATCAGAACAACAAGCTCAAAGATGCATAGACATATGCATGC
ATGCAATGTAATGTGTGAATCTTGATGATGCCACGTCAGAAATCACCGACTGCTAGCTGCGGCAT
CACACTCTCTACTGCCGG

>Ob-miR172C
AAAGCTAATCCTGCCTGCCTGCCTGCACGTAGACAACCATGCACACATATGCTTCCTGAAACAAT
ACTAATAGAACAATATATATACGGCCGGCTCTTTGCTGGTGCAGCATCATCAAGATTCACGCACG
ATGACCACACTTGAAAACCGATACGTGTGCCGTGCGGCACACGTGAATCTTGATGACGCTGCAC
CCGCAACAAGTCGGCTCAGCTCGCATCCACCGCGCGCGCCGCCATGACGAATTGGCGACGGCGG
CGGCGCGCGGCCTGTTGGATTCCCGACCCTACAACGA

>Ob-miR172D
AAAGCAGGCCATGGATCGAGACGAGTAGTCGAGCGTTTGCTGATGCAGCATCATCAAGATTCTC
ATCACATATAGTCAGCCAACCTCGATCTCGTTTAGGGATGAACTCGATGTGAATCTTGATGGTGC
TGCACCTGCAAGCACCGACTGTTTGCTACTAGCTCCATATATGCATCGATCCATGCCTCTCATCCT
CGCCAATTTG

>Og-miR172A
AAAGCAGGCCATGGATCGAGACGAGTAGTCGAGCGTTTGCTGATGCAGCATCATCAAGATTCTC
ATCACATATAGTCAGCCAACCTCGATCTCGTTTAGGGATGAACTCGATGTGAATCTTGATGGTGC
TGCACCTGCAAGCACCGACTGTTTGCTACTAGCTCCATATATGCATCGATCCATGCCTCTCATCCT
CGCCAATTTG

>Og-miR172B
CACAGAAAGAGGGAGGGAGGATCACTCTCGCCGGCGGCGTTGGTACAATTAAGCTGATGCTTAG
TCATTAATTTCCGATGCAGCATCATCAAGATTCCCACACATATATACACATGCGCTTAATTAGCTT
CGATCTCTCTCTTCTCTCTCCATGCGTGCAACCATGCATCATGCAGGTCAGGGCCGGGAGAGAAA
TATTAGCTAGCTAGGTTGTTGAGATCAGAACAACAAGCTCAAAGATGCATAGACATATGCATGC
ATGCAATGTAATGTGTGAATCTTGATGATGCCACGTCAGAAATCACCGACTGCTAGCTGCGGCAT
CACACTCTCTACTGCCGG

>Og-miR172C
AAAGCTAATCCTGCCTGCCTGCCTGCACGTAGACAACCATGCACACATATGCTTCCTGAAACAAT
ACTAATAGAACAATATATATACGGCCGGCTCTTTGCTGGTGCAGCATCATCAAGATTCACGCACG
ATGACCACACTTGAAAACCGATACGTGTGCCGTGCGGCACACGTGAATCTTGATGACGCTGCAC
CCGCAACAAGTCGGCTCAGCTCGCATCCACCGCGCGCGCCGCCATGACGAATTGGCGACGGCGG
CGGCGCGCGGCCTGTTGGATTCCCGACCCTACA

>Og-miR172D
ATCAAAGCAGGCCATGGATCGAGACGAGTAGTCGAGCGTTTGCTGATGCAGCATCATCAAGATT
CTCATCACATATAGTCAGCCAACCTCGATCTCGTTTAGGGATGAACTCGATGTGAATCTTGATGGT
GCTGCACCTGCAAGCACCGACTGTTTGCTACTAGCTCCATATATGCATCGATCCATGCCTCTCATC
CTCGCCAATTTG

>Ou-miR172A

TGGATGGAACGGTAGAGTCGGTGTTTGCGGGCGTGGCATCATCAAGATTCACATCCATGCATATA
TCACAAGACGCACATATACATCCGATTTGGCTGAGAATCTTGATGATGCTGCATCCGCAGACAAG
CGCCTTTTTTTTCTTTTCAAAGGTAAGCTCCTCTGCTATTCAAAGACCATACATTAGTTGTTCATCA
TCTTCATCCTTGTTACTCCCTCCGTT

>Ou-miR172B
CACAGAAAGAGGGAGGGAGGATCACTCTCGCCGGCGGCGTTGGTACAATTAAGCTGATGCTTAG
TCATTAATTTCCGATGCAGCATCATCAAGATTCCCACACATATATACACATGCGCTTAATTAGCTT
CGATCTCTCTCTTCTCTCTCCATGCGTGCAACCATGCATCATGCAGGTCAGGGCCGGGAGAGAAA
TATTAGCTAGGTTGTTGAGATCAGAACAACAAGCTCAAAGATGCATAGACATATGCATGCATGC
AATGTAATGTGTGAATCTTGATGATGCCACGTCAGAAATCACCGACTGCTAGCTGCGGCATCACA
CTCTCTACT

>Ou-miR172C
AAAGCTAATCCTGCCTGCCTGCCTGCACGTAGACAACCATGCACACATATGCTTCCTGAAACAAT
ACTAATAGAACAATATATATACGGCCGGCTCTTTGCTGGTGCAGCATCATCAAGATTCACGCACG
ATGACCACACTTGAAAACCGATACGTGTGCCGTGCGGCACACGTGAATCTTGATGACGCTGCAC
CCGCAACAAGTCGGCTCAGCTCGCATCCACCGCGCGCGCCGCCATGACGAATTGGCGACGGCGG
CGGCGCGCGGCCTGTTGGATTCCCGACCCTACA

>Ou-miR172D
ATCAAAGCAGGCCATGGATCGAGACGAGTAGTCGAGCGTTTGCTGATGCAGCATCATCAAGATT
CTCATCACATATAGTCAGCCAACCTCGATCTCGTTTAGGGATGAACTCGATGTGAATCTTGATGGT
GCTGCACCTGCAAGCACCGACTGTTTGCTACTAGCTCCATATATGCATCGATCCATGCCTCTCATC
CTCGCCAATTTGTGCCAATTTGTGAGATCGATGCATATATATGGTGCATACACTACCAGAAGAAT
GGATAGTATATATATATGGAGAATGGAGCTAGAGGG

>Op-miR172A
TATAGTCGGTGTTTGCGGGTGTGGCATCATCAAGATTCACATCCACGCATATATCAAAGAAGCAC
AGATATAGATTTTCACATCCGATTTGGCTGAGAATCTTGATGATGCTGCATCCGCAGACAAGCGC
CTTTTTATTTTTTTGAAAGGTAAGTAGGAGCTGTGCTCTTCTGTTATTCATCATCATTCAAAGACCA
TACATCAATTGTTCATCATCTTCATCCTAGTTATTATATATGGCCGTCCCCAAATACATAA

>Op-miR172B
ACCTGCTAGCCAATGTGTCAGACTAAAGAGGGAGAGGATCATATCAGAGCTCTCGCCGGCGGTG
TTGATCAACGGTACAGTATTTTAAGCCGGCCTTCACAATCGTGGAAGGTGAATTGATTTTTAGTCA
TTAATTTCCGATGCAGCATCATCAAGATTCCCACACATATATACATCTCTCTGTTCTTCCTGGCAA
GCAACCATGCAGGTATGCAATGCCAGGTC

>Op-miR172C
AGGAAGGGGAAAGCTAGGAACTGTACCTGAAGATTAATTTCCAGTCTCCAATAATCAAGCAGGC
CATGGCGACGAGTAGTAGTCGAGCGATTGCTGATGCAGCATCATCAAGATTCTCATCACATATAT
AGTCAGCCACCTCGATCTCGTTTAGGGATGAACTCGATGTGAATCTTGATGGTGCTGCACCTGCA
AGCACCGACTGTTTGCTAGCTCCATATATAACCGCGGCTGC

>Op-miR172D

CCATGGCGACGAGTAGTAGTCGAGCGATTGCTGATGCAGCATCATCAAGATTCTCATCACATATA
TAGTCAGCCACCTCGATCTCGTTTAGGGATGAACTCGATGTGAATCTTGATGGTGCTGCACCTGC
AAGCACCGACTGTTTGCTAGCTCCATATATAACCGCGGCTGCCTCTCATCCTCGCCAAGTTGCGA
GATCGATGCATGGTGCATACACTACCAGAAGAATGGATAATATAAATGGAGAATGGAGCTAGA
GGGAGATTCGG

>Or-miR172A
AACCCCTAGCTAGAAGAGGCTAGAGAAGAGCAAATCTCTCCCTTCTCCCTTGTGTCCAATGCATC
TCTTGAATTTACTGTACTCTGGAGTAGAAGATACGAACAATGCTCAGTAGTACAGTAAATCCATG
GATGGAACGGTAGAGTCGGTGTTTGCGGGCGTGGCATCATCAAGATTCACATCCATGCATATATC
ACAAGACGCACATATACATCCGATTTGGCTGAGAATCTTGATGATGCTGCATCCGCAGACAAGC
GCCTTTTTTTCTTTTCAAAGGTAAGCTCCTCTGCTATTCAAAGACCATACATTAGTTGTTCATCATC
TTCATCCTTGTTACTCCCTCCG

>Or-miR172B
CACAGAAAGAGGGAGGGAGGATCACTCTCGCCGGCGGCGTTGGTACAATTAAGCTGATGCTTAG
TCATTAATTTCCGATGCAGCATCATCAAGATTCCCACACATATATACACATGCGTTTAATTAGCTT
CGATCTCTCTCTTCTCTCTCCATGCGTGCAACCATGCATCATGCAGGTCAGGGTCAGGAGAGAAA
TATTAGCTAGCTAGGTTGTTGAGATCAGAACAACAAGCTCAAAGATGCATAGACATATGCATGC
ATGCAATGTAATGTGTGAATCTTGATGATGCCACGTCAGAAATCACCGACTGCTAGCTGCGGCAT
CACACTCTCTACTGCCGG

>Or-miR172C
CCTGCACGTAGACAACCATGCACACATATGCTTCCTGAAACAATACTAATAGAACAATATATAT
ACGGCCGGCTCTTTGCTGGTGCAGCATCATCAAGATTCACGCACGATGACTACACTTGAAAACCG
ATACGTGTGCCGTGCGGCACACGTGAATCTTGATGACGCTGCACCCGCAACAAGTCGGCTCAGCT
CGCATCCACCGCGCGCGCCGCCATGACGAATTGGCGACGGCGGCGCGCGGCCTGTTGGATT
CCCGACCCTAC

>Or-miR172D
ATCAAAGCAGGCCATGGATCGAGACGAGTAGTCGAGCGTTTGCTGATGCAGCATCATCAAGATT
CTCATCACATATAGTCAGCCAACCTCGATCTCGTTTAGGGATGAACTCGATGTGAATCTTGATGGT
GCTGCACCTGCAAGCACCGACTGTTTGCTACTAGCTCCATATATGCATCGATCCATGCCTCTCATC
CTCGCCAATTTGTGCCAATTTGTGAGATCGATGCATATATATGGTGCATACACTACCAGAAGAAT
GGATAGTATATATGGAGAATGGAGCTAGAGGGAGATCGA

>Oa-miR172A
GGACTCTCCCTTTTCCCTCCTACTTGCTTAGATGCATATATCATGGCTGGTTTAGTCGGTGTTTGCG
GGCGTGGCATCATCAAGATTCACATCTATGCACCGATAGATCGCTCTTTACGACATAGATTTCAG
ACCCGTTTTGGCTAGCTGGATCTGGATGAGAATCTTGATGATGCTGCATCCGCAGACAAGCGCCT
TCGATTCCTCTCTTCTTCTCTCAACAGCCCA

>Oa-miR172B
AGCTGCTAGGATGAACTCGATAGCTGGCGGCGTTGAATTGAAGGTAGAATGTGACTTGATTTTTA
GTCATTAATTTCCGATGCAGCATCATCAAGATTCCCACACGTATATATGTACAAACTTGATCAGT
AGCTTCTTCGATCCGTCTATTCTCAGAT

>Oa-miR172C
TCAGTAGAGGAGTGCTTGCCGTAATAATATATAGAAGAATAAACAACACGGCCGGCTCTTTGCC
GATGCAGCATCATCAAGATTCACGGACGACTGGGACCGACCGACATGCATGCACGATGCCACGC
CACCAGGCCGGCGGCGCACACGTGAATCTTGGTGATGCCGCACCCGCAACAAGTCGGCCTCGCG
CTCGCTCGCTGGATTGGATGGAATC

>Oa-miR172D
GGCCAGGGGAAGCTATAACTGTATATAAGTAAAAGCGTACCTGAGTCTCCAATTAAGCCGGCCA
TGGCGACGAGTGTAGTCGAGCGTTTGCTGATGCAGCATCATCAAGATTCTCATCGCATATAGTCA
GCCACACCTCGATCCCTCTCTCTCTCTCACTCTCTCTCTCTCTTTTAGGATGAACTCGACGATGTGA
ATCTTGATGGTGCTGCACCTGCAAGCACCGACTGTTTGCTGCTTCGTC

>Sb-miR172A
ATTCGGCGTCTCTCACTGTCCAGGTGGGATGAGTGAGTGTTTGCTGATGCAGCATCATCAAGATTC
TCATCTCATCACATACGCATATATTTATGTATATAGCATATCACCGAGGATGAGCTGGATGTGAA
TCTTGATGATGCTGCACCTGCAAGCACCGACTGTGTTTGCTTGCGTGCTTGCTTCATAATCTGTAG
CAGCTAACTTCAATTCTCCAAGTTCTTCAGGAAAGATGAGGATTTTATTTTTATTTTTTGAGATTAA
AGATGTGGAAGCTAAGGTTG

>Sb-miR172B
GCACTGCTAGCTGATCTGAGATGGATCGATAGCCCTTGATCTCCAATGCAGCATCATCAAGATTC
CCACACATACACATATATATTCACAGAGGCATATATGATGCAGCAGCAGCTAAAACATGTAGTA
AAACTGCTATTAATGGCAGGCAAAGAAGGCGGCAGAGATGCATGTATGCATTTGCAAGCAGTGT
GTGAATCTTGATGATGCCACGCCAGAAATCACCGGCTGATGATGATAATACATCTCTCTCTCTCTC
TCTCTCTCTCTCTCTCTCTCTCTCTCTCTCTCTCTCTCTCTCTCTCTCTCTCTCCATATACGTATCGTC
GTCTCCAGCTGCTATA

>Sb-miR172C
CGGGTGATGAGTGAGTGTCTGCTGATGCAGCATCATCAAGATTCTCATCTCATCACATATGCATA
TATAGCAACTGCGAGCCATCACATGAGCATCACTGAAGATGCATGAGTTGGACCATGTGAATCTT
GATGGTGCTGCACCTGCAAGCACCCACTGTTTGCTTGCTTGCTTGCTTTCCTGCTTGTTGCTTCATT
CATATACAATACAATCCATATATGGCAGCTAGCTTCTGCATGCGGCTTCGATCTCCAATGAGATC
CGGCAAGACG

>Sb-miR172D
GCCGTTCACCTCTTTCTCACCGATGCCACATATATATAGCACCACCACAGACTCCTAGCCCGGAT
TAGTCTAGCATTGCACAGTCGGTGTTTGCGGGCGTGGCATCATCAAGATTCACAACCCATGGATC
CAAATAAACCACTGATTTGGATCGAGGAGATGCATGAGAATCTTGATGATGCTGCATCCGCCAA
CAAGCGCCTACCATATATACCACCTCCTTCTGGATCTCTACTTCTCTAATGTAAGTAGTCTCAGAC
AGTATTGTGCTCATCTTCATCATCAATCGAGATCGCCCTCACGGTTAATTAAGGTGAAAGATCGT
GCCATCC

>Zm-miR172A
CAGAGAAAGGAAATATATAGAAATGGTCGTTGTTTCGAATAAAAATTTGCAAAGCTAGCTTTAG
GCGGATGGGATGACGATCTTTCACCTTAGCTAAACGTGAAGATACAAAATCTCGATTGATGGCG
AAGATGAGCACACAAACGTTCGTAGGCGCTTGTTGGCGGATGCAGCATCATCAAGATTCTCATG
CATGCATTCCAAATCAGTGGTTCGGATTGATGGGTTGTGAATCTTGATGATGCCACGC

>Zm-miR172B
AAGTTGATTAGCAAACAAGGTAGACTGAATGGAAAAAGAAATTAGAGGAGAACGAAGTGGTAC
GTACCAGAGCTAGTAGCTGCATGGTCGTTAATTCAATTCATCTCTTATTACTTGGAGGTGATGCAT
GAGCTAGTGAGTGTCTGCTGATGCAGCATCATCAAGATTCTCATCTCATCATATATATATACACA
CACATGCGAGGCACGCATCATTGAAGATGCATATATGCATGATGCATGAGTTGGACGATGTGAA
TCTTGATGGTGCTGCAC

>Zm-miR172C
TTTGTTAATGCATACATTCATATATATATTCCCATGAATGAATGCTTTAAGCATGAGGCAAGCAA
ACATCGACAGTGGGTGCTTGCAGGTGCAGCACCACCAAGATTCACATCCAACTCTCACGCATCTT
CAGTGATGCATGCATGCTCTGTGATGTCTCGCAGCAGCTATATGCATATGTGATGAGATGAGAAT
CTTGATGATGCTGCAT

>Zm-miR172D
TCTTCTTGATTCATAGGCTCATCATCTATGCCTCTGTCATGCACACACTCGTGTCTTTCTTCTTGAT
GGATACACGTACGGGGGGTTGGGTTGTTCACATATATAGTAGTATAGCTAGTTTATTAGATGCAG
GTATACAGATCATGAGGAAGCAAGAAATTATGCAAAACAGTCGGTGCTTGCAGGTGCAGCACCA
TCAAGATTCACATCCCCAGCTCGATCTGTGCATGATGAGATGAGAATCTTGATGATGCTGCAT

>At-miR172A
GGGAGAGAGAGAGAGAGAGGGAAAGAATAGTCGTTGATTGCCGATGCAGCATCATCAAGATTCTC
ATTTTCATAGAGAACTTTGTGGAGAGTGAATCACCACCGTCCATCAACAGATGTGAATCTTGATG
ATGCCACAGCAGCCAACAACGACCGTGGCTCCGGAAATTAGTCTTCCATTTTTGTTTAACAATCT
TCTTGTAGTAATCTTTTGTCTCTCAAACTCAAACTCAAACTCAAACTCAAACCCA

>At-miR172B
TTGTAGCCGTCGATTGTTGATGCAGCATCATCAAGATTCTCATATACATATCAAAACCCTAATTTA
GGGTATTTATCAATTTCCATGTGAATCTTAATGGTGCTGCGCCTACAAACAACGACAGATGAGCT
TTCTTCCATGGATCCGCCGACAACTTGTATAAAATTTACGCACGCACAAGAGAT

>At-miR172C
ACCTCCGATCTGTGAATTCCTACAAAAAGATTGGACTATGAAATTTCCAATCAACACAAACCTGT
TTCTATGTGTAAGCCACTGATTGCAGCTGCAGCATCATCAAGATTCTCATACATACATCAAAAAT
CTAATCATAAATGGGTTTATTTACACGAATACTTGATGATTTGTGAATCTTGATGATGCTCCAACA
GCGAACAGTAGCTGGTTGATGATAGGGATGTATGTAGTGATTTGGTTTTGTCTTGGATCTTTTCAA
GACTCAAGACGGCACCAT

>At-miR172D
GGGTTAGCATGTTGATGACTTCAGATTCTGAAGTTAGTGGCAGTCATTGTTTGCTATTGCAACATC
TTCAAGATTCAGAAATCAGATTCTCTTATGGGTTTTCTTTTGAGCCTTTATTTTTTGGTTTGAGAAT
CTTGATGATGCTGCAGCGGCAATTAAATGGCTTACTTATCTAGGGTCTTTTTTTCCATTTTCATCAT
GTGGTTAATCAGAAATCCAGATCCTCATATGTAGAATCCTCCGA

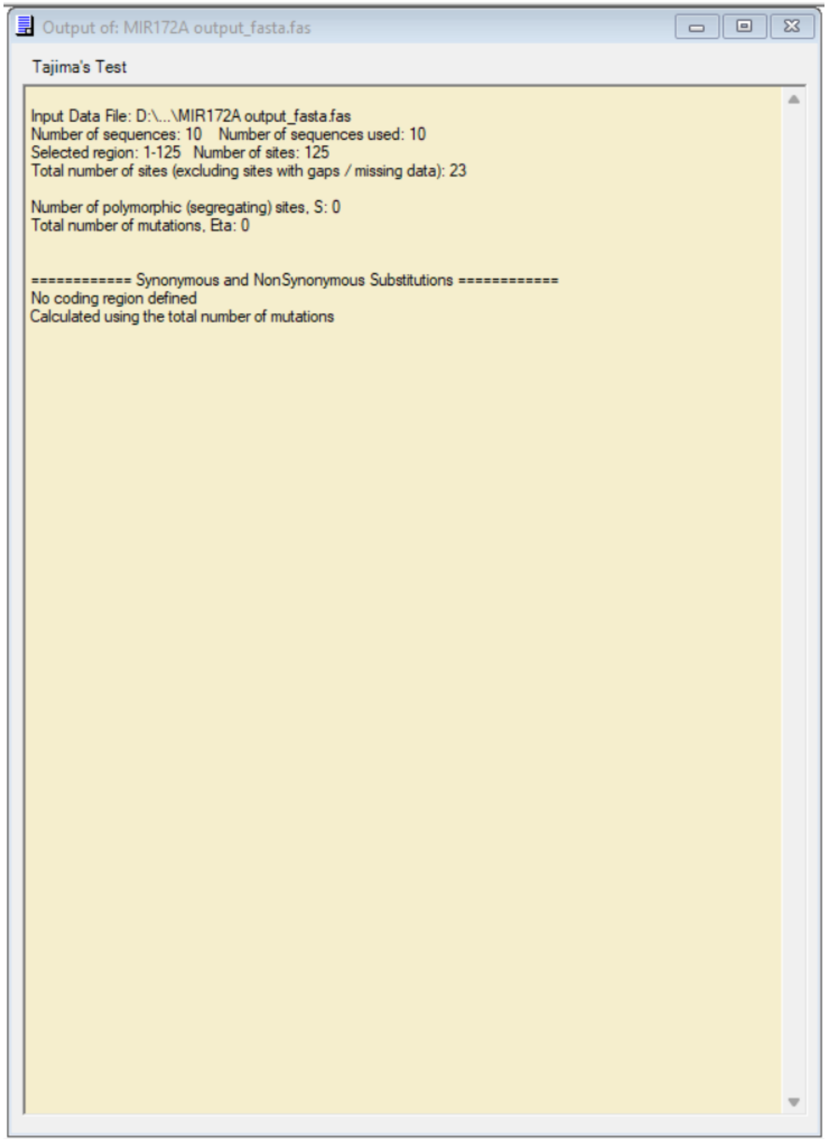

Figure S1. Screenshot of Tajima's test conducted on *MIR172A* sequences from *Oryza* spp, other poaceae members and Arabidopsis. A total of 10 sequences were included in the study.

cells

MDPI

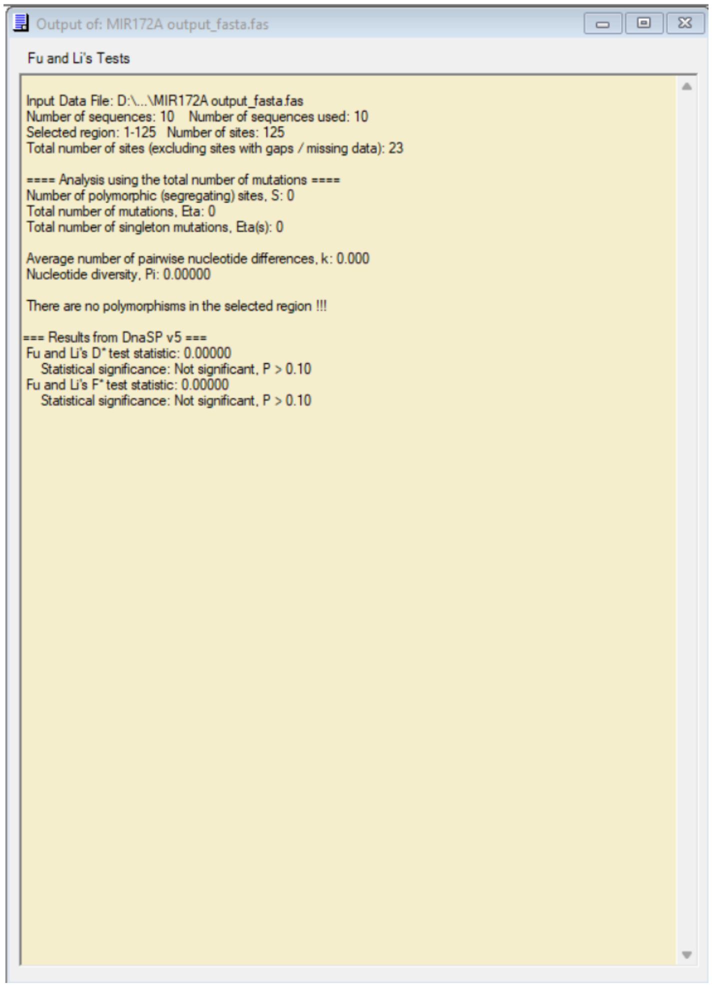

Figure S2. Screenshot of Fu and Li's test conducted on *MIR172A* sequences from *Oryza* spp, other poaceae members and Arabidopsis. A total of 10 sequences were included in the study.

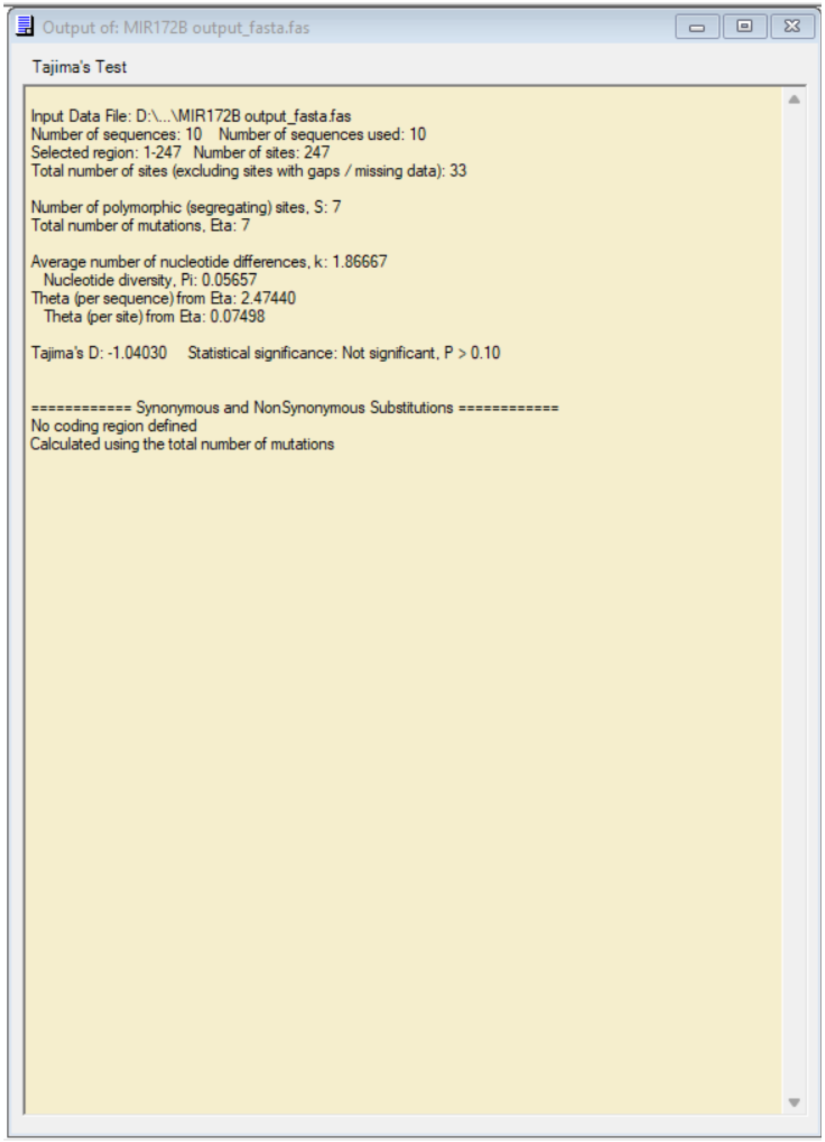

Figure S3. Screenshot of Tajima's test conducted on *MIR172B* sequences from *Oryza* spp, other poaceae members and Arabidopsis. A total of 10 sequences were included in the study.

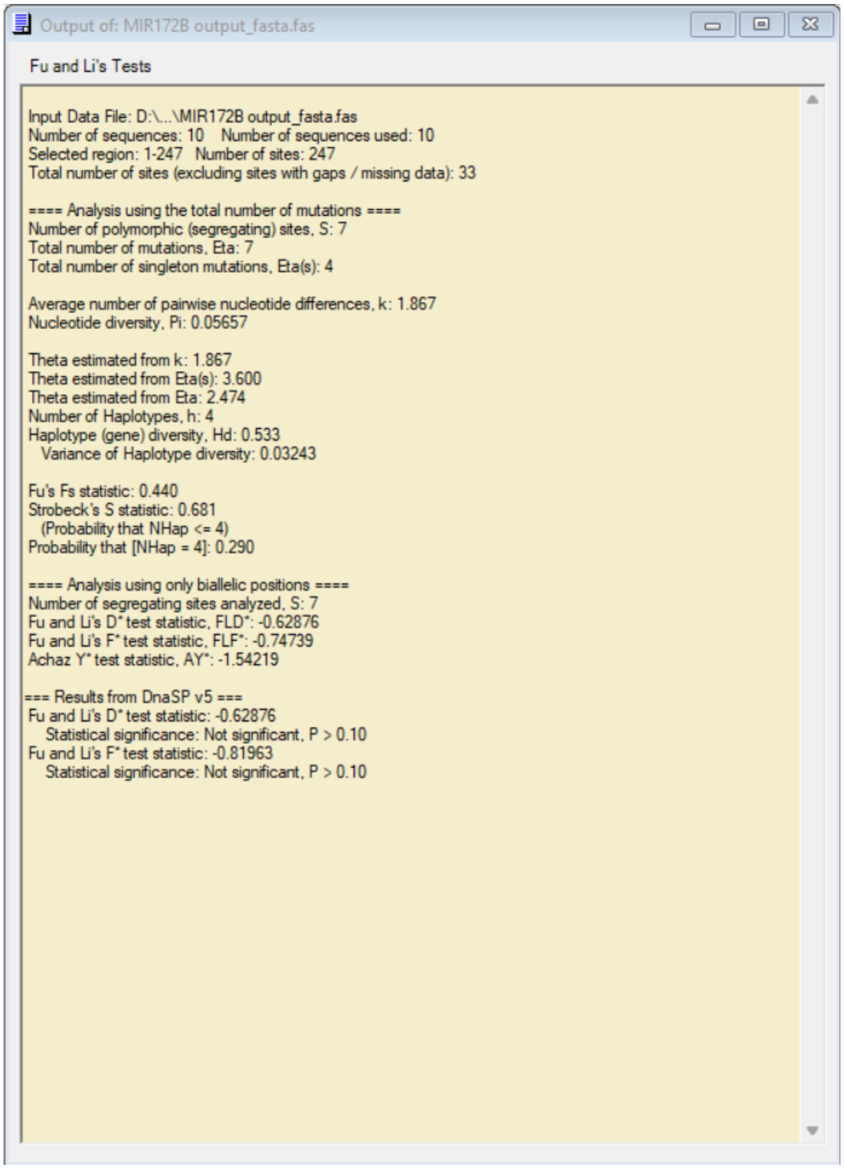

Output of: MIR172B output_fasta.fas

Fu and Li's Tests

Input Data File: D:\...\MIR172B output_fasta.fas
Number of sequences: 10 Number of sequences used: 10
Selected region: 1-247 Number of sites: 247
Total number of sites (excluding sites with gaps / missing data): 33

==== Analysis using the total number of mutations ====
Number of polymorphic (segregating) sites, S: 7
Total number of mutations, Eta: 7
Total number of singleton mutations, Eta(s): 4

Average number of pairwise nucleotide differences, k: 1.867
Nucleotide diversity, Pi: 0.05657

Theta estimated from k: 1.867
Theta estimated from Eta(s): 3.600
Theta estimated from Eta: 2.474
Number of Haplotypes, h: 4
Haplotype (gene) diversity, Hd: 0.533
Variance of Haplotype diversity: 0.03243

Fu's Fs statistic: 0.440
Strobeck's S statistic: 0.681
(Probability that NHap <= 4)
Probability that [NHap = 4]: 0.290

==== Analysis using only biallelic positions ====
Number of segregating sites analyzed, S: 7
Fu and Li's D* test statistic, FLD*: -0.62876
Fu and Li's F* test statistic, FLF*: -0.74739
Achaz Y* test statistic, AY*: -1.54219

=== Results from DnaSP v5 ===
Fu and Li's D* test statistic: -0.62876
Statistical significance: Not significant, P > 0.10
Fu and Li's F* test statistic: -0.81963
Statistical significance: Not significant, P > 0.10

Figure S4. Screenshot of Fu and Li's test conducted on *MIR172B* sequences from *Oryza* spp, other poaceae members and Arabidopsis. A total of 10 sequences were included in the study.

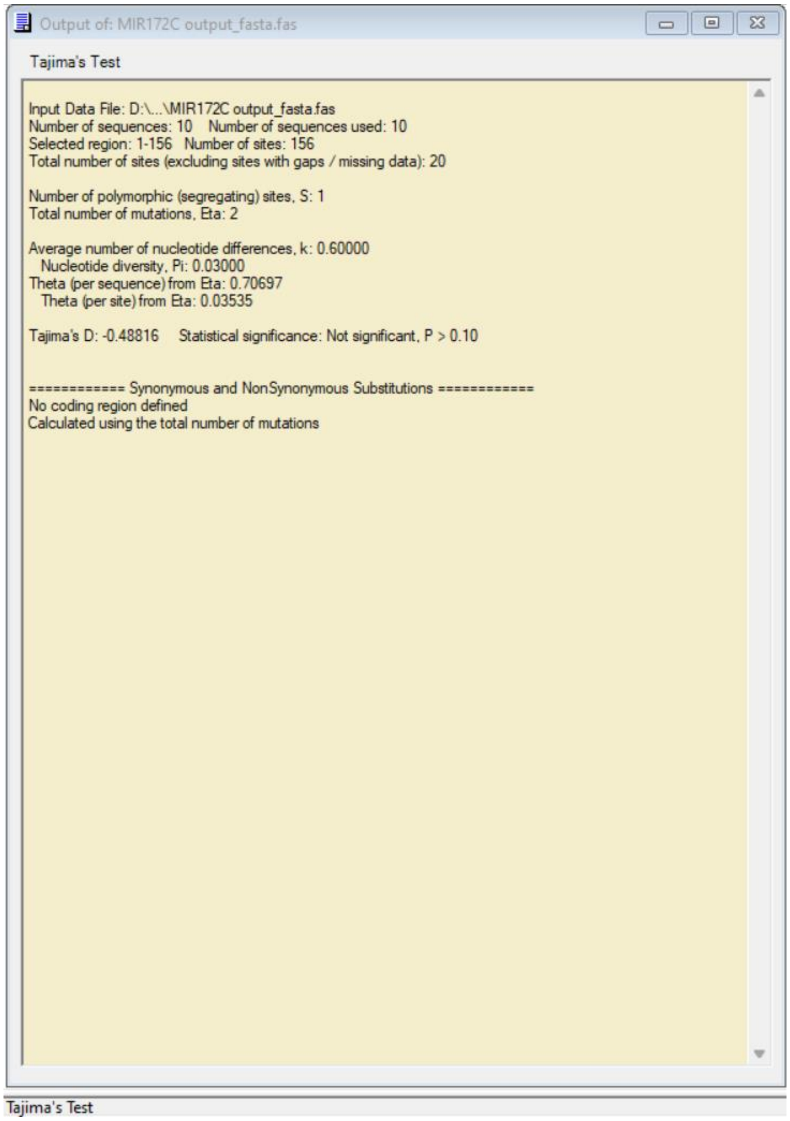

Figure S5. Screenshot of Tajima's test conducted on *MIR172C* sequences from *Oryza* spp, other poaceae members and Arabidopsis. A total of 10 sequences were included in the study.

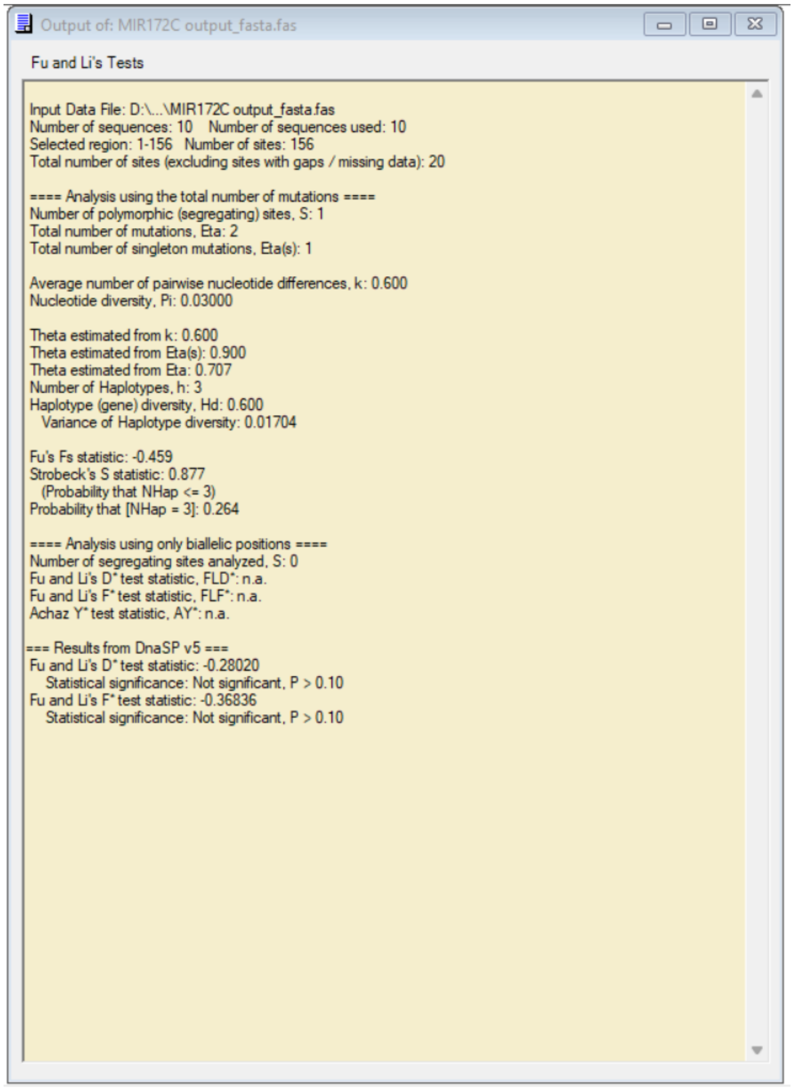

Figure S6. Screenshot of Fu and Li's test conducted on *MIR172C* sequences from *Oryza* spp, other poaceae members and Arabidopsis. A total of 10 sequences were included in the study.

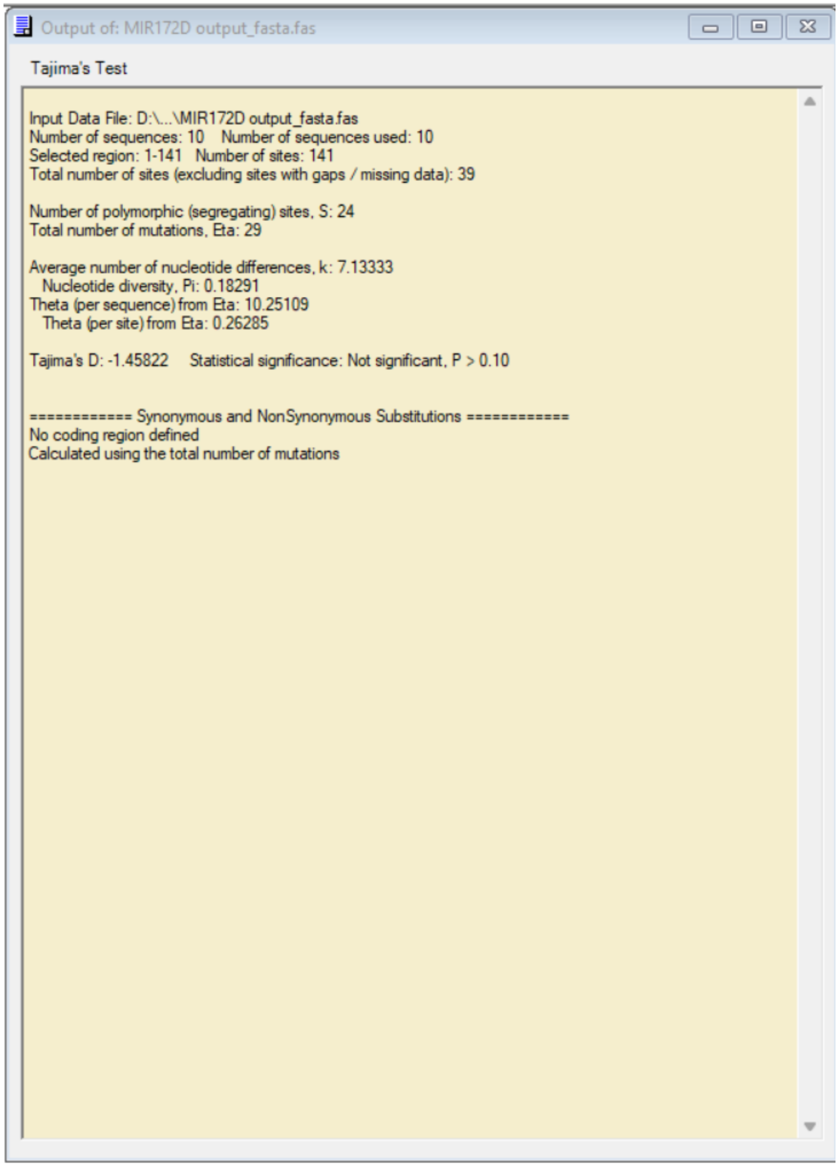

Output of: MIR172D output_fasta.fas

Tajima's Test

Input Data File: D:\...\MIR172D output_fasta.fas
Number of sequences: 10   Number of sequences used: 10
Selected region: 1-141   Number of sites: 141
Total number of sites (excluding sites with gaps / missing data): 39

Number of polymorphic (segregating) sites, S: 24
Total number of mutations, Eta: 29

Average number of nucleotide differences, k: 7.13333
  Nucleotide diversity, Pi: 0.18291
Theta (per sequence) from Eta: 10.25109
  Theta (per site) from Eta: 0.26285

Tajima's D: -1.45822   Statistical significance: Not significant, P > 0.10

============ Synonymous and NonSynonymous Substitutions ============
No coding region defined
Calculated using the total number of mutations

Figure S7. Screenshot of Tajima's test conducted on *MIR172D* sequences from *Oryza* spp, other poaceae members and Arabidopsis. A total of 10 sequences were included in the study.

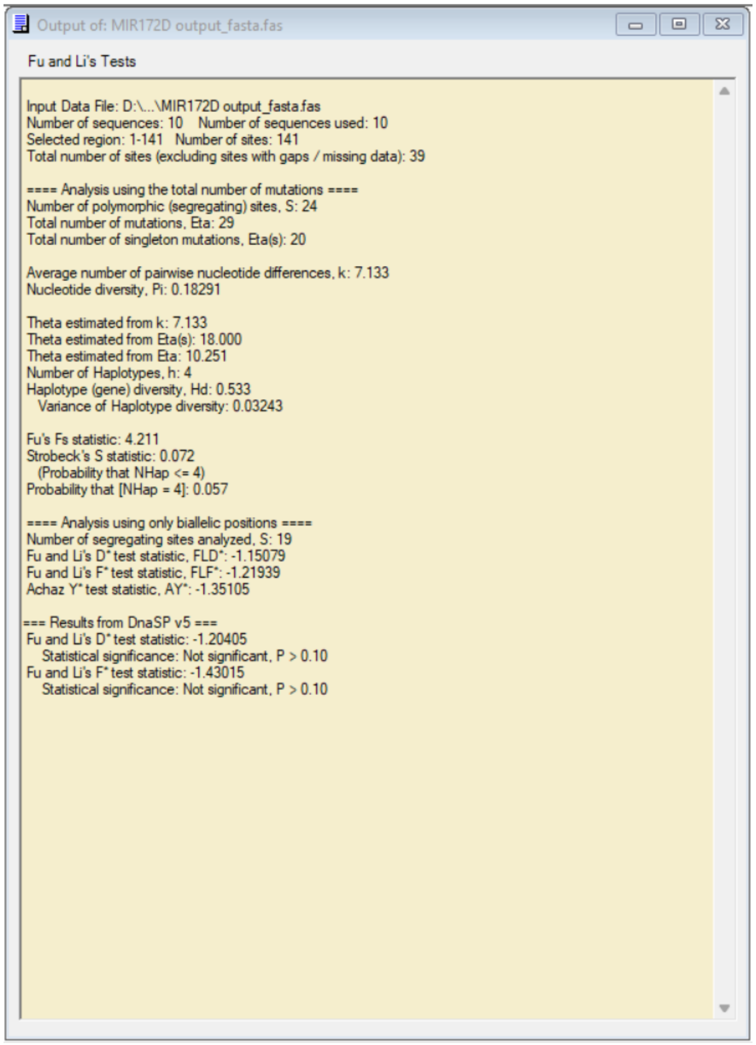

Figure S8. Screenshot of Fu and Li's test conducted on *MIR172D* sequences from *Oryza* spp, other poaceae members and Arabidopsis. A total of 10 sequences were included in the study.

Duplication depth	Reference chromosome	Collinear blocks
0	Sequence24Ob2g	
1	Sequence25Ob2g	Sequence46Og2g
1	Sequence26Ob2g	Sequence47Og2g
1	Sequence27Ob2g	Sequence48Og2g
1	Sequence28Ob2g	Sequence49Og2g
1	Sequence29Ob2g	Sequence50Og2g
1	Sequence30Ob2g	Sequence51Og2g
1	Sequence31Ob2g	||
1	Sequence32Ob2g	Sequence53Og2g
1	Sequence33Ob2g	Sequence55Og2g
1	Sequence34Ob2g	Sequence57Og2g
1	Sequence35Ob2g	Sequence58Og2g
1	Sequence36Ob2g	Sequence59Og2g
1	Sequence37Ob2g	Sequence60Og2g
1	Sequence38Ob2g	Sequence61Og2g
1	Sequence39Ob2g	Sequence62Og2g
1	Sequence40Ob2g	Sequence63Og2g
1	Sequence41Ob2g	Sequence64Og2g
1	Sequence42Ob2g	Sequence65Og2g
1	Sequence43Ob2g	Sequence66Og2g
1	Sequence44Ob2g	Sequence67Og2g
1	Sequence45Ob2g	Sequence68Og2g

Figure S9: Synteny block diagram for *MIR172A* with *Oryza barthii* as reference. The first column shows duplication depth at each gene locus, second column shows the genes in reference chromosomes and the following is aligned collinear blocks where only match genes are displayed. The alignment among non-anchor genes is discarded in the output and denoted by '||' in the multi-alignment of gene orders. Ob2- *Oryza barthii* chr 2, Og2- *Oryza glaberrima* chr 2.

Duplication depth	Reference chromosome	Collinear blocks
1	Sequence46Og2g	Sequence25Ob2g
1	Sequence47Og2g	Sequence26Ob2g
1	Sequence48Og2g	Sequence27Ob2g
1	Sequence49Og2g	Sequence28Ob2g
1	Sequence50Og2g	Sequence29Ob2g
1	Sequence51Og2g	Sequence30Ob2g
1	Sequence52Og2g	||
1	Sequence53Og2g	Sequence32Ob2g
1	Sequence54Og2g	||
1	Sequence55Og2g	Sequence33Ob2g
1	Sequence56Og2g	||
1	Sequence57Og2g	Sequence34Ob2g
1	Sequence58Og2g	Sequence35Ob2g
1	Sequence59Og2g	Sequence36Ob2g
1	Sequence60Og2g	Sequence37Ob2g
1	Sequence61Og2g	Sequence38Ob2g
1	Sequence62Og2g	Sequence39Ob2g
1	Sequence63Og2g	Sequence40Ob2g
1	Sequence64Og2g	Sequence41Ob2g
1	Sequence65Og2g	Sequence42Ob2g
1	Sequence66Og2g	Sequence43Ob2g
1	Sequence67Og2g	Sequence44Ob2g
1	Sequence68Og2g	Sequence45Ob2g

Figure S10: Synteny block diagram for *MIR172*A with *Oryza glaberrima* as reference. The first column shows duplication depth at each gene locus, second column shows the genes in reference chromosomes and the following is aligned collinear blocks where only match genes are displayed. The alignment among non-anchor genes is discarded in the output and denoted by '||' in the multi-alignment of gene orders. ob2- *Oryza barthii* chr 2, og2- *Oryza glaberrima* chr 2.

<table>
<tr><th>Duplication depth</th><th>Reference chromosome</th><th>Collinear blocks</th><th></th><th></th><th></th></tr>
<tr><td>1</td><td>Sequence69Ou9g</td><td>Sequence89Or9g</td><td></td><td></td><td></td></tr>
<tr><td>1</td><td>Sequence70Ou9g</td><td>Sequence90Or9g</td><td></td><td></td><td></td></tr>
<tr><td>2</td><td>Sequence71Ou9g</td><td>||</td><td>Sequence5Os9g</td><td></td><td></td></tr>
<tr><td>2</td><td>Sequence72Ou9g</td><td>||</td><td>||</td><td></td><td></td></tr>
<tr><td>4</td><td>Sequence73Ou9g</td><td>Sequence91Or9g</td><td>Sequence7Os9g</td><td>Sequence119Oa9g</td><td>Sequence147Op9g</td></tr>
<tr><td>4</td><td>Sequence74Ou9g</td><td>Sequence92Or9g</td><td>Sequence8Os9g</td><td>Sequence121Oa9g</td><td>Sequence148Op9g</td></tr>
<tr><td>4</td><td>Sequence75Ou9g</td><td>Sequence93Or9g</td><td>Sequence9Os9g</td><td>Sequence122Oa9g</td><td>Sequence149Op9g</td></tr>
<tr><td>4</td><td>Sequence76Ou9g</td><td>Sequence94Or9g</td><td>Sequence12Os9g</td><td>Sequence124Oa9g</td><td>Sequence150Op9g</td></tr>
<tr><td>4</td><td>Sequence77Ou9g</td><td>||</td><td>||</td><td>||</td><td>||</td></tr>
<tr><td>4</td><td>Sequence78Ou9g</td><td>||</td><td>||</td><td>||</td><td>||</td></tr>
<tr><td>4</td><td>Sequence79Ou9g</td><td>Sequence99Or9g</td><td>Sequence15Os9g</td><td>Sequence125Oa9g</td><td>Sequence151Op9g</td></tr>
<tr><td>4</td><td>Sequence80Ou9g</td><td>Sequence100Or9g</td><td>Sequence17Os9g</td><td>Sequence126Oa9g</td><td>Sequence152Op9g</td></tr>
<tr><td>4</td><td>Sequence81Ou9g</td><td>Sequence101Or9g</td><td>Sequence18Os9g</td><td>||</td><td>||</td></tr>
<tr><td>4</td><td>Sequence82Ou9g</td><td>Sequence102Or9g</td><td>Sequence19Os9g</td><td>Sequence129Oa9g</td><td>Sequence154Op9g</td></tr>
<tr><td>4</td><td>Sequence83Ou9g</td><td>Sequence103Or9g</td><td>Sequence20Os9g</td><td>Sequence130Oa9g</td><td>Sequence155Op9g</td></tr>
<tr><td>4</td><td>Sequence84Ou9g</td><td>Sequence104Or9g</td><td>Sequence21Os9g</td><td>Sequence131Oa9g</td><td>Sequence156Op9g</td></tr>
<tr><td>4</td><td>Sequence85Ou9g</td><td>Sequence105Or9g</td><td>Sequence22Os9g</td><td>Sequence132Oa9g</td><td>Sequence157Op9g</td></tr>
</table>

Figure S11: Synteny block diagram for *MIR172*A with *Oryza glumaepatula* as reference. The first column shows duplication depth at each gene locus, second column shows the genes in reference chromosomes and the following is aligned collinear blocks where only match genes are displayed. The alignment among non-anchor genes is discarded in the output and denoted by '||' in the multi-alignment of gene orders. Os9- *Oryza sativa* chr 9, Ou9- *Oryza glumaepatula* chr 9, Or9- *Oryza rufipogon* chr 9, Oa9- *Oryza brachyantha* chr 9, Op9- *Oryza punctata* chr 9.

<table>
<tr><th>Duplication depth</th><th>Reference chromosome</th><th>Collinear blocks</th><th></th><th></th><th></th></tr>
<tr><td>1</td><td>Sequence86Or9g</td><td>Sequence4Os9g</td><td></td><td></td><td></td></tr>
<tr><td>1</td><td>Sequence87Or9g</td><td>||</td><td></td><td></td><td></td></tr>
<tr><td>1</td><td>Sequence88Or9g</td><td>Sequence5Os9g</td><td></td><td></td><td></td></tr>
<tr><td>2</td><td>Sequence89Or9g</td><td>Sequence6Os9g</td><td>Sequence69Ou9g</td><td></td><td></td></tr>
<tr><td>2</td><td>Sequence90Or9g</td><td>||</td><td>Sequence70Ou9g</td><td></td><td></td></tr>
<tr><td>4</td><td>Sequence91Or9g</td><td>Sequence7Os9g</td><td>Sequence73Ou9g</td><td>Sequence147Op9g</td><td>Sequence119Oa9g</td></tr>
<tr><td>4</td><td>Sequence92Or9g</td><td>Sequence8Os9g</td><td>Sequence74Ou9g</td><td>Sequence148Op9g</td><td>Sequence121Oa9g</td></tr>
<tr><td>4</td><td>Sequence93Or9g</td><td>Sequence9Os9g</td><td>Sequence75Ou9g</td><td>Sequence149Op9g</td><td>Sequence122Oa9g</td></tr>
<tr><td>4</td><td>Sequence94Or9g</td><td>||</td><td>Sequence76Ou9g</td><td>||</td><td>||</td></tr>
<tr><td>4</td><td>Sequence95Or9g</td><td>||</td><td>||</td><td>||</td><td>||</td></tr>
<tr><td>4</td><td>Sequence96Or9g</td><td>Sequence10Os9g</td><td>||</td><td>Sequence150Op9g</td><td>Sequence124Oa9g</td></tr>
<tr><td>4</td><td>Sequence97Or9g</td><td>Sequence12Os9g</td><td>||</td><td>||</td><td>||</td></tr>
<tr><td>4</td><td>Sequence98Or9g</td><td>Sequence13Os9g</td><td>||</td><td>||</td><td>||</td></tr>
<tr><td>4</td><td>Sequence99Or9g</td><td>Sequence15Os9g</td><td>Sequence79Ou9g</td><td>Sequence151Op9g</td><td>Sequence125Oa9g</td></tr>
<tr><td>4</td><td>Sequence100Or9g</td><td>Sequence17Os9g</td><td>Sequence80Ou9g</td><td>Sequence152Op9g</td><td>Sequence126Oa9g</td></tr>
<tr><td>4</td><td>Sequence101Or9g</td><td>Sequence18Os9g</td><td>Sequence81Ou9g</td><td>||</td><td>||</td></tr>
<tr><td>4</td><td>Sequence102Or9g</td><td>Sequence19Os9g</td><td>Sequence82Ou9g</td><td>Sequence154Op9g</td><td>Sequence129Oa9g</td></tr>
<tr><td>4</td><td>Sequence103Or9g</td><td>Sequence20Os9g</td><td>Sequence83Ou9g</td><td>Sequence155Op9g</td><td>Sequence130Oa9g</td></tr>
<tr><td>4</td><td>Sequence104Or9g</td><td>Sequence21Os9g</td><td>Sequence84Ou9g</td><td>Sequence156Op9g</td><td>Sequence131Oa9g</td></tr>
<tr><td>4</td><td>Sequence105Or9g</td><td>Sequence22Os9g</td><td>Sequence85Ou9g</td><td>Sequence157Op9g</td><td>Sequence132Oa9g</td></tr>
<tr><td>2</td><td>Sequence106Or9g</td><td>Sequence23Os9g</td><td></td><td></td><td>Sequence133Oa9g</td></tr>
<tr><td>0</td><td>Sequence107Or9g</td><td></td><td></td><td></td><td></td></tr>
<tr><td>0</td><td>Sequence108Or9g</td><td></td><td></td><td></td><td></td></tr>
</table>

Figure S12: Synteny block diagram for *MIR172*A with *Oryza rufipogon* as reference. The first column shows duplication depth at each gene locus, second column shows the genes in reference chromosomes and the following is aligned collinear blocks where only match genes are displayed. The alignment among non-anchor genes is discarded in the output and denoted by '||' in the multi-alignment of gene orders. Os9- *Oryza sativa* chr 9, Ou9- *Oryza glumaepatula* chr 9, Or9- *Oryza rufipogon* chr 9, Oa9- *Oryza brachyantha* chr 9, Op9- *Oryza punctata* chr 9.

cells

MDPI

<table>
<tr><th>Duplication depth</th><th>Reference chromosome</th><th>Collinear blocks</th><th></th><th></th><th></th></tr>
<tr><td>1</td><td>Sequence109Oa9g</td><td>Sequence136Op9g</td><td></td><td></td><td></td></tr>
<tr><td>1</td><td>Sequence110Oa9g</td><td>Sequence137Op9g</td><td></td><td></td><td></td></tr>
<tr><td>1</td><td>Sequence111Oa9g</td><td>Sequence138Op9g</td><td></td><td></td><td></td></tr>
<tr><td>1</td><td>Sequence112Oa9g</td><td>Sequence139Op9g</td><td></td><td></td><td></td></tr>
<tr><td>1</td><td>Sequence113Oa9g</td><td>||</td><td></td><td></td><td></td></tr>
<tr><td>1</td><td>Sequence114Oa9g</td><td>Sequence141Op9g</td><td></td><td></td><td></td></tr>
<tr><td>1</td><td>Sequence115Oa9g</td><td>Sequence142Op9g</td><td></td><td></td><td></td></tr>
<tr><td>2</td><td>Sequence116Oa9g</td><td>Sequence143Op9g</td><td>Sequence1Os9g</td><td></td><td></td></tr>
<tr><td>2</td><td>Sequence117Oa9g</td><td>Sequence145Op9g</td><td>Sequence3Os9g</td><td></td><td></td></tr>
<tr><td>2</td><td>Sequence118Oa9g</td><td>||</td><td>||</td><td></td><td></td></tr>
<tr><td>4</td><td>Sequence119Oa9g</td><td>||</td><td>||</td><td>Sequence91Or9g</td><td>Sequence73Ou9g</td></tr>
<tr><td>4</td><td>Sequence120Oa9g</td><td>||</td><td>||</td><td>||</td><td>||</td></tr>
<tr><td>4</td><td>Sequence121Oa9g</td><td>Sequence148Op9g</td><td>Sequence8Os9g</td><td>Sequence92Or9g</td><td>Sequence74Ou9g</td></tr>
<tr><td>4</td><td>Sequence122Oa9g</td><td>Sequence149Op9g</td><td>Sequence9Os9g</td><td>Sequence93Or9g</td><td>Sequence75Ou9g</td></tr>
<tr><td>4</td><td>Sequence123Oa9g</td><td>||</td><td>||</td><td>||</td><td>||</td></tr>
<tr><td>4</td><td>Sequence124Oa9g</td><td>Sequence150Op9g</td><td>Sequence10Os9g</td><td>Sequence96Or9g</td><td>Sequence76Ou9g</td></tr>
<tr><td>4</td><td>Sequence125Oa9g</td><td>Sequence151Op9g</td><td>Sequence15Os9g</td><td>Sequence99Or9g</td><td>Sequence79Ou9g</td></tr>
<tr><td>4</td><td>Sequence126Oa9g</td><td>Sequence152Op9g</td><td>Sequence17Os9g</td><td>Sequence100Or9g</td><td>Sequence80Ou9g</td></tr>
<tr><td>4</td><td>Sequence127Oa9g</td><td>||</td><td>||</td><td>||</td><td>||</td></tr>
<tr><td>4</td><td>Sequence128Oa9g</td><td>||</td><td>||</td><td>||</td><td>||</td></tr>
<tr><td>4</td><td>Sequence129Oa9g</td><td>Sequence154Op9g</td><td>Sequence19Os9g</td><td>Sequence102Or9g</td><td>Sequence82Ou9g</td></tr>
<tr><td>4</td><td>Sequence130Oa9g</td><td>Sequence155Op9g</td><td>Sequence20Os9g</td><td>Sequence103Or9g</td><td>Sequence83Ou9g</td></tr>
<tr><td>4</td><td>Sequence131Oa9g</td><td>Sequence156Op9g</td><td>Sequence21Os9g</td><td>Sequence104Or9g</td><td>Sequence84Ou9g</td></tr>
<tr><td>4</td><td>Sequence132Oa9g</td><td>Sequence157Op9g</td><td>Sequence22Os9g</td><td>Sequence105Or9g</td><td>Sequence85Ou9g</td></tr>
<tr><td>2</td><td>Sequence133Oa9g</td><td></td><td>Sequence23Os9g</td><td>Sequence106Or9g</td><td></td></tr>
<tr><td>0</td><td>Sequence134Oa9g</td><td></td><td></td><td></td><td></td></tr>
<tr><td>0</td><td>Sequence135Oa9g</td><td></td><td></td><td></td><td></td></tr>
</table>

Figure S13: Synteny block diagram for *MIR172*A with *Oryza brachyantha* as reference. The first column shows duplication depth at each gene locus, second column shows the genes in reference chromosomes and the following is aligned collinear blocks where only match genes are displayed. The alignment among non-anchor genes is discarded in the output and denoted by '||' in the multi-alignment of gene orders. Os9- *Oryza sativa* chr 9, Ou9- *Oryza glumaepatula* chr 9, Or9- *Oryza rufipogon* chr 9, Oa9- *Oryza brachyantha* chr 9, Op9- *Oryza punctata* chr 9.

Duplication depth	Reference chromosome	Collinear blocks			
1	Sequence136Op9g	Sequence109Oa9g			
1	Sequence137Op9g	Sequence110Oa9g			
1	Sequence138Op9g	Sequence111Oa9g			
1	Sequence139Op9g	Sequence112Oa9g			
1	Sequence140Op9g	||			
1	Sequence141Op9g	Sequence114Oa9g			
1	Sequence142Op9g	Sequence115Oa9g			
2	Sequence143Op9g	Sequence116Oa9g	Sequence1Os9g		
2	Sequence144Op9g	||	||		
2	Sequence145Op9g	Sequence117Oa9g	Sequence3Os9g		
2	Sequence146Op9g	||	||		
4	Sequence147Op9g	||	||	Sequence73Ou9g	Sequence91Or9g
4	Sequence148Op9g	Sequence121Oa9g	Sequence8Os9g	Sequence74Ou9g	Sequence92Or9g
4	Sequence149Op9g	Sequence122Oa9g	Sequence9Os9g	Sequence75Ou9g	Sequence93Or9g
4	Sequence150Op9g	Sequence124Oa9g	Sequence10Os9g	Sequence76Ou9g	Sequence96Or9g
4	Sequence151Op9g	Sequence125Oa9g	Sequence15Os9g	Sequence79Ou9g	Sequence99Or9g
4	Sequence152Op9g	Sequence126Oa9g	Sequence17Os9g	Sequence80Ou9g	Sequence100Or9g
4	Sequence153Op9g	||	||	||	||
4	Sequence154Op9g	Sequence129Oa9g	Sequence19Os9g	Sequence82Ou9g	Sequence102Or9g
4	Sequence155Op9g	Sequence130Oa9g	Sequence20Os9g	Sequence83Ou9g	Sequence103Or9g
4	Sequence156Op9g	Sequence131Oa9g	Sequence21Os9g	Sequence84Ou9g	Sequence104Or9g
4	Sequence157Op9g	Sequence132Oa9g	Sequence22Os9g	Sequence85Ou9g	Sequence105Or9g

Figure S14: Synteny block diagram for *MIR172*A with *Oryza punctata* as reference. The first column shows duplication depth at each gene locus, second column shows the genes in reference chromosomes and the following is aligned collinear blocks where only match genes are displayed. The alignment among non-anchor genes is discarded in the output and denoted by '||' in the multi-alignment of gene orders. Os9- *Oryza sativa* chr 9, Ou9- *Oryza glumaepatula* chr 9, Or9- *Oryza rufipogon* chr 9, Oa9- *Oryza brachyantha* chr 9, Op9- *Oryza punctata* chr 9.

Duplication depth	Reference chromosome	Collinear blocks
0	Sequence158Sb9g	
0	Sequence159Sb9g	
0	Sequence160Sb9g	
0	Sequence161Sb9g	
0	Sequence162Sb9g	
0	Sequence163Sb9g	
0	Sequence164Sb9g	
0	Sequence165Sb9g	
0	Sequence166Sb9g	
0	Sequence167Sb9g	
0	Sequence168Sb9g	
0	Sequence169Sb9g	
0	Sequence170Sb9g	
0	Sequence171Sb9g	
0	Sequence172Sb9g	
0	Sequence173Sb9g	
0	Sequence174Sb9g	
0	Sequence175Sb9g	
0	Sequence176Sb9g	
0	Sequence177Sb9g	

Figure S15: Synteny block diagram for *MIR172*A with *Sorghum bicolor* as reference. The first column shows duplication depth at each gene locus, second column shows the genes in reference chromosomes and the following is aligned collinear blocks where only match genes are displayed. The alignment among non-anchor genes is discarded in the output and denoted by '||' in the multi-alignment of gene orders. Sb9- *Sorghum bicolor* chr 9.

Duplication depth	Reference chromosome	Collinear blocks
0	Sequence178Zm7g	
0	Sequence179Zm7g	
0	Sequence180Zm7g	
0	Sequence181Zm7g	
0	Sequence182Zm7g	
0	Sequence183Zm7g	
0	Sequence184Zm7g	
0	Sequence185Zm7g	
0	Sequence186Zm7g	
0	Sequence187Zm7g	
0	Sequence188Zm7g	
0	Sequence189Zm7g	
0	Sequence190Zm7g	
0	Sequence191Zm7g	

Figure S16: Synteny block diagram for *MIR172*A with *Zea mays* as reference. The first column shows duplication depth at each gene locus, second column shows the genes in reference chromosomes and the following is aligned collinear blocks where only match genes are displayed. The alignment among non-anchor genes is discarded in the output and denoted by '||' in the multi-alignment of gene orders. Zm7-*Zea mays* chr 7.

cells

Duplication depth	Reference chromosome	Collinear blocks
0	Sequence192At2g	
0	Sequence193At2g	
0	Sequence194At2g	
0	Sequence195At2g	
0	Sequence196At2g	
0	Sequence197At2g	
0	Sequence198At2g	
0	Sequence199At2g	
0	Sequence200At2g	
0	Sequence201At2g	
0	Sequence202At2g	
0	Sequence203At2g	
0	Sequence204At2g	
0	Sequence205At2g	
0	Sequence206At2g	
0	Sequence207At2g	
0	Sequence208At2g	
0	Sequence209At2g	
0	Sequence210At2g	
0	Sequence211At2g	
0	Sequence212At2g	
0	Sequence213At2g	
0	Sequence214At2g	
0	Sequence215At2g	
0	Sequence216At2g	
0	Sequence217At2g	
0	Sequence218At2g	

Figure S17: Synteny block diagram for *MIR172*A with *Arabidopsis thaliana* as reference. The first column shows duplication depth at each gene locus, second column shows the genes in reference chromosomes and the following is aligned collinear blocks where only match genes are displayed. The alignment among non-anchor genes is discarded in the output and denoted by '||' in the multi-alignment of gene orders. At2- *Arabidopsis thaliana* chr 2.

cells

<table>
<tr><th>Duplication depth</th><th>Reference chromosome</th><th colspan="5">Collinear blocks</th></tr>
<tr><td>2</td><td>Sequence24Ob1g</td><td>Sequence45Og1g</td><td>Sequence107Op1g</td><td></td><td></td><td></td></tr>
<tr><td>3</td><td>Sequence25Ob1g</td><td>Sequence46Og1g</td><td>||</td><td>Sequence81Or1g</td><td></td><td></td></tr>
<tr><td>4</td><td>Sequence26Ob1g</td><td>Sequence47Og1g</td><td>Sequence109Op1g</td><td>Sequence82Or1g</td><td>Sequence1Os1g</td><td></td></tr>
<tr><td>4</td><td>Sequence27Ob1g</td><td>||</td><td>||</td><td>||</td><td>||</td><td></td></tr>
<tr><td>4</td><td>Sequence28Ob1g</td><td>Sequence48Og1g</td><td>Sequence112Op1g</td><td>Sequence83Or1g</td><td>Sequence2Os1g</td><td></td></tr>
<tr><td>5</td><td>Sequence29Ob1g</td><td>Sequence49Og1g</td><td>||</td><td>Sequence84Or1g</td><td>Sequence5Os1g</td><td>Sequence63Ou1g</td></tr>
<tr><td>5</td><td>Sequence30Ob1g</td><td>Sequence50Og1g</td><td>Sequence115Op1g</td><td>Sequence85Or1g</td><td>Sequence6Os1g</td><td>Sequence64Ou1g</td></tr>
<tr><td>5</td><td>Sequence31Ob1g</td><td>||</td><td>||</td><td>||</td><td>Sequence7Os1g</td><td>Sequence65Ou1g</td></tr>
<tr><td>5</td><td>Sequence32Ob1g</td><td>Sequence52Og1g</td><td>Sequence117Op1g</td><td>Sequence87Or1g</td><td>||</td><td>Sequence66Ou1g</td></tr>
<tr><td>5</td><td>Sequence33Ob1g</td><td>||</td><td>||</td><td>||</td><td>Sequence9Os1g</td><td>||</td></tr>
<tr><td>5</td><td>Sequence34Ob1g</td><td>||</td><td>||</td><td>Sequence88Or1g</td><td>Sequence10Os1g</td><td>||</td></tr>
<tr><td>5</td><td>Sequence35Ob1g</td><td>Sequence54Og1g</td><td>Sequence118Op1g</td><td>Sequence89Or1g</td><td>Sequence14Os1g</td><td>Sequence70Ou1g</td></tr>
<tr><td>5</td><td>Sequence36Ob1g</td><td>Sequence55Og1g</td><td>||</td><td>Sequence90Or1g</td><td>Sequence15Os1g</td><td>Sequence71Ou1g</td></tr>
<tr><td>5</td><td>Sequence37Ob1g</td><td>||</td><td>||</td><td>||</td><td>||</td><td>||</td></tr>
<tr><td>5</td><td>Sequence38Ob1g</td><td>||</td><td>||</td><td>||</td><td>||</td><td>||</td></tr>
<tr><td>5</td><td>Sequence39Ob1g</td><td>||</td><td>||</td><td>||</td><td>Sequence17Os1g</td><td>Sequence74Ou1g</td></tr>
<tr><td>5</td><td>Sequence40Ob1g</td><td>Sequence57Og1g</td><td>Sequence124Op1g</td><td>Sequence93Or1g</td><td>Sequence18Os1g</td><td>Sequence75Ou1g</td></tr>
<tr><td>5</td><td>Sequence41Ob1g</td><td>Sequence58Og1g</td><td>Sequence125Op1g</td><td>Sequence94Or1g</td><td>||</td><td>Sequence76Ou1g</td></tr>
<tr><td>5</td><td>Sequence42Ob1g</td><td>Sequence59Og1g</td><td>Sequence129Op1g</td><td>Sequence95Or1g</td><td>Sequence21Os1g</td><td>Sequence78Ou1g</td></tr>
<tr><td>1</td><td>Sequence43Ob1g</td><td>Sequence60Og1g</td><td></td><td></td><td></td><td></td></tr>
<tr><td>0</td><td>Sequence44Ob1g</td><td></td><td></td><td></td><td></td><td></td></tr>
</table>

Figure S18: Synteny block diagram for *MIR172B* with *Oryza barthii* as reference. The first column shows duplication depth at each gene locus, second column shows the genes in reference chromosomes and the following is aligned collinear blocks where only match genes are displayed. The alignment among non-anchor genes is discarded in the output and denoted by '||' in the multi-alignment of gene orders. Os1- *Oryza sativa* chr 1, Ob1- *Oryza barthii* chr 1, Og1- *Oryza glaberrima* chr 1, Ou1- *Oryza glumaepatula* chr 1, Or1- *Oryza rufipogon* chr 1, Op1- *Oryza punctata* chr 1.

<table>
<tr><th>Duplication depth</th><th>Reference chromosome</th><th>Collinear blocks</th><th></th><th></th><th></th><th></th></tr>
<tr><td>2</td><td>Sequence45Og1g</td><td>Sequence107Op1g</td><td>Sequence24Ob1g</td><td></td><td></td><td></td></tr>
<tr><td>3</td><td>Sequence46Og1g</td><td>||</td><td>Sequence25Ob1g</td><td>Sequence81Or1g</td><td></td><td></td></tr>
<tr><td>4</td><td>Sequence47Og1g</td><td>Sequence109Op1g</td><td>Sequence26Ob1g</td><td>Sequence82Or1g</td><td>Sequence1Os1g</td><td></td></tr>
<tr><td>4</td><td>Sequence48Og1g</td><td>Sequence112Op1g</td><td>Sequence28Ob1g</td><td>Sequence83Or1g</td><td>Sequence2Os1g</td><td></td></tr>
<tr><td>5</td><td>Sequence49Og1g</td><td>Sequence113Op1g</td><td>Sequence29Ob1g</td><td>Sequence84Or1g</td><td>Sequence5Os1g</td><td>Sequence63Ou1g</td></tr>
<tr><td>5</td><td>Sequence50Og1g</td><td>Sequence115Op1g</td><td>Sequence30Ob1g</td><td>Sequence85Or1g</td><td>Sequence6Os1g</td><td>Sequence64Ou1g</td></tr>
<tr><td>5</td><td>Sequence51Og1g</td><td>||</td><td>||</td><td>||</td><td>||</td><td>||</td></tr>
<tr><td>5</td><td>Sequence52Og1g</td><td>Sequence117Op1g</td><td>Sequence32Ob1g</td><td>Sequence87Or1g</td><td>||</td><td>Sequence66Ou1g</td></tr>
<tr><td>5</td><td>Sequence53Og1g</td><td>||</td><td>||</td><td>||</td><td>Sequence11Os1g</td><td>Sequence67Ou1g</td></tr>
<tr><td>5</td><td>Sequence54Og1g</td><td>Sequence118Op1g</td><td>Sequence35Ob1g</td><td>Sequence89Or1g</td><td>Sequence14Os1g</td><td>Sequence70Ou1g</td></tr>
<tr><td>5</td><td>Sequence55Og1g</td><td>Sequence119Op1g</td><td>Sequence36Ob1g</td><td>Sequence90Or1g</td><td>Sequence15Os1g</td><td>Sequence71Ou1g</td></tr>
<tr><td>5</td><td>Sequence56Og1g</td><td>||</td><td>||</td><td>||</td><td>||</td><td>||</td></tr>
<tr><td>5</td><td>Sequence57Og1g</td><td>Sequence124Op1g</td><td>Sequence40Ob1g</td><td>Sequence93Or1g</td><td>Sequence18Os1g</td><td>Sequence75Ou1g</td></tr>
<tr><td>5</td><td>Sequence58Og1g</td><td>Sequence125Op1g</td><td>Sequence41Ob1g</td><td>Sequence94Or1g</td><td>Sequence19Os1g</td><td>Sequence76Ou1g</td></tr>
<tr><td>5</td><td>Sequence59Og1g</td><td>Sequence129Op1g</td><td>Sequence42Ob1g</td><td>Sequence95Or1g</td><td>Sequence21Os1g</td><td>Sequence78Ou1g</td></tr>
<tr><td>1</td><td>Sequence60Og1g</td><td></td><td>Sequence43Ob1g</td><td></td><td></td><td></td></tr>
<tr><td>0</td><td>Sequence61Og1g</td><td></td><td></td><td></td><td></td><td></td></tr>
<tr><td>0</td><td>Sequence62Og1g</td><td></td><td></td><td></td><td></td><td></td></tr>
</table>

Figure S19: Synteny block diagram for *MIR172B* with *Oryza glaberrima* as reference. The first column shows duplication depth at each gene locus, second column shows the genes in reference chromosomes and the following is aligned collinear blocks where only match genes are displayed. The alignment among non-anchor genes is discarded in the output and denoted by '||' in the multi-alignment of gene orders. Os1- *Oryza sativa* chr 1, Ob1- *Oryza barthii* chr 1, Og1- *Oryza glaberrima* chr 1, Ou1- *Oryza glumaepatula* chr 1, Or1- *Oryza rufipogon* chr 1, Op1- *Oryza punctata* chr 1.

Duplication depth	Reference chromosome	Collinear blocks				
5	Sequence63Ou1g	Sequence49Og1g	Sequence113Op1g	Sequence29Ob1g	Sequence84Or1g	Sequence5Os1g
5	Sequence64Ou1g	Sequence50Og1g	Sequence115Op1g	Sequence30Ob1g	Sequence85Or1g	Sequence6Os1g
5	Sequence65Ou1g	||	||	Sequence31Ob1g	||	Sequence7Os1g
5	Sequence66Ou1g	Sequence52Og1g	Sequence117Op1g	Sequence32Ob1g	Sequence87Or1g	||
5	Sequence67Ou1g	Sequence53Og1g	||	||	||	Sequence11Os1g
5	Sequence68Ou1g	||	||	||	||	||
5	Sequence69Ou1g	||	||	||	||	||
5	Sequence70Ou1g	Sequence54Og1g	Sequence118Op1g	Sequence35Ob1g	Sequence89Or1g	Sequence14Os1g
5	Sequence71Ou1g	Sequence55Og1g	Sequence119Op1g	Sequence36Ob1g	Sequence90Or1g	Sequence15Os1g
5	Sequence72Ou1g	||	||	||	||	||
5	Sequence73Ou1g	||	||	||	||	||
5	Sequence74Ou1g	||	||	Sequence39Ob1g	||	Sequence17Os1g
5	Sequence75Ou1g	Sequence57Og1g	Sequence124Op1g	Sequence40Ob1g	Sequence93Or1g	Sequence18Os1g
5	Sequence76Ou1g	Sequence58Og1g	Sequence125Op1g	Sequence41Ob1g	Sequence94Or1g	Sequence19Os1g
5	Sequence77Ou1g	||	||	||	||	||
5	Sequence78Ou1g	Sequence59Og1g	Sequence129Op1g	Sequence42Ob1g	Sequence95Or1g	Sequence21Os1g
2	Sequence79Ou1g		||		Sequence96Or1g	
2	Sequence80Ou1g		Sequence131Op1g		Sequence97Or1g	

Figure S20: Synteny block diagram for *MIR172B* with *Oryza glumaepatula* as reference. The first column shows duplication depth at each gene locus, second column shows the genes in reference chromosomes and the following is aligned collinear blocks where only match genes are displayed. The alignment among non-anchor genes is discarded in the output and denoted by '||' in the multi-alignment of gene orders. Os1- *Oryza sativa* chr 1, Ob1- *Oryza barthii* chr 1, Og1- *Oryza glaberrima* chr 1, Ou1- *Oryza glumaepatula* chr 1, Or1- *Oryza rufipogon* chr 1, Op1- *Oryza punctata* chr 1.

cells

MDPI

<table>
<tr><th>Duplication depth</th><th>Reference chromosome</th><th>Collinear blocks</th><th></th><th></th><th></th><th></th></tr>
<tr><td>2</td><td>Sequence81Or1g</td><td>Sequence25Ob1g</td><td>Sequence46Og1g</td><td></td><td></td><td></td></tr>
<tr><td>4</td><td>Sequence82Or1g</td><td>Sequence26Ob1g</td><td>Sequence47Og1g</td><td>Sequence1Os1g</td><td>Sequence109Op1g</td><td></td></tr>
<tr><td>4</td><td>Sequence83Or1g</td><td>Sequence28Ob1g</td><td>Sequence48Og1g</td><td>Sequence2Os1g</td><td>Sequence112Op1g</td><td></td></tr>
<tr><td>5</td><td>Sequence84Or1g</td><td>Sequence29Ob1g</td><td>Sequence49Og1g</td><td>Sequence5Os1g</td><td>Sequence113Op1g</td><td>Sequence63Ou1g</td></tr>
<tr><td>5</td><td>Sequence85Or1g</td><td>Sequence30Ob1g</td><td>Sequence50Og1g</td><td>Sequence6Os1g</td><td>Sequence116Op1g</td><td>Sequence64Ou1g</td></tr>
<tr><td>5</td><td>Sequence86Or1g</td><td>||</td><td>||</td><td>||</td><td>||</td><td>||</td></tr>
<tr><td>5</td><td>Sequence87Or1g</td><td>Sequence32Ob1g</td><td>Sequence52Og1g</td><td>Sequence8Os1g</td><td>||</td><td>Sequence66Ou1g</td></tr>
<tr><td>5</td><td>Sequence88Or1g</td><td>Sequence34Ob1g</td><td>||</td><td>||</td><td>||</td><td>||</td></tr>
<tr><td>5</td><td>Sequence89Or1g</td><td>Sequence35Ob1g</td><td>Sequence54Og1g</td><td>Sequence14Os1g</td><td>||</td><td>Sequence70Ou1g</td></tr>
<tr><td>5</td><td>Sequence90Or1g</td><td>Sequence36Ob1g</td><td>Sequence55Og1g</td><td>Sequence15Os1g</td><td>Sequence119Op1g</td><td>Sequence71Ou1g</td></tr>
<tr><td>5</td><td>Sequence91Or1g</td><td>||</td><td>||</td><td>||</td><td>||</td><td>||</td></tr>
<tr><td>5</td><td>Sequence92Or1g</td><td>||</td><td>||</td><td>||</td><td>||</td><td>||</td></tr>
<tr><td>5</td><td>Sequence93Or1g</td><td>Sequence40Ob1g</td><td>Sequence57Og1g</td><td>Sequence18Os1g</td><td>Sequence124Op1g</td><td>Sequence75Ou1g</td></tr>
<tr><td>5</td><td>Sequence94Or1g</td><td>Sequence41Ob1g</td><td>Sequence58Og1g</td><td>Sequence19Os1g</td><td>Sequence125Op1g</td><td>Sequence76Ou1g</td></tr>
<tr><td>5</td><td>Sequence95Or1g</td><td>Sequence42Ob1g</td><td>Sequence59Og1g</td><td>Sequence21Os1g</td><td>||</td><td>Sequence78Ou1g</td></tr>
<tr><td>2</td><td>Sequence96Or1g</td><td></td><td></td><td></td><td>Sequence127Op1g</td><td>Sequence79Ou1g</td></tr>
<tr><td>2</td><td>Sequence97Or1g</td><td></td><td></td><td></td><td>Sequence131Op1g</td><td>Sequence80Ou1g</td></tr>
</table>

Figure S21: Synteny block diagram for *MIR172B* with *Oryza rufipogon* as reference. The first column shows duplication depth at each gene locus, second column shows the genes in reference chromosomes and the following is aligned collinear blocks where only match genes are displayed. The alignment among non-anchor genes is discarded in the output and denoted by '||' in the multi-alignment of gene orders. Os1- *Oryza sativa* chr 1, Ob1- *Oryza barthii* chr 1, Og1- *Oryza glaberrima* chr 1, Ou1- *Oryza glumaepatula* chr 1, Or1- *Oryza rufipogon* chr 1, Op1- *Oryza punctata* chr 1.

Duplication depth	Reference chromosome	Collinear blocks
0	Sequence98Oa1g	
0	Sequence99Oa1g	
0	Sequence100Oa1g	
0	Sequence101Oa1g	
0	Sequence102Oa1g	
0	Sequence103Oa1g	
0	Sequence104Oa1g	
0	Sequence105Oa1g	
0	Sequence106Oa1g	

Figure S22: Synteny block diagram for *MIR172B* with *Oryza brachyantha* as reference. The first column shows duplication depth at each gene locus, second column shows the genes in reference chromosomes and the following is aligned collinear blocks where only match genes are displayed. The alignment among non-anchor genes is discarded in the output and denoted by '||' in the multi-alignment of gene orders. Oa1- *Oryza brachyantha* chr 1.

cells MDPI

<table>
<tr><th>Duplication depth</th><th>Reference chromosome</th><th colspan="5">Collinear blocks</th></tr>
<tr><td>2</td><td>Sequence107Op1g</td><td>Sequence24Ob1g</td><td>Sequence45Og1g</td><td></td><td></td><td></td></tr>
<tr><td>2</td><td>Sequence108Op1g</td><td>||</td><td>||</td><td></td><td></td><td></td></tr>
<tr><td>3</td><td>Sequence109Op1g</td><td>Sequence26Ob1g</td><td>Sequence47Og1g</td><td>Sequence82Or1g</td><td></td><td></td></tr>
<tr><td>3</td><td>Sequence110Op1g</td><td>||</td><td>||</td><td>||</td><td></td><td></td></tr>
<tr><td>4</td><td>Sequence111Op1g</td><td>||</td><td>||</td><td>||</td><td>Sequence1Os1g</td><td></td></tr>
<tr><td>4</td><td>Sequence112Op1g</td><td>Sequence28Ob1g</td><td>Sequence48Og1g</td><td>Sequence83Or1g</td><td>Sequence2Os1g</td><td></td></tr>
<tr><td>5</td><td>Sequence113Op1g</td><td>||</td><td>Sequence49Og1g</td><td>Sequence84Or1g</td><td>Sequence5Os1g</td><td>Sequence63Ou1g</td></tr>
<tr><td>5</td><td>Sequence114Op1g</td><td>||</td><td>||</td><td>||</td><td>||</td><td>||</td></tr>
<tr><td>5</td><td>Sequence115Op1g</td><td>Sequence30Ob1g</td><td>Sequence50Og1g</td><td>||</td><td>Sequence6Os1g</td><td>Sequence64Ou1g</td></tr>
<tr><td>5</td><td>Sequence116Op1g</td><td>||</td><td>||</td><td>Sequence85Or1g</td><td>||</td><td>||</td></tr>
<tr><td>5</td><td>Sequence117Op1g</td><td>Sequence32Ob1g</td><td>Sequence52Og1g</td><td>||</td><td>Sequence8Os1g</td><td>Sequence66Ou1g</td></tr>
<tr><td>5</td><td>Sequence118Op1g</td><td>Sequence35Ob1g</td><td>Sequence54Og1g</td><td>||</td><td>Sequence14Os1g</td><td>Sequence70Ou1g</td></tr>
<tr><td>5</td><td>Sequence119Op1g</td><td>||</td><td>Sequence55Og1g</td><td>Sequence90Or1g</td><td>Sequence15Os1g</td><td>Sequence71Ou1g</td></tr>
<tr><td>5</td><td>Sequence120Op1g</td><td>||</td><td>||</td><td>||</td><td>||</td><td>||</td></tr>
<tr><td>5</td><td>Sequence121Op1g</td><td>||</td><td>||</td><td>||</td><td>||</td><td>||</td></tr>
<tr><td>5</td><td>Sequence122Op1g</td><td>||</td><td>||</td><td>||</td><td>||</td><td>||</td></tr>
<tr><td>5</td><td>Sequence123Op1g</td><td>||</td><td>||</td><td>||</td><td>||</td><td>||</td></tr>
<tr><td>5</td><td>Sequence124Op1g</td><td>Sequence40Ob1g</td><td>Sequence57Og1g</td><td>Sequence93Or1g</td><td>||</td><td>Sequence75Ou1g</td></tr>
<tr><td>5</td><td>Sequence125Op1g</td><td>Sequence41Ob1g</td><td>Sequence58Og1g</td><td>Sequence94Or1g</td><td>Sequence19Os1g</td><td>Sequence76Ou1g</td></tr>
<tr><td>5</td><td>Sequence126Op1g</td><td>||</td><td>||</td><td>||</td><td>||</td><td>||</td></tr>
<tr><td>5</td><td>Sequence127Op1g</td><td>||</td><td>||</td><td>Sequence96Or1g</td><td>||</td><td>||</td></tr>
<tr><td>5</td><td>Sequence128Op1g</td><td>||</td><td>||</td><td>||</td><td>||</td><td>||</td></tr>
<tr><td>5</td><td>Sequence129Op1g</td><td>Sequence42Ob1g</td><td>Sequence59Og1g</td><td>||</td><td>Sequence21Os1g</td><td>Sequence78Ou1g</td></tr>
<tr><td>2</td><td>Sequence130Op1g</td><td></td><td></td><td>||</td><td></td><td>||</td></tr>
<tr><td>2</td><td>Sequence131Op1g</td><td></td><td></td><td>Sequence97Or1g</td><td></td><td>Sequence80Ou1g</td></tr>
</table>

Figure S23: Synteny block diagram for *MIR172B* with *Oryza punctata* as reference. The first column shows duplication depth at each gene locus, second column shows the genes in reference chromosomes and the following is aligned collinear blocks where only match genes are displayed. The alignment among non-anchor genes is discarded in the output and denoted by '||' in the multi-alignment of gene orders. Os1- *Oryza sativa* chr 1, Ob1- *Oryza barthii* chr 1, Og1- *Oryza glaberrima* chr 1, Ou1- *Oryza glumaepatula* chr 1, Or1- *Oryza rufipogon* chr 1, Op1- *Oryza punctata* chr 1.

Duplication depth	Reference chromosome	Collinear blocks
0	Sequence132Sb3g	
0	Sequence133Sb3g	
0	Sequence134Sb3g	
0	Sequence135Sb3g	
0	Sequence136Sb3g	
0	Sequence137Sb3g	
0	Sequence138Sb3g	
0	Sequence139Sb3g	
0	Sequence140Sb3g	
0	Sequence141Sb3g	
0	Sequence142Sb3g	
0	Sequence143Sb3g	
0	Sequence144Sb3g	
0	Sequence145Sb3g	
0	Sequence146Sb3g	
0	Sequence147Sb3g	
0	Sequence148Sb3g	
0	Sequence149Sb3g	
0	Sequence150Sb3g	
0	Sequence151Sb3g	
0	Sequence152Sb3g	

Figure S24: Synteny block diagram for *MIR172B* with *Sorghum bicolor* as reference. The first column shows duplication depth at each gene locus, second column shows the genes in reference chromosomes and the following is aligned collinear blocks where only match genes are displayed. The alignment among non-anchor genes is discarded in the output and denoted by '||' in the multi-alignment of gene orders. Sb3 - *Sorghum bicolor* chr 3.

Duplication depth	Reference chromosome	Collinear blocks
0	Sequence153Zm5g	
0	Sequence154Zm5g	
0	Sequence155Zm5g	
0	Sequence156Zm5g	
0	Sequence157Zm5g	
0	Sequence158Zm5g	
0	Sequence159Zm5g	
0	Sequence160Zm5g	
0	Sequence161Zm5g	
0	Sequence162Zm5g	
0	Sequence163Zm5g	
0	Sequence164Zm5g	
0	Sequence165Zm5g	
0	Sequence166Zm5g	
0	Sequence167Zm5g	

Figure S25: Synteny block diagram for *MIR172B* with *Zea mays* as reference. The first column shows duplication depth at each gene locus, second column shows the genes in reference chromosomes and the following is aligned collinear blocks where only match genes are displayed. The alignment among non-anchor genes is discarded in the output and denoted by '||' in the multi-alignment of gene orders. Zm5 - *Zea mays* chr 5.

Duplication depth	Reference chromosome	Collinear blocks
0	Sequence168At5g	
0	Sequence169At5g	
0	Sequence170At5g	
0	Sequence171At5g	
0	Sequence172At5g	
0	Sequence173At5g	
0	Sequence174At5g	
0	Sequence175At5g	
0	Sequence176At5g	
0	Sequence177At5g	
0	Sequence178At5g	
0	Sequence179At5g	
0	Sequence180At5g	
0	Sequence181At5g	
0	Sequence182At5g	
0	Sequence183At5g	
0	Sequence184At5g	
0	Sequence185At5g	
0	Sequence186At5g	
0	Sequence187At5g	
0	Sequence188At5g	
0	Sequence189At5g	
0	Sequence190At5g	
0	Sequence191At5g	
0	Sequence192At5g	
0	Sequence193At5g	
0	Sequence194At5g	
0	Sequence195At5g	

Figure S26: Synteny block diagram for *MIR172B* with *Arabidopsis thaliana* as reference. The first column shows duplication depth at each gene locus, second column shows the genes in reference chromosomes and the following is aligned collinear blocks where only match genes are displayed. The alignment among non-anchor genes is discarded in the output and denoted by '||' in the multi-alignment of gene orders. At5- *Arabidopsis thaliana* chr 5.

<table>
<tr><th>Duplication depth</th><th>Reference chromosome</th><th>Collinear blocks</th><th></th><th></th><th></th></tr>
<tr><td>0</td><td>Sequence22Ob7g</td><td></td><td></td><td></td><td></td></tr>
<tr><td>0</td><td>Sequence23Ob7g</td><td></td><td></td><td></td><td></td></tr>
<tr><td>0</td><td>Sequence24Ob7g</td><td></td><td></td><td></td><td></td></tr>
<tr><td>0</td><td>Sequence25Ob7g</td><td></td><td></td><td></td><td></td></tr>
<tr><td>1</td><td>Sequence26Ob7g</td><td>Sequence83Or7g</td><td></td><td></td><td></td></tr>
<tr><td>3</td><td>Sequence27Ob7g</td><td>Sequence84Or7g</td><td>Sequence69Ou7g</td><td>Sequence1Os7g</td><td></td></tr>
<tr><td>4</td><td>Sequence28Ob7g</td><td>Sequence86Or7g</td><td>Sequence70Ou7g</td><td>Sequence3Os7g</td><td>Sequence52Og7g</td></tr>
<tr><td>4</td><td>Sequence29Ob7g</td><td>Sequence89Or7g</td><td>Sequence72Ou7g</td><td>Sequence9Os7g</td><td>Sequence53Og7g</td></tr>
<tr><td>4</td><td>Sequence30Ob7g</td><td>Sequence90Or7g</td><td>Sequence73Ou7g</td><td>Sequence10Os7g</td><td>Sequence54Og7g</td></tr>
<tr><td>4</td><td>Sequence31Ob7g</td><td>Sequence91Or7g</td><td>Sequence74Ou7g</td><td>Sequence11Os7g</td><td>Sequence55Og7g</td></tr>
<tr><td>4</td><td>Sequence32Ob7g</td><td>Sequence92Or7g</td><td>Sequence75Ou7g</td><td>||</td><td>Sequence56Og7g</td></tr>
<tr><td>4</td><td>Sequence33Ob7g</td><td>||</td><td>Sequence76Ou7g</td><td>||</td><td>Sequence57Og7g</td></tr>
<tr><td>4</td><td>Sequence34Ob7g</td><td>Sequence94Or7g</td><td>Sequence77Ou7g</td><td>Sequence13Os7g</td><td>Sequence58Og7g</td></tr>
<tr><td>4</td><td>Sequence35Ob7g</td><td>Sequence95Or7g</td><td>Sequence78Ou7g</td><td>Sequence14Os7g</td><td>Sequence59Og7g</td></tr>
<tr><td>4</td><td>Sequence36Ob7g</td><td>Sequence96Or7g</td><td>Sequence79Ou7g</td><td>Sequence16Os7g</td><td>Sequence60Og7g</td></tr>
<tr><td>4</td><td>Sequence37Ob7g</td><td>Sequence97Or7g</td><td>||</td><td>||</td><td>||</td></tr>
<tr><td>4</td><td>Sequence38Ob7g</td><td>||</td><td>Sequence80Ou7g</td><td>Sequence17Os7g</td><td>Sequence62Og7g</td></tr>
<tr><td>4</td><td>Sequence39Ob7g</td><td>||</td><td>Sequence81Ou7g</td><td>Sequence18Os7g</td><td>Sequence63Og7g</td></tr>
<tr><td>3</td><td>Sequence40Ob7g</td><td>||</td><td></td><td>||</td><td>||</td></tr>
<tr><td>3</td><td>Sequence41Ob7g</td><td>||</td><td></td><td>||</td><td>Sequence65Og7g</td></tr>
<tr><td>3</td><td>Sequence42Ob7g</td><td>||</td><td></td><td>Sequence20Os7g</td><td>Sequence66Og7g</td></tr>
<tr><td>2</td><td>Sequence43Ob7g</td><td>||</td><td></td><td></td><td>Sequence67Og7g</td></tr>
<tr><td>2</td><td>Sequence44Ob7g</td><td>Sequence105Or7g</td><td></td><td></td><td>Sequence68Og7g</td></tr>
<tr><td>0</td><td>Sequence45Ob7g</td><td></td><td></td><td></td><td></td></tr>
<tr><td>0</td><td>Sequence46Ob7g</td><td></td><td></td><td></td><td></td></tr>
</table>

Figure S27: Synteny block diagram for *MIR172C* with *Oryza barthii* as reference. The first column shows duplication depth at each gene locus, second column shows the genes in reference chromosomes and the following is aligned collinear blocks where only match genes are displayed. The alignment among non-anchor genes is discarded in the output and denoted by '||' in the multi-alignment of gene orders. Os7- *Oryza sativa* chr 7, Ob7- *Oryza barthii* chr 7, Og7- *Oryza glaberrima* chr 7, Ou7- *Oryza glumaepatula* chr 7, Or7- *Oryza rufipogon* chr 7, Oa7- *Oryza brachyantha* chr 7, Op2- *Oryza punctata* chr 2, Sb4- *Sorghum bicolor* chr 4, Zm4- *Zea mays* chr 4, At3- *Arabidopsis thaliana* chr 3.

Duplication depth	Reference chromosome	Collinear blocks			
0	Sequence47Og7g				
0	Sequence48Og7g				
0	Sequence49Og7g				
0	Sequence50Og7g				
0	Sequence51Og7g				
4	Sequence52Og7g	Sequence3Os7g	Sequence70Ou7g	Sequence28Ob7g	Sequence86Or7g
4	Sequence53Og7g	Sequence9Os7g	Sequence72Ou7g	Sequence29Ob7g	Sequence89Or7g
4	Sequence54Og7g	Sequence10Os7g	Sequence73Ou7g	Sequence30Ob7g	Sequence90Or7g
4	Sequence55Og7g	Sequence11Os7g	Sequence74Ou7g	Sequence31Ob7g	Sequence91Or7g
4	Sequence56Og7g	||	Sequence75Ou7g	Sequence32Ob7g	Sequence92Or7g
4	Sequence57Og7g	||	Sequence76Ou7g	Sequence33Ob7g	||
4	Sequence58Og7g	Sequence13Os7g	Sequence77Ou7g	Sequence34Ob7g	Sequence94Or7g
4	Sequence59Og7g	Sequence14Os7g	Sequence78Ou7g	Sequence35Ob7g	Sequence95Or7g
4	Sequence60Og7g	Sequence16Os7g	Sequence79Ou7g	Sequence36Ob7g	Sequence96Or7g
4	Sequence61Og7g	||	||	||	||
4	Sequence62Og7g	Sequence17Os7g	Sequence80Ou7g	Sequence38Ob7g	||
4	Sequence63Og7g	Sequence18Os7g	Sequence81Ou7g	Sequence39Ob7g	||
3	Sequence64Og7g	||		||	||
3	Sequence65Og7g	||		Sequence41Ob7g	||
3	Sequence66Og7g	Sequence20Os7g		Sequence42Ob7g	||
2	Sequence67Og7g			Sequence43Ob7g	||
2	Sequence68Og7g			Sequence44Ob7g	Sequence105Or7g

Figure S28: Synteny block diagram for *MIR172C* with *Oryza glaberrima* as reference. The first column shows duplication depth at each gene locus, second column shows the genes in reference chromosomes and the following is aligned collinear blocks where only match genes are displayed. The alignment among non-anchor genes is discarded in the output and denoted by '||' in the multi-alignment of gene orders. Os7- *Oryza sativa* chr 7, Ob7- *Oryza barthii* chr 7, Og7- *Oryza glaberrima* chr 7, Ou7- *Oryza glumaepatula* chr 7, Or7- *Oryza rufipogon* chr 7.

Duplication depth	Reference chromosome	Collinear blocks			
3	Sequence69Ou7g	Sequence27Ob7g	Sequence84Or7g	Sequence1Os7g	
4	Sequence70Ou7g	Sequence28Ob7g	Sequence86Or7g	Sequence3Os7g	Sequence52Og7g
4	Sequence71Ou7g	||	||	||	||
4	Sequence72Ou7g	Sequence29Ob7g	Sequence89Or7g	Sequence9Os7g	Sequence53Og7g
4	Sequence73Ou7g	Sequence30Ob7g	Sequence90Or7g	Sequence10Os7g	Sequence54Og7g
4	Sequence74Ou7g	Sequence31Ob7g	Sequence91Or7g	Sequence11Os7g	Sequence55Og7g
4	Sequence75Ou7g	Sequence32Ob7g	Sequence92Or7g	||	Sequence56Og7g
4	Sequence76Ou7g	Sequence33Ob7g	||	||	Sequence57Og7g
4	Sequence77Ou7g	Sequence34Ob7g	Sequence94Or7g	Sequence13Os7g	Sequence58Og7g
4	Sequence78Ou7g	Sequence35Ob7g	Sequence95Or7g	Sequence14Os7g	Sequence59Og7g
4	Sequence79Ou7g	Sequence36Ob7g	Sequence96Or7g	Sequence16Os7g	Sequence60Og7g
3	Sequence80Ou7g	Sequence38Ob7g		Sequence17Os7g	Sequence62Og7g
3	Sequence81Ou7g	Sequence39Ob7g		Sequence18Os7g	Sequence63Og7g

Figure S29: Synteny block diagram for *MIR172C* with *Oryza glumaepatula* as reference. The first column shows duplication depth at each gene locus, second column shows the genes in reference chromosomes and the following is aligned collinear blocks where only match genes are displayed. The alignment among non-anchor genes is discarded in the output and denoted by '||' in the multi-alignment of gene orders. Os7- *Oryza sativa* chr 7, Ob7- *Oryza barthii* chr 7, Og7- *Oryza glaberrima* chr 7, Ou7- *Oryza glumaepatula* chr 7, Or7- *Oryza rufipogon* chr 7.

<table>
<tr><th>Duplication depth</th><th>Reference chromosome</th><th>Collinear blocks</th><th></th><th></th><th></th></tr>
<tr><td>0</td><td>Sequence82Or7g</td><td></td><td></td><td></td><td></td></tr>
<tr><td>1</td><td>Sequence83Or7g</td><td>Sequence26Ob7g</td><td></td><td></td><td></td></tr>
<tr><td>3</td><td>Sequence84Or7g</td><td>Sequence27Ob7g</td><td>Sequence1Os7g</td><td>Sequence69Ou7g</td><td></td></tr>
<tr><td>3</td><td>Sequence85Or7g</td><td>||</td><td>Sequence2Os7g</td><td>||</td><td></td></tr>
<tr><td>4</td><td>Sequence86Or7g</td><td>Sequence28Ob7g</td><td>Sequence3Os7g</td><td>Sequence70Ou7g</td><td>Sequence52Og7g</td></tr>
<tr><td>4</td><td>Sequence87Or7g</td><td>||</td><td>Sequence4Os7g</td><td>||</td><td>||</td></tr>
<tr><td>4</td><td>Sequence88Or7g</td><td>||</td><td>Sequence6Os7g</td><td>||</td><td>||</td></tr>
<tr><td>4</td><td>Sequence89Or7g</td><td>Sequence29Ob7g</td><td>Sequence9Os7g</td><td>Sequence72Ou7g</td><td>Sequence53Og7g</td></tr>
<tr><td>4</td><td>Sequence90Or7g</td><td>Sequence30Ob7g</td><td>Sequence10Os7g</td><td>Sequence73Ou7g</td><td>Sequence54Og7g</td></tr>
<tr><td>4</td><td>Sequence91Or7g</td><td>Sequence31Ob7g</td><td>Sequence11Os7g</td><td>Sequence74Ou7g</td><td>Sequence55Og7g</td></tr>
<tr><td>4</td><td>Sequence92Or7g</td><td>Sequence32Ob7g</td><td>||</td><td>Sequence75Ou7g</td><td>Sequence56Og7g</td></tr>
<tr><td>4</td><td>Sequence93Or7g</td><td>||</td><td>Sequence12Os7g</td><td>||</td><td>||</td></tr>
<tr><td>4</td><td>Sequence94Or7g</td><td>Sequence34Ob7g</td><td>Sequence13Os7g</td><td>Sequence77Ou7g</td><td>Sequence58Og7g</td></tr>
<tr><td>4</td><td>Sequence95Or7g</td><td>Sequence35Ob7g</td><td>Sequence15Os7g</td><td>Sequence78Ou7g</td><td>Sequence59Og7g</td></tr>
<tr><td>4</td><td>Sequence96Or7g</td><td>Sequence36Ob7g</td><td>Sequence16Os7g</td><td>Sequence79Ou7g</td><td>Sequence60Og7g</td></tr>
<tr><td>2</td><td>Sequence97Or7g</td><td>Sequence37Ob7g</td><td></td><td></td><td>||</td></tr>
<tr><td>2</td><td>Sequence98Or7g</td><td>||</td><td></td><td></td><td>||</td></tr>
<tr><td>2</td><td>Sequence99Or7g</td><td>||</td><td></td><td></td><td>||</td></tr>
<tr><td>2</td><td>Sequence100Or7g</td><td>||</td><td></td><td></td><td>||</td></tr>
<tr><td>2</td><td>Sequence101Or7g</td><td>||</td><td></td><td></td><td>||</td></tr>
<tr><td>2</td><td>Sequence102Or7g</td><td>||</td><td></td><td></td><td>||</td></tr>
<tr><td>2</td><td>Sequence103Or7g</td><td>||</td><td></td><td></td><td>||</td></tr>
<tr><td>2</td><td>Sequence104Or7g</td><td>||</td><td></td><td></td><td>||</td></tr>
<tr><td>2</td><td>Sequence105Or7g</td><td>Sequence44Ob7g</td><td></td><td></td><td>Sequence68Og7g</td></tr>
<tr><td>0</td><td>Sequence106Or7g</td><td></td><td></td><td></td><td></td></tr>
<tr><td>0</td><td>Sequence107Or7g</td><td></td><td></td><td></td><td></td></tr>
<tr><td>0</td><td>Sequence108Or7g</td><td></td><td></td><td></td><td></td></tr>
<tr><td>0</td><td>Sequence109Or7g</td><td></td><td></td><td></td><td></td></tr>
</table>

Figure S30: Synteny block diagram for *MIR172C* with *Oryza rufipogon* as reference. The first column shows duplication depth at each gene locus, second column shows the genes in reference chromosomes and the following is aligned collinear blocks where only match genes are displayed. The alignment among non-anchor genes is discarded in the output and denoted by '||' in the multi-alignment of gene orders. Os7- *Oryza sativa* chr 7, Ob7- *Oryza barthii* chr 7, Og7- *Oryza glaberrima* chr 7, Ou7- *Oryza glumaepatula* chr 7, Or7- *Oryza rufipogon* chr 7.

Duplication depth	Reference chromosome	Collinear blocks
0	Sequence110Oa7g	
0	Sequence111Oa7g	
0	Sequence112Oa7g	
0	Sequence113Oa7g	
0	Sequence114Oa7g	
0	Sequence115Oa7g	
0	Sequence116Oa7g	
0	Sequence117Oa7g	
0	Sequence118Oa7g	
0	Sequence119Oa7g	
0	Sequence120Oa7g	
0	Sequence121Oa7g	
0	Sequence122Oa7g	
0	Sequence123Oa7g	
0	Sequence124Oa7g	
0	Sequence125Oa7g	
0	Sequence126Oa7g	
0	Sequence127Oa7g	
0	Sequence128Oa7g	
0	Sequence129Oa7g	
0	Sequence130Oa7g	

Figure S31: Synteny block diagram for *MIR172C* with *Oryza brachyantha* as reference. The first column shows duplication depth at each gene locus, second column shows the genes in reference chromosomes and the following is aligned collinear blocks where only match genes are displayed. The alignment among non-anchor genes is discarded in the output and denoted by '||' in the multi-alignment of gene orders. Oa7 - *Oryza brachyantha* chr 7.

Duplication depth	Reference chromosome	Collinear blocks
0	Sequence131Op2g	
0	Sequence132Op2g	
0	Sequence133Op2g	
0	Sequence134Op2g	
0	Sequence135Op2g	
0	Sequence136Op2g	
0	Sequence137Op2g	
0	Sequence138Op2g	
0	Sequence139Op2g	
0	Sequence140Op2g	
0	Sequence141Op2g	
0	Sequence142Op2g	
0	Sequence143Op2g	
0	Sequence144Op2g	
0	Sequence145Op2g	
0	Sequence146Op2g	
0	Sequence147Op2g	
0	Sequence148Op2g	
0	Sequence149Op2g	
0	Sequence150Op2g	
0	Sequence151Op2g	
0	Sequence152Op2g	

Figure S32: Synteny block diagram for *MIR172C* with *Oryza punctata* as reference. The first column shows duplication depth at each gene locus, second column shows the genes in reference chromosomes and the following is aligned collinear blocks where only match genes are displayed. The alignment among non-anchor genes is discarded in the output and denoted by '||' in the multi-alignment of gene orders. Oa7 - *Oryza punctata* chr 2.

Duplication depth	Reference chromosome	Collinear blocks
0	Sequence153Sb4g	
0	Sequence154Sb4g	
0	Sequence155Sb4g	
0	Sequence156Sb4g	
0	Sequence157Sb4g	
0	Sequence158Sb4g	
0	Sequence159Sb4g	
0	Sequence160Sb4g	
0	Sequence161Sb4g	
0	Sequence162Sb4g	
0	Sequence163Sb4g	
0	Sequence164Sb4g	
0	Sequence165Sb4g	
0	Sequence166Sb4g	
0	Sequence167Sb4g	
0	Sequence168Sb4g	
0	Sequence169Sb4g	
0	Sequence170Sb4g	

Figure S33: Synteny block diagram for *MIR172C* with *Sorghum bicolor* as reference. The first column shows duplication depth at each gene locus, second column shows the genes in reference chromosomes and the following is aligned collinear blocks where only match genes are displayed. The alignment among non-anchor genes is discarded in the output and denoted by '||' in the multi-alignment of gene orders. Sb4 - *Sorghum bicolor* chr 4.

cells

MDPI

Duplication depth	Reference chromosome	Collinear blocks
0	Sequence171Zm4g	
0	Sequence172Zm4g	
0	Sequence173Zm4g	
0	Sequence174Zm4g	
0	Sequence175Zm4g	
0	Sequence176Zm4g	
0	Sequence177Zm4g	
0	Sequence178Zm4g	
0	Sequence179Zm4g	
0	Sequence180Zm4g	
0	Sequence181Zm4g	
0	Sequence182Zm4g	

Figure S34: Synteny block diagram for *MIR172C* with *Zea mays* as reference. The first column shows duplication depth at each gene locus, second column shows the genes in reference chromosomes and the following is aligned collinear blocks where only match genes are displayed. The alignment among non-anchor genes is discarded in the output and denoted by '||' in the multi-alignment of gene orders. Zm4-*Zea mays* chr 4.

Duplication depth	Reference chromosome	Collinear blocks
0	Sequence183At3g	
0	Sequence184At3g	
0	Sequence185At3g	
0	Sequence186At3g	
0	Sequence187At3g	
0	Sequence188At3g	
0	Sequence189At3g	
0	Sequence190At3g	
0	Sequence191At3g	
0	Sequence192At3g	
0	Sequence193At3g	
0	Sequence194At3g	
0	Sequence195At3g	
0	Sequence196At3g	
0	Sequence197At3g	
0	Sequence198At3g	
0	Sequence199At3g	
0	Sequence200At3g	
0	Sequence201At3g	
0	Sequence202At3g	
0	Sequence203At3g	
0	Sequence204At3g	
0	Sequence205At3g	
0	Sequence206At3g	
0	Sequence207At3g	
0	Sequence208At3g	
0	Sequence209At3g	
0	Sequence210At3g	
0	Sequence211At3g	

Figure S35: Synteny block diagram for *MIR172C* with *Arabidopsis thaliana* as reference. The first column shows duplication depth at each gene locus, second column shows the genes in reference chromosomes and the following is aligned collinear blocks where only match genes are displayed. The alignment among non-anchor genes is discarded in the output and denoted by '||' in the multi-alignment of gene orders. At3 - *Arabidopsis thaliana* chr 3.

Duplication depth	Reference chromosome	Collinear blocks					
1	Sequence43Ob2g	Sequence2Osi2g					
4	Sequence44Ob2g	Sequence3Osi2g	Sequence65Og2g	Sequence143Op2g	Sequence107Or2g		
5	Sequence45Ob2g	Sequence4Osi2g	Sequence66Og2g	Sequence144Op2g	Sequence108Or2g	Sequence88Ou2g	
5	Sequence46Ob2g	||	Sequence67Og2g	||	||	||	
6	Sequence47Ob2g	Sequence7Osi2g	Sequence68Og2g	Sequence146Op2g	Sequence110Or2g	Sequence90Ou2g	Sequence24Osii2g
6	Sequence48Ob2g	Sequence8Osi2g	Sequence69Og2g	Sequence149Op2g	||	Sequence94Ou2g	Sequence25Osii2g
6	Sequence49Ob2g	Sequence13Osi2g	Sequence70Og2g	Sequence150Op2g	Sequence111Or2g	Sequence95Ou2g	Sequence30Osii2g
6	Sequence50Ob2g	||	Sequence73Og2g	||	||	||	||
6	Sequence51Ob2g	||	Sequence74Og2g	Sequence151Op2g	Sequence113Or2g	Sequence96Ou2g	Sequence32Osii2g
6	Sequence52Ob2g	Sequence15Osi2g	||	Sequence152Op2g	Sequence114Or2g	||	||
6	Sequence53Ob2g	Sequence16Osi2g	Sequence76Og2g	||	Sequence115Or2g	||	Sequence33Osii2g
6	Sequence54Ob2g	||	Sequence77Og2g	||	||	Sequence98Ou2g	||
6	Sequence55Ob2g	Sequence18Osi2g	Sequence78Og2g	||	Sequence117Or2g	Sequence99Ou2g	Sequence35Osii2g
6	Sequence56Ob2g	Sequence19Osi2g	Sequence79Og2g	Sequence154Op2g	Sequence118Or2g	Sequence100Ou2g	Sequence36Osii2g
6	Sequence57Ob2g	Sequence20Osi2g	Sequence80Og2g	||	Sequence119Or2g	Sequence101Ou2g	Sequence37Osii2g
6	Sequence58Ob2g	Sequence21Osi2g	Sequence81Og2g	Sequence155Op2g	Sequence120Or2g	Sequence102Ou2g	Sequence38Osii2g
5	Sequence59Ob2g		Sequence82Og2g	Sequence157Op2g	Sequence121Or2g	Sequence103Ou2g	Sequence39Osii2g
5	Sequence60Ob2g		Sequence83Og2g	||	||	||	||
5	Sequence61Ob2g		Sequence84Og2g	Sequence158Op2g	Sequence122Or2g	Sequence104Ou2g	Sequence40Osii2g
5	Sequence62Ob2g		Sequence85Og2g	Sequence159Op2g	Sequence123Or2g	Sequence105Ou2g	Sequence41Osii2g
5	Sequence63Ob2g		Sequence86Og2g	Sequence160Op2g	Sequence124Or2g	Sequence106Ou2g	Sequence42Osii2g
2	Sequence64Ob2g		Sequence87Og2g	Sequence163Op2g			

Figure S36: Synteny block diagram for *MIR172D* with *Oryza barthii* as reference. The first column shows duplication depth at each gene locus, second column shows the genes in reference chromosomes and the following is aligned collinear blocks where only match genes are displayed. The alignment among non-anchor genes is discarded in the output and denoted by '||' in the multi-alignment of gene orders. Osi2- *Oryza sativa* homeolog i chr 2, Osii2- *Oryza sativa* homeolog ii chr 2, Ob2- *Oryza barthii* chr 2, Og2- *Oryza glaberrima* chr 2, Ou2- *Oryza glumaepatula* chr 2, Or2- *Oryza rufipogon* chr 2, Op2- *Oryza punctata* chr 2.

<table>
<tr><th>Duplication depth</th><th>Reference chromosome</th><th>Collinear blocks</th><th></th><th></th><th></th><th></th><th></th></tr>
<tr><td>4</td><td>Sequence65Og2g</td><td>Sequence44Ob2g</td><td>Sequence3Osi2g</td><td>Sequence107Or2g</td><td>Sequence143Op2g</td><td></td><td></td></tr>
<tr><td>5</td><td>Sequence66Og2g</td><td>Sequence45Ob2g</td><td>Sequence4Osi2g</td><td>Sequence108Or2g</td><td>Sequence144Op2g</td><td>Sequence88Ou2g</td><td></td></tr>
<tr><td>5</td><td>Sequence67Og2g</td><td>Sequence46Ob2g</td><td>||</td><td>||</td><td>||</td><td>||</td><td></td></tr>
<tr><td>6</td><td>Sequence68Og2g</td><td>Sequence47Ob2g</td><td>Sequence7Osi2g</td><td>||</td><td>Sequence146Op2g</td><td>Sequence90Ou2g</td><td>Sequence24Osii2g</td></tr>
<tr><td>6</td><td>Sequence69Og2g</td><td>Sequence48Ob2g</td><td>Sequence8Osi2g</td><td>Sequence109Or2g</td><td>Sequence149Op2g</td><td>Sequence94Ou2g</td><td>Sequence25Osii2g</td></tr>
<tr><td>6</td><td>Sequence70Og2g</td><td>Sequence49Ob2g</td><td>Sequence13Osi2g</td><td>||</td><td>||</td><td>Sequence95Ou2g</td><td>Sequence30Osii2g</td></tr>
<tr><td>6</td><td>Sequence71Og2g</td><td>||</td><td>||</td><td>||</td><td>||</td><td>||</td><td>||</td></tr>
<tr><td>6</td><td>Sequence72Og2g</td><td>||</td><td>||</td><td>||</td><td>Sequence150Op2g</td><td>||</td><td>||</td></tr>
<tr><td>6</td><td>Sequence73Og2g</td><td>Sequence50Ob2g</td><td>||</td><td>||</td><td>||</td><td>||</td><td>||</td></tr>
<tr><td>6</td><td>Sequence74Og2g</td><td>Sequence51Ob2g</td><td>Sequence15Osi2g</td><td>Sequence113Or2g</td><td>Sequence151Op2g</td><td>Sequence96Ou2g</td><td>Sequence32Osii2g</td></tr>
<tr><td>6</td><td>Sequence75Og2g</td><td>||</td><td>||</td><td>||</td><td>||</td><td>||</td><td>||</td></tr>
<tr><td>6</td><td>Sequence76Og2g</td><td>Sequence53Ob2g</td><td>Sequence16Osi2g</td><td>Sequence115Or2g</td><td>||</td><td>||</td><td>Sequence33Osii2g</td></tr>
<tr><td>6</td><td>Sequence77Og2g</td><td>Sequence54Ob2g</td><td>Sequence17Osi2g</td><td>Sequence116Or2g</td><td>Sequence153Op2g</td><td>Sequence98Ou2g</td><td>Sequence34Osii2g</td></tr>
<tr><td>6</td><td>Sequence78Og2g</td><td>Sequence55Ob2g</td><td>Sequence18Osi2g</td><td>Sequence117Or2g</td><td>||</td><td>Sequence99Ou2g</td><td>Sequence35Osii2g</td></tr>
<tr><td>6</td><td>Sequence79Og2g</td><td>Sequence56Ob2g</td><td>Sequence19Osi2g</td><td>Sequence118Or2g</td><td>Sequence154Op2g</td><td>Sequence100Ou2g</td><td>Sequence36Osii2g</td></tr>
<tr><td>6</td><td>Sequence80Og2g</td><td>Sequence57Ob2g</td><td>Sequence20Osi2g</td><td>Sequence119Or2g</td><td>||</td><td>Sequence101Ou2g</td><td>Sequence37Osii2g</td></tr>
<tr><td>6</td><td>Sequence81Og2g</td><td>Sequence58Ob2g</td><td>Sequence21Osi2g</td><td>Sequence120Or2g</td><td>Sequence155Op2g</td><td>Sequence102Ou2g</td><td>Sequence38Osii2g</td></tr>
<tr><td>6</td><td>Sequence82Og2g</td><td>Sequence59Ob2g</td><td>Sequence22Osi2g</td><td>Sequence121Or2g</td><td>Sequence156Op2g</td><td>Sequence103Ou2g</td><td>Sequence39Osii2g</td></tr>
<tr><td>5</td><td>Sequence83Og2g</td><td>Sequence60Ob2g</td><td></td><td>||</td><td>||</td><td>||</td><td>||</td></tr>
<tr><td>5</td><td>Sequence84Og2g</td><td>Sequence61Ob2g</td><td></td><td>Sequence122Or2g</td><td>Sequence158Op2g</td><td>Sequence104Ou2g</td><td>||</td></tr>
<tr><td>5</td><td>Sequence85Og2g</td><td>Sequence62Ob2g</td><td></td><td>Sequence123Or2g</td><td>Sequence159Op2g</td><td>Sequence105Ou2g</td><td>Sequence41Osii2g</td></tr>
<tr><td>5</td><td>Sequence86Og2g</td><td>Sequence63Ob2g</td><td></td><td>||</td><td>Sequence160Op2g</td><td>Sequence106Ou2g</td><td>Sequence42Osii2g</td></tr>
<tr><td>3</td><td>Sequence87Og2g</td><td>Sequence64Ob2g</td><td></td><td>Sequence124Or2g</td><td>Sequence163Op2g</td><td></td><td></td></tr>
</table>

Figure S37: Synteny block diagram for *MIR172D* with *Oryza glaberrima* as reference. The first column shows duplication depth at each gene locus, second column shows the genes in reference chromosomes and the following is aligned collinear blocks where only match genes are displayed. The alignment among non-anchor genes is discarded in the output and denoted by '||' in the multi-alignment of gene orders. Osi2- *Oryza sativa* homeolog i chr 2, Osii2- *Oryza sativa* homeolog ii chr 2, Ob2- *Oryza barthii* chr 2, Og2- *Oryza glaberrima* chr 2, Ou2- *Oryza glumaepatula* chr 2, Or2- *Oryza rufipogon* chr 2, Op2- *Oryza punctata* chr 2.

Duplication depth	Reference chromosome	Collinear blocks					
4	Sequence88Ou2g	Sequence66Og2g	Sequence108Or2g	Sequence4Osi2g	Sequence45Ob2g		
4	Sequence89Ou2g	||	Sequence109Or2g	||	||		
6	Sequence90Ou2g	Sequence68Og2g	||	||	Sequence47Ob2g	Sequence146Op2g	Sequence27Osii2g
6	Sequence91Ou2g	||	||	||	||	||	||
6	Sequence92Ou2g	||	Sequence110Or2g	Sequence7Osi2g	||	||	||
6	Sequence93Ou2g	||	||	||	||	||	||
6	Sequence94Ou2g	Sequence69Og2g	||	Sequence11Osi2g	Sequence48Ob2g	Sequence149Op2g	Sequence28Osii2g
6	Sequence95Ou2g	Sequence70Og2g	Sequence111Or2g	Sequence13Osi2g	Sequence49Ob2g	Sequence150Op2g	Sequence30Osii2g
6	Sequence96Ou2g	Sequence74Og2g	Sequence113Or2g	Sequence15Osi2g	Sequence51Ob2g	Sequence151Op2g	Sequence32Osii2g
6	Sequence97Ou2g	||	||	||	||	||	||
6	Sequence98Ou2g	Sequence77Og2g	Sequence116Or2g	Sequence17Osi2g	Sequence54Ob2g	||	Sequence34Osii2g
6	Sequence99Ou2g	Sequence78Og2g	Sequence117Or2g	Sequence18Osi2g	Sequence55Ob2g	||	Sequence35Osii2g
6	Sequence100Ou2g	Sequence79Og2g	Sequence118Or2g	Sequence19Osi2g	Sequence56Ob2g	Sequence154Op2g	Sequence36Osii2g
6	Sequence101Ou2g	Sequence80Og2g	Sequence119Or2g	Sequence20Osi2g	Sequence57Ob2g	||	Sequence37Osii2g
6	Sequence102Ou2g	Sequence81Og2g	Sequence120Or2g	||	Sequence58Ob2g	Sequence155Op2g	||
6	Sequence103Ou2g	Sequence82Og2g	Sequence121Or2g	Sequence22Osi2g	Sequence59Ob2g	Sequence156Op2g	Sequence39Osii2g
5	Sequence104Ou2g	Sequence84Og2g	Sequence122Or2g		Sequence61Ob2g	Sequence158Op2g	Sequence40Osii2g
5	Sequence105Ou2g	Sequence85Og2g	Sequence123Or2g		Sequence62Ob2g	Sequence159Op2g	Sequence41Osii2g
4	Sequence106Ou2g	Sequence86Og2g	Sequence124Or2g		Sequence63Ob2g		Sequence42Osii2g

Figure S38: Synteny block diagram for *MIR172D* with *Oryza glumaepatula* as reference. The first column shows duplication depth at each gene locus, second column shows the genes in reference chromosomes and the following is aligned collinear blocks where only match genes are displayed. The alignment among non-anchor genes is discarded in the output and denoted by '||' in the multi-alignment of gene orders. Osi2- *Oryza sativa* homeolog i chr 2, Osii2- *Oryza sativa* homeolog ii chr 2, Ob2- *Oryza barthii* chr 2, Og2- *Oryza glaberrima* chr 2, Ou2- *Oryza glumaepatula* chr 2, Or2- *Oryza rufipogon* chr 2, Op2- *Oryza punctata* chr 2.

Duplication depth	Reference chromosome	Collinear blocks					
4	Sequence107Or2g	Sequence65Og2g	Sequence3Osi2g	Sequence44Ob2g	Sequence143Op2g		
5	Sequence108Or2g	Sequence66Og2g	Sequence4Osi2g	Sequence45Ob2g	Sequence144Op2g	Sequence88Ou2g	
5	Sequence109Or2g	Sequence69Og2g	||	||	||	Sequence89Ou2g	
6	Sequence110Or2g	||	Sequence8Osi2g	Sequence47Ob2g	Sequence146Op2g	Sequence92Ou2g	Sequence25Osii2g
6	Sequence111Or2g	||	Sequence13Osi2g	Sequence49Ob2g	||	Sequence95Ou2g	Sequence30Osii2g
6	Sequence112Or2g	||	||	||	||	||	||
6	Sequence113Or2g	Sequence74Og2g	||	Sequence51Ob2g	Sequence151Op2g	Sequence96Ou2g	Sequence32Osii2g
6	Sequence114Or2g	||	||	Sequence52Ob2g	||	||	||
6	Sequence115Or2g	Sequence76Og2g	Sequence16Osi2g	Sequence53Ob2g	||	||	Sequence33Osii2g
6	Sequence116Or2g	Sequence77Og2g	Sequence17Osi2g	||	Sequence153Op2g	Sequence98Ou2g	Sequence34Osii2g
6	Sequence117Or2g	Sequence78Og2g	Sequence18Osi2g	Sequence55Ob2g	||	Sequence99Ou2g	Sequence35Osii2g
6	Sequence118Or2g	Sequence79Og2g	Sequence19Osi2g	Sequence56Ob2g	Sequence154Op2g	Sequence100Ou2g	Sequence36Osii2g
6	Sequence119Or2g	Sequence80Og2g	Sequence20Osi2g	Sequence57Ob2g	||	Sequence101Ou2g	Sequence37Osii2g
6	Sequence120Or2g	Sequence81Og2g	Sequence21Osi2g	Sequence58Ob2g	Sequence155Op2g	Sequence102Ou2g	Sequence38Osii2g
6	Sequence121Or2g	Sequence82Og2g	Sequence22Osi2g	Sequence59Ob2g	Sequence156Op2g	Sequence103Ou2g	Sequence39Osii2g
5	Sequence122Or2g	Sequence84Og2g		Sequence61Ob2g	Sequence158Op2g	Sequence104Ou2g	Sequence40Osii2g
5	Sequence123Or2g	Sequence85Og2g		Sequence62Ob2g	||	Sequence105Ou2g	Sequence41Osii2g
5	Sequence124Or2g	Sequence87Og2g		Sequence63Ob2g	Sequence163Op2g	Sequence106Ou2g	Sequence42Osii2g

Figure S39: Synteny block diagram for *MIR172D* with *Oryza rufipogon* as reference. The first column shows duplication depth at each gene locus, second column shows the genes in reference chromosomes and the following is aligned collinear blocks where only match genes are displayed. The alignment among non-anchor genes is discarded in the output and denoted by '||' in the multi-alignment of gene orders. Osi2- *Oryza sativa* homeolog i chr 2, Osii2- *Oryza sativa* homeolog ii chr 2, Ob2- *Oryza barthii* chr 2, Og2- *Oryza glaberrima* chr 2, Ou2- *Oryza glumaepatula* chr 2, Or2- *Oryza rufipogon* chr 2, Op2- *Oryza punctata* chr 2.

Duplication depth	Reference chromosome	Collinear blocks
0	Sequence125Oa2g	
0	Sequence126Oa2g	
0	Sequence127Oa2g	
0	Sequence128Oa2g	
0	Sequence129Oa2g	
0	Sequence130Oa2g	
0	Sequence131Oa2g	
0	Sequence132Oa2g	
0	Sequence133Oa2g	
0	Sequence134Oa2g	
0	Sequence135Oa2g	
0	Sequence136Oa2g	
0	Sequence137Oa2g	
0	Sequence138Oa2g	
0	Sequence139Oa2g	
0	Sequence140Oa2g	
0	Sequence141Oa2g	

Figure S40: Synteny block diagram for *MIR172D* with *Oryza brachyantha* as reference. The first column shows duplication depth at each gene locus, second column shows the genes in reference chromosomes and the following is aligned collinear blocks where only match genes are displayed. The alignment among non-anchor genes is discarded in the output and denoted by '||' in the multi-alignment of gene orders. Oa2- *Oryza brachyantha* chr 2.

cells

MDPI

Duplication depth	Reference chromosome	Collinear blocks					
0	Sequence142Op2g						
4	Sequence143Op2g	Sequence3Osi2g	Sequence107Or2g	Sequence65Og2g	Sequence44Ob2g		
4	Sequence144Op2g	Sequence4Osi2g	Sequence108Or2g	Sequence66Og2g	Sequence45Ob2g		
4	Sequence145Op2g	Sequence5Osi2g	||	||	||		
6	Sequence146Op2g	Sequence7Osi2g	Sequence110Or2g	Sequence68Og2g	Sequence47Ob2g	Sequence24Osii2g	Sequence90Ou2g
6	Sequence147Op2g	||	||	||	||	||	||
6	Sequence148Op2g	||	||	||	||	||	||
6	Sequence149Op2g	||	||	Sequence69Og2g	Sequence48Ob2g	||	Sequence94Ou2g
6	Sequence150Op2g	Sequence13Osi2g	||	Sequence72Og2g	Sequence49Ob2g	Sequence30Osii2g	Sequence95Ou2g
6	Sequence151Op2g	||	Sequence113Or2g	Sequence74Og2g	Sequence51Ob2g	||	Sequence96Ou2g
6	Sequence152Op2g	Sequence15Osi2g	||	||	Sequence52Ob2g	Sequence32Osii2g	||
6	Sequence153Op2g	Sequence17Osi2g	Sequence116Or2g	Sequence77Og2g	||	Sequence34Osii2g	||
5	Sequence154Op2g		Sequence118Or2g	Sequence79Og2g	Sequence56Ob2g	||	Sequence100Ou2g
5	Sequence155Op2g		Sequence120Or2g	Sequence81Og2g	Sequence58Ob2g	||	Sequence102Ou2g
5	Sequence156Op2g		Sequence121Or2g	Sequence82Og2g	||	Sequence39Osii2g	Sequence103Ou2g
5	Sequence157Op2g		||	||	Sequence59Ob2g	||	||
5	Sequence158Op2g		Sequence122Or2g	Sequence84Og2g	Sequence61Ob2g	Sequence40Osii2g	Sequence104Ou2g
5	Sequence159Op2g		||	Sequence85Og2g	Sequence62Ob2g	Sequence41Osii2g	Sequence105Ou2g
4	Sequence160Op2g		||	Sequence86Og2g	Sequence63Ob2g	Sequence42Osii2g	
3	Sequence161Op2g		||	||	||		
3	Sequence162Op2g		||	||	||		
3	Sequence163Op2g		Sequence124Or2g	Sequence87Og2g	Sequence64Ob2g		

Figure S41: Synteny block diagram for *MIR172D* with *Oryza punctata* as reference. The first column shows duplication depth at each gene locus, second column shows the genes in reference chromosomes and the following is aligned collinear blocks where only match genes are displayed. The alignment among non-anchor genes is discarded in the output and denoted by '||' in the multi-alignment of gene orders. Osi2- *Oryza sativa* homeolog i chr 2, Osii2- *Oryza sativa* homeolog ii chr 2, Ob2- *Oryza barthii* chr 2, Og2- *Oryza glaberrima* chr 2, Ou2- *Oryza glumaepatula* chr 2, Or2- *Oryza rufipogon* chr 2, Op2- *Oryza punctata* chr 2.

<table>
<tr><th>Duplication depth</th><th>Reference chromosome</th><th>Collinear blocks</th><th></th><th></th><th></th><th></th><th></th></tr>
<tr><td>4</td><td>Sequence24Osii2g</td><td>Sequence146Op2g</td><td>Sequence7Osi2g</td><td>Sequence68Og2g</td><td>Sequence47Ob2g</td><td></td><td></td></tr>
<tr><td>5</td><td>Sequence25Osii2g</td><td>||</td><td>Sequence8Osi2g</td><td>Sequence69Og2g</td><td>Sequence48Ob2g</td><td>Sequence110Or2g</td><td></td></tr>
<tr><td>5</td><td>Sequence26Osii2g</td><td>||</td><td>Sequence9Osi2g</td><td>||</td><td>||</td><td>||</td><td></td></tr>
<tr><td>6</td><td>Sequence27Osii2g</td><td>||</td><td>Sequence10Osi2g</td><td>||</td><td>||</td><td>||</td><td>Sequence90Ou2g</td></tr>
<tr><td>6</td><td>Sequence28Osii2g</td><td>||</td><td>Sequence11Osi2g</td><td>||</td><td>||</td><td>||</td><td>Sequence94Ou2g</td></tr>
<tr><td>6</td><td>Sequence29Osii2g</td><td>||</td><td>Sequence12Osi2g</td><td>||</td><td>||</td><td>||</td><td>||</td></tr>
<tr><td>6</td><td>Sequence30Osii2g</td><td>Sequence150Op2g</td><td>Sequence13Osi2g</td><td>Sequence70Og2g</td><td>Sequence49Ob2g</td><td>Sequence111Or2g</td><td>Sequence95Ou2g</td></tr>
<tr><td>6</td><td>Sequence31Osii2g</td><td>||</td><td>Sequence14Osi2g</td><td>||</td><td>||</td><td>||</td><td>||</td></tr>
<tr><td>6</td><td>Sequence32Osii2g</td><td>Sequence152Op2g</td><td>Sequence15Osi2g</td><td>Sequence74Og2g</td><td>Sequence51Ob2g</td><td>Sequence113Or2g</td><td>Sequence96Ou2g</td></tr>
<tr><td>6</td><td>Sequence33Osii2g</td><td>||</td><td>Sequence16Osi2g</td><td>Sequence76Og2g</td><td>Sequence53Ob2g</td><td>Sequence115Or2g</td><td>||</td></tr>
<tr><td>6</td><td>Sequence34Osii2g</td><td>Sequence153Op2g</td><td>Sequence17Osi2g</td><td>Sequence77Og2g</td><td>||</td><td>Sequence116Or2g</td><td>Sequence98Ou2g</td></tr>
<tr><td>6</td><td>Sequence35Osii2g</td><td>||</td><td>Sequence18Osi2g</td><td>Sequence78Og2g</td><td>Sequence55Ob2g</td><td>Sequence117Or2g</td><td>Sequence99Ou2g</td></tr>
<tr><td>6</td><td>Sequence36Osii2g</td><td>||</td><td>Sequence19Osi2g</td><td>Sequence79Og2g</td><td>Sequence56Ob2g</td><td>Sequence118Or2g</td><td>Sequence100Ou2g</td></tr>
<tr><td>6</td><td>Sequence37Osii2g</td><td>||</td><td>Sequence20Osi2g</td><td>Sequence80Og2g</td><td>Sequence57Ob2g</td><td>Sequence119Or2g</td><td>Sequence101Ou2g</td></tr>
<tr><td>6</td><td>Sequence38Osii2g</td><td>||</td><td>Sequence21Osi2g</td><td>Sequence81Og2g</td><td>Sequence58Ob2g</td><td>Sequence120Or2g</td><td>||</td></tr>
<tr><td>6</td><td>Sequence39Osii2g</td><td>Sequence156Op2g</td><td>Sequence22Osi2g</td><td>Sequence82Og2g</td><td>Sequence59Ob2g</td><td>Sequence121Or2g</td><td>Sequence103Ou2g</td></tr>
<tr><td>5</td><td>Sequence40Osii2g</td><td>Sequence158Op2g</td><td></td><td>||</td><td>Sequence61Ob2g</td><td>Sequence122Or2g</td><td>Sequence104Ou2g</td></tr>
<tr><td>5</td><td>Sequence41Osii2g</td><td>Sequence159Op2g</td><td></td><td>Sequence85Og2g</td><td>Sequence62Ob2g</td><td>Sequence123Or2g</td><td>Sequence105Ou2g</td></tr>
<tr><td>5</td><td>Sequence42Osii2g</td><td>Sequence160Op2g</td><td></td><td>Sequence86Og2g</td><td>Sequence63Ob2g</td><td>Sequence124Or2g</td><td>Sequence106Ou2g</td></tr>
</table>

Figure S42: Synteny block diagram for *MIR172D* with *Oryza sativa* (ii) as reference. The first column shows duplication depth at each gene locus, second column shows the genes in reference chromosomes and the following is aligned collinear blocks where only match genes are displayed. The alignment among non-anchor genes is discarded in the output and denoted by '||' in the multi-alignment of gene orders. Osi2- *Oryza sativa* homeolog i chr 2, Osii2- *Oryza sativa* homeolog ii chr 2, Ob2- *Oryza barthii* chr 2, Og2- *Oryza glaberrima* chr 2, Ou2- *Oryza glumaepatula* chr 2, Or2- *Oryza rufipogon* chr 2, Op2- *Oryza punctata* chr 2.

Duplication depth	Reference chromosome	Collinear blocks
0	Sequence164Sb2g	
0	Sequence165Sb2g	
0	Sequence166Sb2g	
0	Sequence167Sb2g	
0	Sequence168Sb2g	
0	Sequence169Sb2g	
0	Sequence170Sb2g	
0	Sequence171Sb2g	
0	Sequence172Sb2g	
0	Sequence173Sb2g	
0	Sequence174Sb2g	
0	Sequence175Sb2g	
0	Sequence176Sb2g	
0	Sequence177Sb2g	
0	Sequence178Sb2g	
0	Sequence179Sb2g	
0	Sequence180Sb2g	
0	Sequence181Sb2g	
0	Sequence182Sb2g	
0	Sequence183Sb2g	
0	Sequence184Sb2g	
0	Sequence185Sb2g	
0	Sequence186Sb2g	
0	Sequence187Sb2g	
0	Sequence188Sb2g	
0	Sequence189Sb2g	
0	Sequence190Sb2g	

Figure S43: Synteny block diagram for *MIR172D* with *Sorghum bicolor* as reference. The first column shows duplication depth at each gene locus, second column shows the genes in reference chromosomes and the following is aligned collinear blocks where only match genes are displayed. The alignment among non-anchor genes is discarded in the output and denoted by '||' in the multi-alignment of gene orders. Sb2 - *Sorghum bicolor* chr 2.

Duplication depth	Reference chromosome	Collinear blocks
0	Sequence191Zm6g	
0	Sequence192Zm6g	
0	Sequence193Zm6g	
0	Sequence194Zm6g	
0	Sequence195Zm6g	
0	Sequence196Zm6g	
0	Sequence197Zm6g	
0	Sequence198Zm6g	
0	Sequence199Zm6g	
0	Sequence200Zm6g	
0	Sequence201Zm6g	
0	Sequence202Zm6g	
0	Sequence203Zm6g	
0	Sequence204Zm6g	
0	Sequence205Zm6g	
0	Sequence206Zm6g	
0	Sequence207Zm6g	
0	Sequence208Zm6g	
0	Sequence209Zm6g	

Figure S44: Synteny block diagram for *MIR172D* with *Zea mays* as reference. The first column shows duplication depth at each gene locus, second column shows the genes in reference chromosomes and the following is aligned collinear blocks where only match genes are displayed. The alignment among non-anchor genes is discarded in the output and denoted by '||' in the multi-alignment of gene orders. Zm6 - *Zea mays* chr 6.

cells MDPI

Duplication depth	Reference chromosome	Collinear blocks
0	Sequence210At3g	
0	Sequence211At3g	
0	Sequence212At3g	
0	Sequence213At3g	
0	Sequence214At3g	
0	Sequence215At3g	
0	Sequence216At3g	
0	Sequence217At3g	
0	Sequence218At3g	
0	Sequence219At3g	
0	Sequence220At3g	
0	Sequence221At3g	
0	Sequence222At3g	
0	Sequence223At3g	
0	Sequence224At3g	
0	Sequence225At3g	
0	Sequence226At3g	
0	Sequence227At3g	
0	Sequence228At3g	
0	Sequence229At3g	
0	Sequence230At3g	
0	Sequence231At3g	
0	Sequence232At3g	
0	Sequence233At3g	
0	Sequence234At3g	
0	Sequence235At3g	

Figure S45: Synteny block diagram for *MIR172D* with *Arabidopsis thaliana* as reference. The first column shows duplication depth at each gene locus, second column shows the genes in reference chromosomes and the following is aligned collinear blocks where only match genes are displayed. The alignment among non-anchor genes is discarded in the output and denoted by '||' in the multi-alignment of gene orders. At3 - *Arabidopsis thaliana* chr 3.
